# Long-term tolerability and effectiveness of eptinezumab in Japanese adults with chronic migraine: results of the 60-week open-label SUNSET trial

**DOI:** 10.1186/s10194-025-02214-w

**Published:** 2025-11-22

**Authors:** Takao Takeshima, Daisuke Danno, Noboru Imai, Keisuke Suzuki, Anders Ettrup, Sidsel Jensen, Mette Krog Josiassen, Aurélia Mittoux, Yasuhiko Matsumori

**Affiliations:** 1 Headache Center, Department of Neurology, Tominaga Hospital, Osaka, Japan; 2 Japanese Red Cross Shizuoka Hospital, Shizuoka, Japan; 3 Department of Neurology, Dokkyo Medical University, Tochigi, Japan; 4 H. Lundbeck A/S, Copenhagen, Denmark; 5 Sendai Headache and Neurology Clinic, Sendai, Japan

**Keywords:** Chronic migrainef, Effectiveness, Eptinezumab, Japan, Long-term, Patient-reported outcomes, Safety

## Abstract

**Background:**

In the phase 3 SUNRISE trial conducted in a predominantly Asian population with chronic migraine (CM), eptinezumab (100 or 300 mg) met the primary and all key secondary endpoints and was well-tolerated. The open-label SUNSET extension trial was conducted in Japanese participants from SUNRISE and evaluated long-term safety, tolerability, and effectiveness of eptinezumab.

**Methods:**

After completing the 12-week double-blind period of SUNRISE, the first 160 Japanese participants enrolled in SUNSET and 159 received eptinezumab every 12 weeks for 60 weeks. At the SUNSET baseline visit (also SUNRISE Week 12), all participants received an infusion of eptinezumab 100 mg. For those with a < 50% reduction in monthly migraine days (MMDs) during SUNSET Weeks 1–12 relative to baseline values obtained in SUNRISE, the dose of eptinezumab was increased to 300 mg at SUNSET Week 12. The primary outcome was safety and tolerability (based on treatment-emergent adverse events); secondary outcomes were maintenance of migraine-preventive effect and impact on health-related quality of life (e.g, Patient Global Impression of Change [PGIC], patient-identified most bothersome symptom [PI-MBS], and Migraine-specific Work Productivity and Activity Impairment [WPAI:M]). Baseline values reported are from the SUNRISE trial.

**Results:**

Among 159 treated participants, 141 (88.7%) completed SUNSET. Evaluable participants were mostly female (87.4%), with a mean age of 41.9 years and a mean of 17.1 baseline MMDs. No new safety signals were identified, and few participants had a serious adverse event (3.1%), event leading to withdrawal (4.4%), or infusion interruption/termination (5.0%). MMD reductions from the lead-in placebo-controlled trial were maintained during SUNSET, and just over 35% of participants had ≥50% reduction in MMDs from Week 24 onwards. PGIC scores showed that ~50% of participants were very much or much improved from Week 24 onwards. From the lead-in placebo-controlled trial, PI-MBS scores and WPAI:M domain scores for presenteeism, work productivity loss, and activity impairment showed sustained improvement over 60 weeks.

**Conclusions:**

Long-term (60 weeks) treatment with eptinezumab was found to maintain reductions in migraine frequency and severity from the placebo-controlled lead-in trial, with no new safety concerns. These results confirm the effectiveness of eptinezumab in Japanese participants with CM to reduce the burden of migraine and improve the ability to undertake daily life activities.

**Trial Registration:**

ClinicalTrials.gov (Identifier: NCT05064371; date of registration: September 22, 2021)

**Supplementary Information:**

The online version contains supplementary material available at 10.1186/s10194-025-02214-w.

## Graphical Abstract

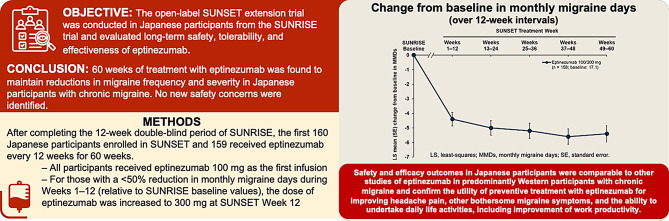

## Background

In Japan, migraine is ranked as the fourth most common cause of years lived with disability [1], with a prevalence estimated to be between 8.6% and 11.0% [2, 3], Migraine has substantial negative impact on daily functioning, quality of life, and life course in Japanese adults [4–6]. One recent cross-sectional trial reported that Japanese individuals with migraine have higher rates of absenteeism, presenteeism, work productivity impairment, and total activity impairment compared with matched controls, and lower health-related quality of life (HRQoL) in terms of mental, physical, and social characteristics [7]. Further, data from the OVERCOME (Japan) population-based survey showed the rate of presenteeism to be 34.3%; work productivity loss, 36.2%; and total daily activity impairment, 35.9% among respondents with migraine, with rates rising to ≥50% in those with chronic migraine (CM) [6, 8]. Results from OVERCOME (Japan) also found that HRQoL was lowest in respondents with CM and that 51% of this subgroup experienced moderate or severe disability due to their disease [6].

Despite the heavy burden of migraine, multiple Japanese studies have reported that medical care and treatment remain insufficient: 43–81% of people with migraine have never consulted a physician about their disease [6, 9, 10], and up to 90% have never used a preventive medication [6]. Preventive medications available in Japan include calcium-channel blockers, antiepileptics, and antidepressants, as well as monoclonal antibodies (mAbs) targeting the calcitonin gene-related peptide (CGRP) [11, 12], with the latter available as of 2021 and recommended in patients for whom ≥1 prior preventive treatment failed to work. Topiramate and onabotulinumtoxinA, which are standard treatments for CM in Europe and the United States [13, 14], are not covered by health insurance in Japan [11]. Although there has been a small increase in prescribing of preventive treatments in recent years, from 16.1% in 2018 to 26.4% in 2022, the majority of people with migraine rely on over-the-counter medications, or are prescribed triptans or non-specific anti-inflammatory drugs [9, 15].

There appear to be several barriers to effective migraine care in Japan. These include stigma and/or a wish to hide migraine (particularly from employers) [16], concern by patients that doctors will not take them seriously or that their symptoms are not severe enough to warrant a consultation [6, 10], and a lack of awareness of migraine and its treatment on the part of both people with migraine and physicians [9, 10, 17]. Although these barriers do not appear to have changed significantly over the past 30 years [18], the approval and introduction of new preventive treatments such as anti-CGRP mAbs appear to have increased recognition and understanding of the availability and benefits of preventive treatment for migraine, including CM, resulting in rising prescribing rates [15]. Nonetheless, greater utilization of preventive treatment options for migraine within Japan remains an important unmet need [8].

The humanized anti-CGRP mAb eptinezumab is administered by intravenous (IV) infusion every 12 weeks and has been shown to be an effective and safe migraine-preventive treatment for both episodic migraine (EM) and CM at doses of 100 or 300 mg in American and European people with migraine [19–23]. Data from short and long-term clinical trials and real-world studies have shown that eptinezumab is able to improve patient well-being, reduce most bothersome migraine-associated symptoms, decrease rate of absenteeism, increase rate of presenteeism, and provide high overall satisfaction with effectiveness [24–28].

To evaluate the efficacy and safety of eptinezumab 100 mg and 300 mg for the preventive treatment of migraine in an Asian population with CM, a large-scale phase 3 clinical trial was conducted (SUNRISE), followed by an open-label extension trial in Japanese participants (SUNSET). In SUNRISE, eptinezumab demonstrated statistically significant reductions in monthly migraine days (MMDs) versus placebo, beginning on Day 1 and sustained through Week 12; between-group differences in mean change from baseline in MMDs (Weeks 1–12) for the total population (*N* = 978) were −2.39 for eptinezumab 100 mg versus placebo (*p* < 0.0001) and −2.73 for eptinezumab 300 mg versus placebo (*p* < 0.0001) and in participants from Japan (*n* = 302), −1.55 for eptinezumab 100 mg versus placebo (*p* = 0.0196) and −1.40 for eptinezumab 300 mg versus placebo (*p* = 0.0342) [29]. The SUNRISE trial showed comparable efficacy to the pivotal PROMISE-2 trial and a similar day 1 onset of effect as seen in previous trials in Western populations (PROMISE-1, PROMISE-2, and DELIVER) [21, 23, 30], confirming the efficacy of eptinezumab in a population predominantly from Asia. Eptinezumab was also well-tolerated over 24-week treatment in SUNRISE, with a safety profile consistent with those observed in prior clinical trials.

Herein, we report the findings from the open-label extension SUNSET trial conducted in Japanese participants with CM, which evaluated the long-term safety, tolerability, and effectiveness of eptinezumab over 60 weeks of treatment.

## Methods

### Trial design

**Fig. 1:**
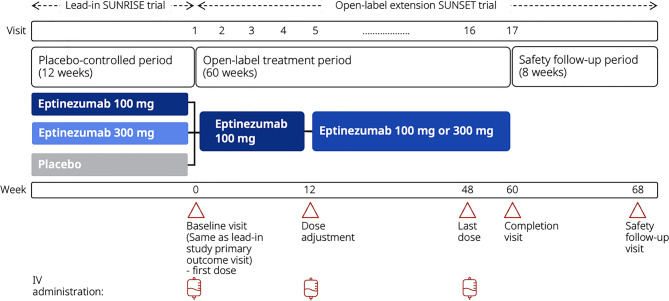
Trial design. Participants with chronic migraine were enrolled in the SUNSET open-label extension trial after completing the SUNRISE double-blind, placebo-controlled trial. Participants in SUNSET attended the clinic every 12 weeks for eptinezumab administration; visits occurring between the onsite attendances were conducted by telephone. In SUNSET, eptinezumab 100 mg was administered to all participants at baseline (Visit 1, Week 0). Participants with ≥50% reduction in MMDs during SUNSET Weeks 1–12 compared with their baseline value in SUNRISE continued to receive eptinezumab 100 mg throughout the trial; those who did not have ≥50% reduction in MMDs during SUNSET Weeks 1–12 compared with their baseline in SUNRISE increased their eptinezumab dose to 300 mg at Visit 4 and for the remainder of the trial. MMDs, monthly migraine days; Q12W, every 12 weeks

SUNSET (NCT05064371) was an open-label, interventional extension trial conducted from September 21, 2021, through June 8, 2024. The design is illustrated in Fig. 1. Participants from Japan with CM, and who were eligible for preventive treatment, were enrolled in the SUNSET open-label extension trial after completing the 12-week SUNRISE double-blind, placebo-controlled trial (published separately [29]). The setting of the trial was clinics or headache centers. Participants in SUNSET received IV eptinezumab every 12 weeks for 60 weeks (5 doses) followed by 8 weeks’ safety follow-up. During the 60-week treatment period, participants were required to complete a daily electronic diary uploaded on a trial device.

At baseline in SUNSET (Visit 1, Week 0), all participants received eptinezumab 100 mg. At Visit 4 (Week 12), the dose was adjusted according to response. Participants with ≥50% reduction in MMDs during SUNSET Weeks 1–12 compared with their baseline value from the SUNRISE trial continued to receive eptinezumab 100 mg at Visit 4 and for the remainder of the trial. Participants who did not have ≥50% reduction in MMDs during SUNSET Weeks 1–12 compared with their baseline value from the SUNRISE trial had their eptinezumab dose increased to 300 mg at Visit 4 and for the remainder of the trial; however, if required due to a treatment-emergent adverse event (TEAE) related to tolerability to eptinezumab, any participant escalated to eptinezumab 300 mg was allowed to reduce their dose back to 100 mg.

The SUNSET trial was designed and conducted in accordance with the Declaration of Helsinki, Good Clinical Practice guidelines, and all applicable regulatory requirements, and was approved by the Ethics Committee or Institutional Review Board at each of the 17 investigational sites.

### Participants

Enrolment in the SUNSET trial was restricted to participants from Japan and required prior completion of the placebo-controlled period of the SUNRISE trial. Eligibility criteria for the SUNRISE trial included a diagnosis of CM at the screening visit (defined per IHS International Classification of Headache Disorders, 3rd edition, guidelines [31]), a history of migraine onset ≥12 months previously, and ≥8 migraine days and ≥15 to ≤ 26 headache days per month during the screening period. A concurrent diagnosis of medication overuse headache was allowed. Prescription and over-the-counter medication for preventive treatment of migraine recommended by a healthcare professional was allowed provided the dose and the regimen were stable for ≥3 months prior to and during the lead-in trial and were expected to be maintained until the completion visit (Week 60) in SUNSET. Full details of the inclusion and exclusion criteria are provided in Additional file 1. SUNSET participants were Japanese adults ages 18–75 years who had migraine onset on or before age 50. All participants in SUNSET provided written informed consent.

### Trial endpoints

The primary objective of the SUNSET trial was to evaluate the long-term safety and tolerability of eptinezumab, measured as the number of participants with TEAEs, classified according to the Medical Dictionary for Regulatory Activities, version 27.0. Vital signs, weight, laboratory values, electrocardiogram (ECG) data, Columbia–Suicide Severity Rating Scale (C-SSRS) [32] scores, and development of specific anti-drug (anti-eptinezumab) antibodies (ADAs) including neutralizing antibodies (NAbs) were also measured.

The main secondary objectives were to evaluate the maintenance of the therapeutic effect of eptinezumab on prevention of migraine and HRQoL. Exploratory objectives were to evaluate the long-term exposure of eptinezumab and to evaluate the maintenance of the therapeutic effect of eptinezumab on work productivity. Effectiveness was assessed using the change from baseline in the number of MMDs; ≥50% and ≥75% migraine responder rates (MRRs); mean Patient Global Impression of Change (PGIC) [33] score; mean patient-identified most bothersome symptom (PI-MBS) [34, 35] score; change from baseline in the 6-item Headache Impact Test (HIT-6) [36] total score; and change from baseline in the EuroQoL EQ-5D-5L visual analog scale (VAS) [37] score. Exploratory endpoints included here are the change from baseline in the Migraine-Specific Quality-of-Life Questionnaire, version 2.1 (MSQ v2.1) [38] domain scores and change from baseline in the Migraine-specific Work Productivity and Activity Impairment questionnaire (WPAI:M) [39, 40] domain scores. Response and change from baseline for all efficacy assessments were calculated according to the baseline values recorded in the SUNRISE trial.

The PGIC and PI-MBS scales range from 1 (very much improved) to 7 (very much worse). PGIC responders were defined as participants who responded with a score of 1 (very much improved) or 2 (much improved). The HIT-6 contains 6 questions, with each item rated as never = 6, rarely = 8, sometimes = 10, very often = 11, and always = 13; thus, the total score ranges from 36 to 78. A score of ≥60 in HIT-6 refers to severely impacted participants. The MSQ v2.1 consists of 14 items covering three domains: role function–restrictive (7 items), role function–preventive (4 items), and emotional function (3 items). Each item is scored on a 6-point scale ranging from 1 (none of the time) to 6 (all of the time). Raw domain scores are summed up and transformed to a 0- to 100-point scale. Higher scores indicate better quality of life. The EQ-5D-5L includes a VAS to rate the overall health state, ranging from 0 (worst imaginable health state) to 100 (best imaginable health state). The WPAI:M assesses activities over the preceding 7 days and consists of two items assessing the number of hours worked and the number of hours missed from work, and two visual numerical scales to assess the impact on productivity at work and the ability to complete normal daily activities.

### Statistical analyses

As this extension trial was not hypothesis-testing, no formal statistical calculations were conducted to set the sample size. The aim was to have safety data encompassing ≥100 participants with at least 1 year of exposure to eptinezumab (defined as receiving 4 consecutive infusions with eptinezumab and having data for at least 365 days). Based on this, the first 160 participants from Japan who completed the SUNRISE trial were enrolled into SUNSET trial. Data from all enrolled participants who received an infusion of trial medication was used to tabulate demographic data and to evaluate safety and tolerability outcomes (all-patients-treated set [APTS]); data from all participants in the APTS who had a valid baseline assessment of MMDs and ≥1 valid post-baseline 4-week assessment of MMDs was used to evaluate effectiveness outcomes (full analysis set [FAS]).

Summary statistics (n, arithmetic mean, standard deviation [SD], median, range) were calculated for continuous variables, and counts and percentages for categorical variables. The change from baseline in the number of MMDs per 4-week interval (Weeks 1–4, 5–8, 9–12, 13–16, 17–20, 21–24, 25–28, 29–32, 33–36, 37–40, 41–44, 45–48, 49–52, 53–56, and 57–60) was modeled using a mixed model for repeated measures (MMRM) using all available data, with month as a factor, baseline score as a continuous covariate, and baseline score-by-month as an interaction term. An unstructured variance structure was used to model the within-participant errors. Change from baseline in MMDs across 3-month intervals was estimated as the average across the three monthly timepoint estimates using equal weights for each 4-week interval. A similar MMRM model was used for the electronic patient-reported outcomes (ePROs), using visit instead of month and using the baseline value of the ePRO as baseline score. For PGIC and PI-MBS where the scale itself represents a change from baseline, baseline score was excluded from the model. MRRs were calculated based on participants who had a ≥50% or ≥75% reduction from baseline in the average MMDs during each interval. Patient-reported outcomes were calculated as scores at each timepoint/interval, or change from baseline at each timepoint/interval. A post hoc analysis was conducted to evaluate the ≥50% MRR over Weeks 13–24 and Weeks 49–60 in participants who had their dose escalated to 300 mg at Week 12 in SUNSET, after not having ≥50% reduction in MMDs for Weeks 1–12 compared to baseline in SUNRISE.

All statistical analyses were conducted using SAS software version 9.4 or later (SAS Institute, Inc., Cary, NC). The trial protocol and statistical analysis plan are available online as Additional file 1 and 2, respectively, in which additional details including the handling of missing data can be found.

## Results

### Participants

**Fig. 2:**
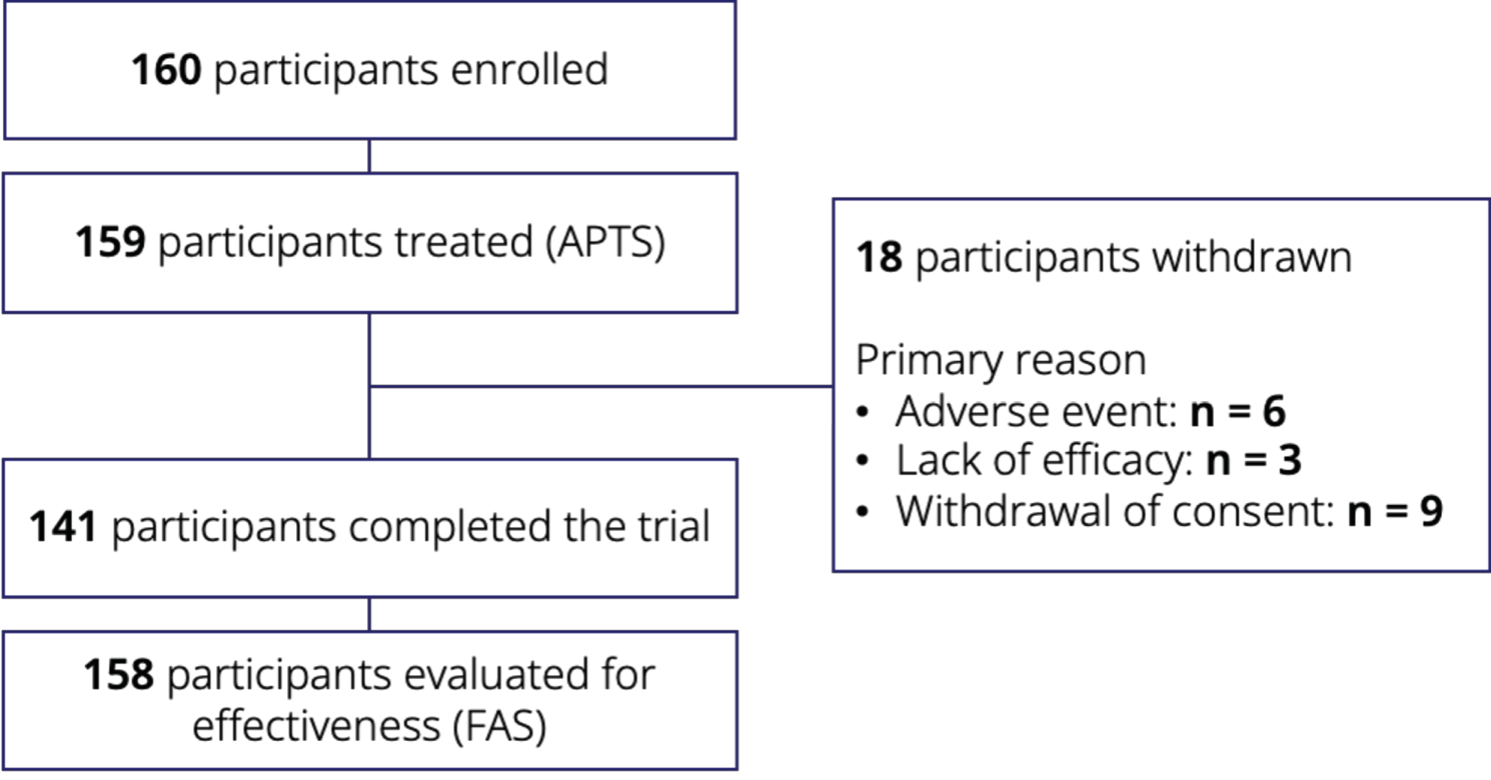
Participant disposition through the SUNSET trial. APTS, all-patients-treated set; FAS, full analysis set

A total of 160 participants from Japan who had completed the SUNRISE trial were enrolled in the SUNSET trial (Fig. 2). One participant did not receive eptinezumab at Visit 1 and was excluded from the APTS; one additional participant was excluded from the FAS since lack of observations did not allow for any post-baseline 4-weekly MMD assessments. During the SUNSET trial, 18 of the treated participants withdrew, with the primary reasons for withdrawal being adverse events (*n* = 6), lack of efficacy (*n* = 3), or revocation of consent (*n* = 9), resulting in a high completion rate (141/159 [88.7%]) for the 60-week treatment period.

Demographics collected at the start of the SUNRISE trial, overall and according to treatment in SUNRISE, are shown in Table 1. The majority of participants in the APTS were female (139/159 [87.4%]), the mean (SD) age was 41.9 (10.6) years, and the mean (SD) number of baseline MMDs (at the start of SUNRISE) for participants in the FAS was 17.1 (3.8). In the APTS, concomitant use of prescription or over-the-counter medication for migraine prevention was reported for 101/159 (63.5%) participants.

**Table 1:** SUNSET participant demographics

		Prior treatment in SUNRISE		
	All SUNSET participants	Eptinezumab 100 mg	Eptinezumab 300 mg	Placebo
	(*N* = 159)	(*n* = 52)	(*n* = 53)	(*n* = 54)
**Sex**, n (%)				
Male	20 (12.6%)	7 (13.5%)	6 (11.3%)	7 (13.0%)
Female	139 (87.4%)	45 (86.5%)	47 (88.7%)	47 (87.0%)
**Age** (years)				
Mean (SD)	41.9 (10.6)	43.4 (10.9)	39.5 (10.7)	42.7 (9.9)
≤35, n (%)	45 (28.3%)	12 (23.1%)	21 (39.6%)	12 (22.2%)
> 35, n (%)	114 (71.7%)	40 (76.9%)	32 (60.4%)	42 (77.8%)
**MMDs**, mean (SD)^a^	17.1 (3.8)	17.2 (3.6)	16.4 (3.9)	17.7 (3.7)
**Age at migraine diagnosis** (years)				
Mean (SD)	38.5 (10.8)	40.1 (11.2)	35.4 (10.5)	40.1 (10.3)
≤21, n (%)	12 (7.5%)	4 (7.7%)	4 (7.5%)	4 (7.4%)
> 21, n (%)	147 (92.5%)	48 (92.3%)	49 (92.5%)	50 (92.6%)
**Duration of migraine at baseline** (years since onset)				
Mean (SD)	22.0 (11.4)	23.5 (11.7)	20.0 (11.1)	22.6 (11.2)
**Duration of migraine at baseline** (years since diagnosis)				
Mean (SD)	3.5 (6.1)	3.5 (6.9)	4.2 (6.1)	2.8 (5.4)
≤15, n (%)	149 (93.7%)	50 (96.2%)	48 (90.6%)	51 (94.4%)
> 15, n (%)	10 (6.3%)	2 (3.8%)	5 (9.4%)	3 (5.6%)
**Suffer from aura**, n (%)				
No	134 (84.3%)	44 (84.6%)	42 (79.2%)	48 (88.9%)
Yes	25 (15.7%)	8 (15.4%)	11 (20.8%)	6 (11.1%)

Data are from the all-patients-treated set unless otherwise specified

^a^Data are from the full analysis set

MMDs, monthly migraine days; SD, standard deviation

### Exposure and dosing

Among participants in the APTS, the majority (140/159 [88.1%]) received all 5 eptinezumab infusions, with 6 (3.8%) receiving 4 infusions, 4 (2.5%) receiving 3 infusions, 6 (3.8%) receiving 2 infusions, and 3 (1.9%) receiving 1 infusion. Overall, 146 Japanese participants had ≥1 year of exposure to eptinezumab across the SUNRISE and SUNSET trials.

After 12 weeks of treatment in SUNSET, 123/159 (77.4%) participants were escalated to the eptinezumab 300-mg dose. Escalation was based solely on the trial algorithm and was triggered by a <50% reduction in MMDs during SUNSET Weeks 1–12 compared with the baseline value from the SUNRISE trial. For 15 participants, a system error resulted in dose escalation. Two participants who had their dose increased to 300 mg subsequently de-escalated back to the 100-mg dose; one was reduced by administration error and the other due to the occurrence of TEAEs (tonsillitis and coronavirus disease 2019 [COVID-19]).

### Safety and tolerability

Although 133 participants (83.6%) experienced a TEAE, the proportions with serious adverse events, TEAEs leading to withdrawal, and TEAEs leading to infusion interruption were low (5/159 [3.1%], 7/159 [4.4%], and 8/159 [5.0%], respectively) (Table 2). The most common TEAEs were COVID-19 (40/159 [25.2%]) and nasopharyngitis (18/159 [11.3%]). Serious adverse events, each occurring in one participant, were appendicitis (not related to treatment), infectious enteritis (not related to treatment), ulcerative colitis (possibly related to treatment), presyncope (possibly related to treatment), and exacerbation of CM (probably related to treatment). Furthermore, the overall prevalence of ADAs was 21% (33/159) and NAbs, 4% (7/159). At Week 92, none of the participants were ADA-positive, indicating that the ADAs are transient. No new safety signals were identified during long-term treatment with eptinezumab, and there were no clinically relevant findings related to vital signs, weight, laboratory values, ECGs, and C-SSRS scores.

**Table 2:** Summary of TEAEs

	Eptinezumab 100/300 mg
	(*N* = 159)
**Participants with TEAEs**, n (%)	133 (83.6%)
**Participants with SAEs**, n (%)	5 (3.1%)
**Participants with TEAEs leading to withdrawal**, n (%)	7 (4.4%)
**Participants with TEAEs leading to infusion interruption/termination**, n (%)	8 (5.0%)
**Deaths**, n (%)	0
**TEAEs occurring in ≥2% of participants in any group**, n (%)	
COVID-19	40 (25.2%)
Nasopharyngitis	18 (11.3%)
Cystitis	8 (5.0%)
Migraine	8 (5.0%)
Malaise	7 (4.4%)
Nausea	7 (4.4%)
Gastroenteritis	5 (3.1%)
Post-acute COVID-19 syndrome	5 (3.1%)
Abdominal discomfort	4 (2.5%)
Abdominal pain	4 (2.5%)
Abdominal pain upper	4 (2.5%)
Arthralgia	4 (2.5%)
Back pain	4 (2.5%)
Cough	4 (2.5%)
Dizziness	4 (2.5%)
Immunization reaction	4 (2.5%)
Influenza	4 (2.5%)
Pharyngitis	4 (2.5%)
Seasonal allergy	4 (2.5%)
Sinusitis	4 (2.5%)

Data are from the all-patients-treated set (APTS)

COVID-19, coronavirus disease 2019; SAE, serious adverse event; TEAE, treatment-emergent adverse event

### Effectiveness

**Fig. 3:**
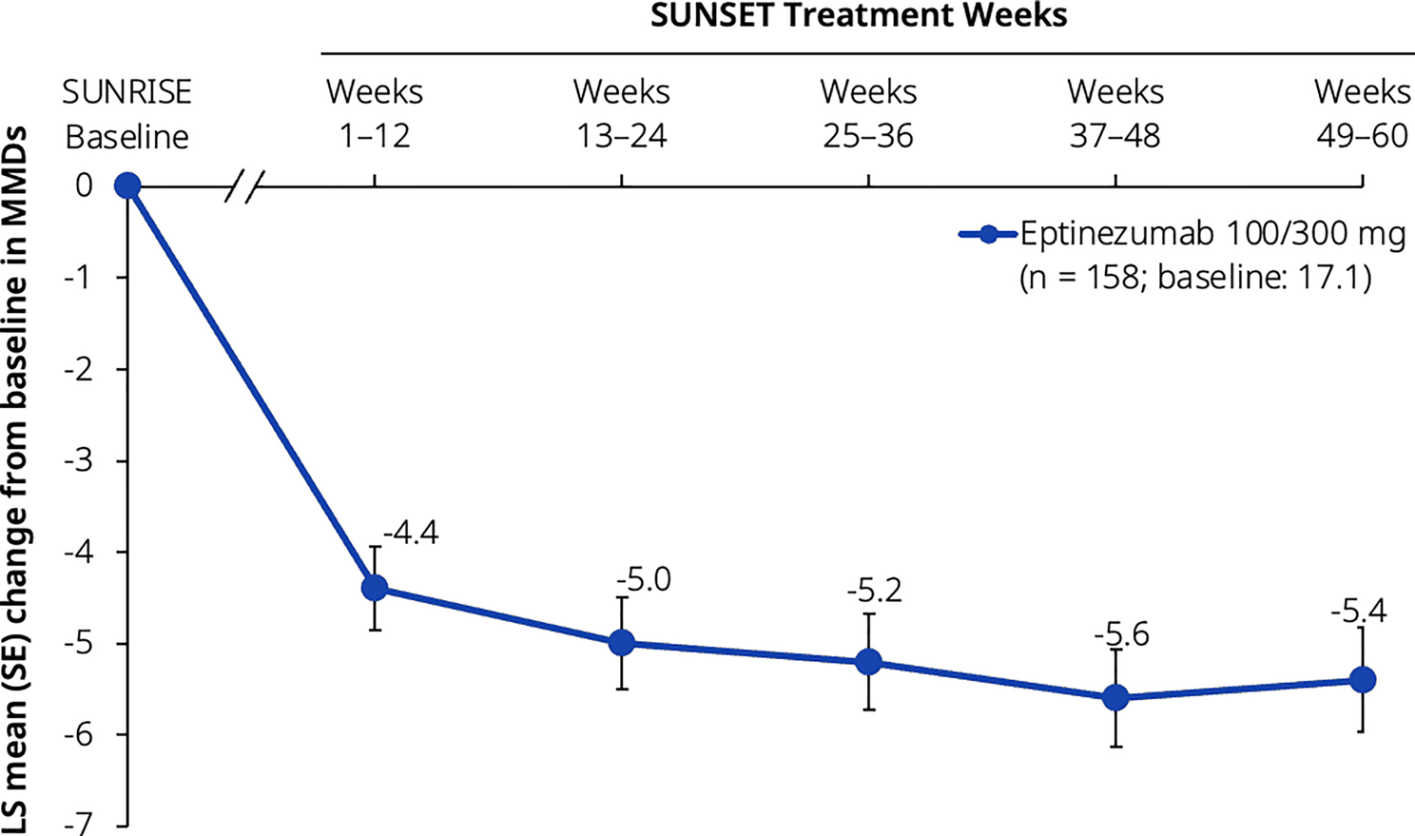
Change from baseline in MMDs over 12-week intervals. Data presented here are from the full analysis set. Baseline values are from the SUNRISE trial. The axis break between SUNRISE baseline and SUNSET Weeks 1–12 represents the 12-week placebo-controlled period in SUNRISE that preceded the SUNSET trial. The estimated LS means were derived from an MMRM with month as a factor, baseline score as a continuous covariate, and baseline score-by-month interaction. From the MMRM, estimates across multiple 4-week intervals were computed using equal weights for each 4-week interval. LS, least-squares; MMDs, monthly migraine days; MMRM, mixed model for repeated measures; SE, standard error

A summary of effectiveness outcomes is provided in Additional file 3. Based on the maintenance of therapeutic effectiveness estimand, the mean change from baseline (i.e., baseline of the SUNRISE trial) in MMDs across Weeks 49–60 was −5.4 days, and across Weeks 1–12 was −4.4 days. Thus, the initial reduction in MMDs was maintained throughout the trial (Fig. 3). Similar effects over time were shown regardless of the lead-in treatment received during the SUNRISE trial (Additional file. 3).

**Fig. 4:**
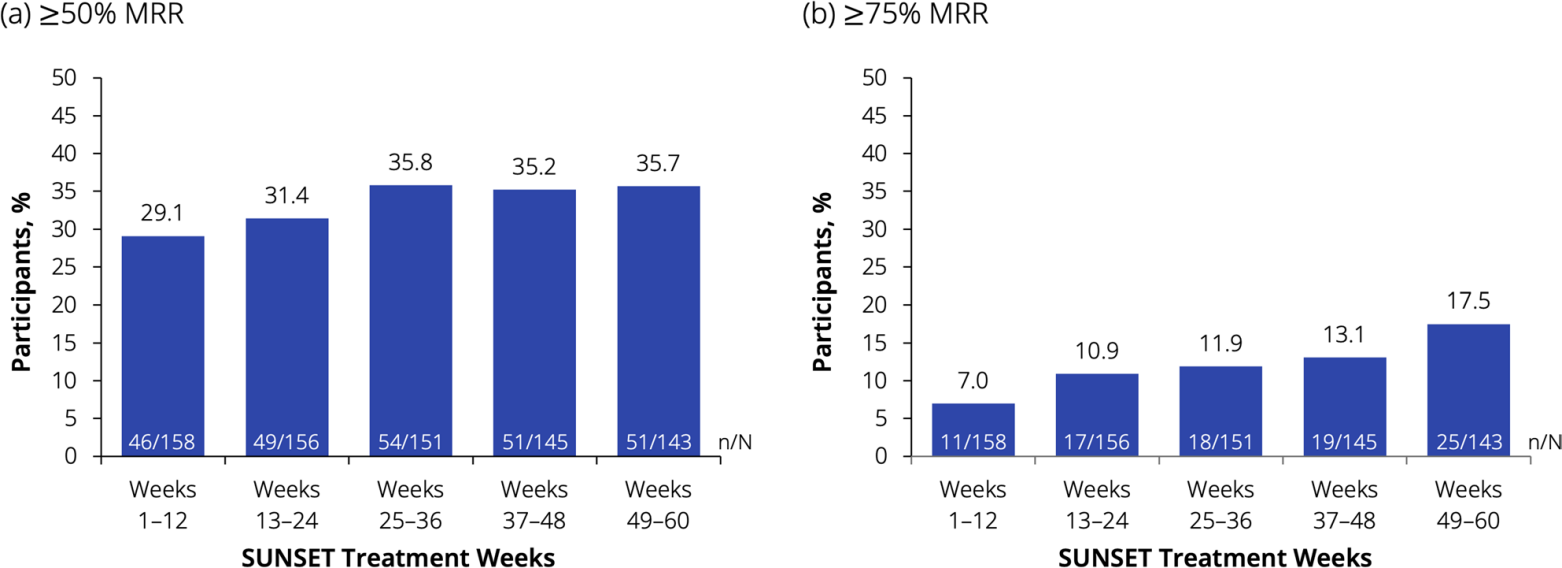
Reduction from baseline in MMDs: (**a**) ≥50% MRR and (**b**) ≥75% MRR. Data presented here are from the full analysis set. Baseline values are from the SUNRISE trial. The ≥50% and ≥75% response variables across the 4-week intervals were calculated as the mean percentage change in MMDs (based on the available monthly values of MMDs). If the MMD value was missing for a given month, the responder status was derived based on the available values. N indicates the number of participants with observations in the interval or at the timepoint. Data represent the percentage of responders. MMDs, monthly migraine days; MRR, migraine responder rate

The proportion of participants with a ≥ 50% reduction from baseline in MMDs was 29% across Weeks 1–12. This initial reduction in MMDs was also maintained throughout the trial (36% across Weeks 49–60) (Fig. 4a). During the 3-month intervals from Week 13 onwards, more than 10% of participants had ≥75% reduction in MMDs, rising to 17.5% during Weeks 49–60 (Fig. 4b). The proportion of participants with a ≥50% reduction from baseline in MMDs was 13.0% (14/108) over Weeks 13–24 and 18.8% (19/101) over Weeks 49–60 in participants who had their dose escalated to 300 mg dose at Week 12, after not having ≥50% response over Weeks 1–12.

**Fig. 5:**
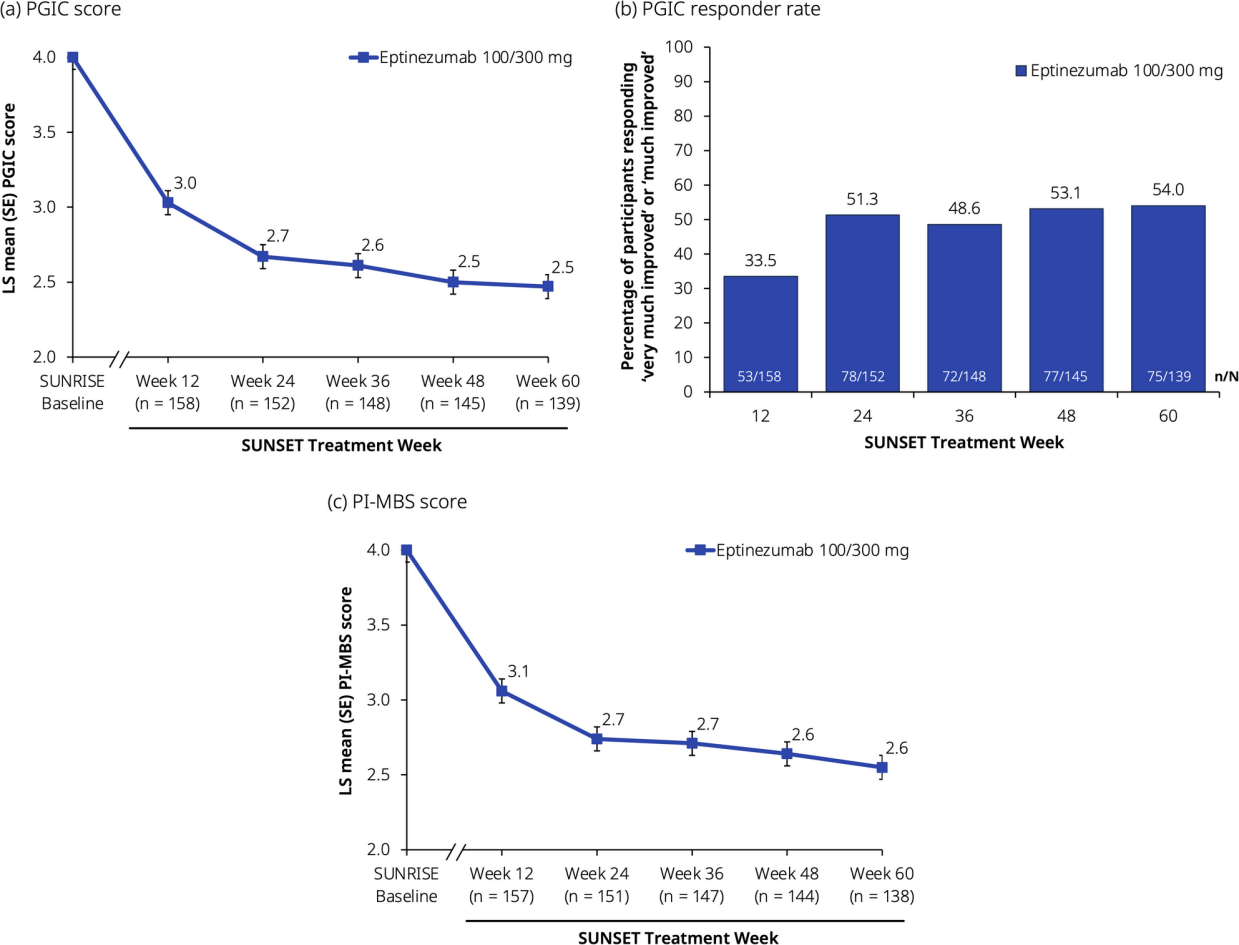
Other secondary endpoints: (**a**) PGIC score, (**b**) PGIC responder rate, and (**c**) PI-MBS score. Data presented here are from the full analysis set. Baseline values are from the SUNRISE trial. The axis break between SUNRISE baseline and SUNSET Week 12 represents the 12-week placebo-controlled period in SUNRISE that preceded the SUNSET trial. The PGIC and PI-MBS scales range from 1 (very much improved) to 7 (very much worse). The estimated LS means in (**a**) and (**c**) were derived from a mixed model for repeated measures with visit as a factor. Data in (**b**) represent the percentage of responders (participants who responded with a score of 1 [very much improved] or 2 [much improved]). LS, least-squares; PGIC, Patient Global Impression of Change; PI-MBS, patient-identified most bothersome symptom; SE, standard error

**Fig. 6:**
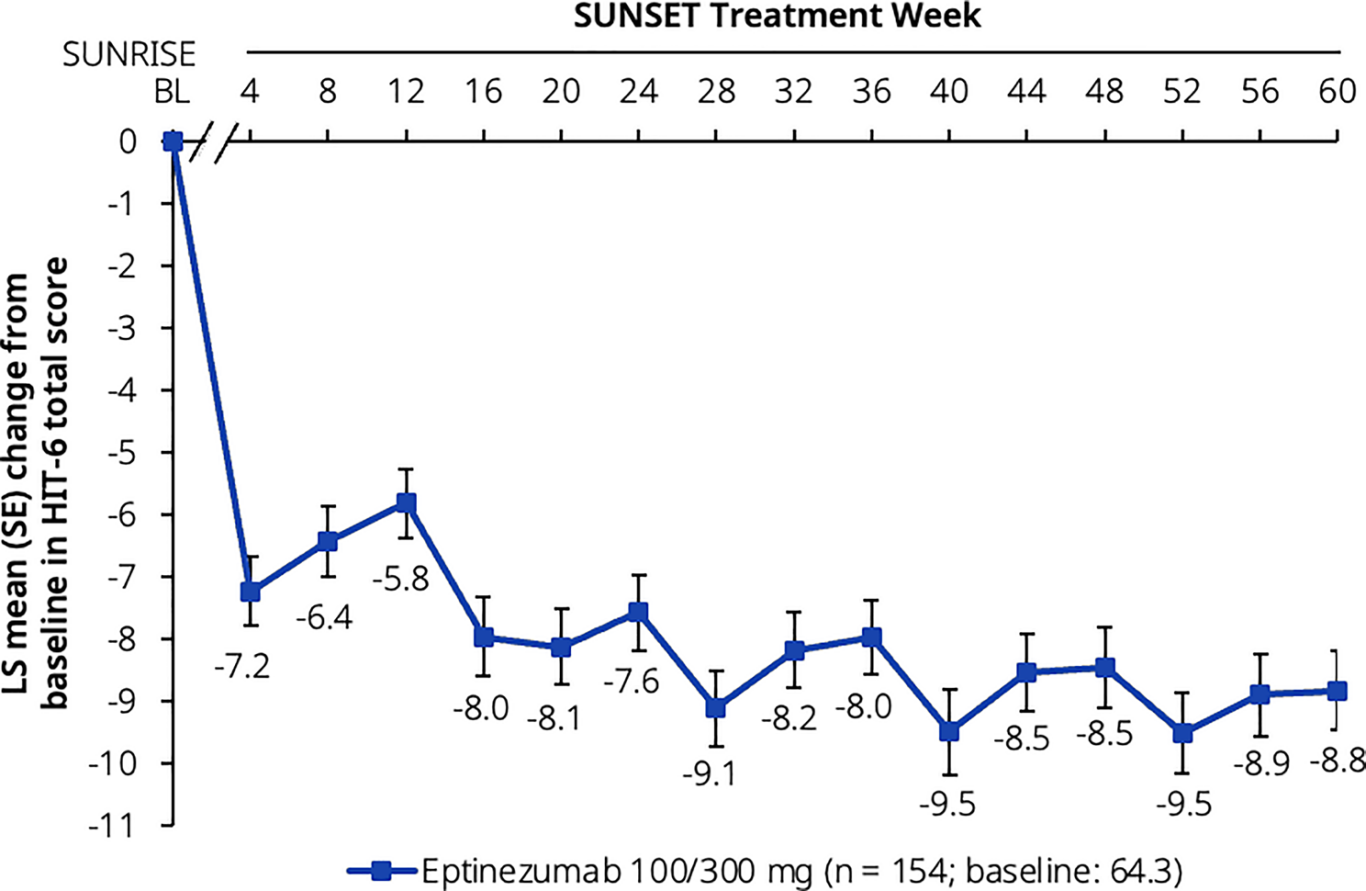
Change from baseline in HIT-6 total score. Data presented here are from the full analysis set. Baseline values are from the SUNRISE trial. The axis break between SUNRISE baseline and SUNSET Week 4 represents the 12-week placebo-controlled period in SUNRISE that preceded the SUNSET trial. The HIT-6 contains 6 questions, with each item rated as never = 6, rarely = 8, sometimes = 10, very often = 11, and always = 13; thus, the total score ranges from 36 to 78. The life impact derived from the total score is described as follows: severe (≥60), substantial (56–59), some (50–55), little to none (≤49). Decrease from baseline indicates improvement. The estimated LS means were derived from a mixed model for repeated measures with visit as a factor, baseline score as a continuous covariate, and baseline score-by-visit interaction. BL, baseline; HIT-6, 6-item Headache Impact Test; LS, least-squares; SE, standard error

Initial reduction was maintained throughout the trial for the mean PGIC score, from Week 12 (3.0) to Week 60 (2.5) (Fig. 5a). From Week 24 onwards, around 50% of participants reported that they were ‘very much improved’ or ‘much improved’ (Fig. 5b). At baseline, among 157 participants who specified their most bothersome symptom on the PI-MBS, the most frequent symptoms were pain with activity (56/157 [35.7%]), nausea (28/157 [17.8%]), and fatigue (23/157 [14.6%]) (Additional file 3). Participants showed sustained improvements in PI-MBS throughout SUNSET (Fig. 5c). At all intervals assessed there were decreases—corresponding to an improvement—from baseline in HIT-6 total score. Initial clinical meaningful improvement ( > 5) was also maintained throughout the trial for the mean decrease in HIT-6 total score from Week 4 (−7.2) to Week 60 (−8.8) (Fig. 6).

The results of the analyses of the MSQ v2.1 subscores showed increases (improvement in the quality of life of the participants) over time from baseline (Additional file. 3). The mean changes from baseline in the individual subscores at Week 12 were 15.6 for role function–restrictive, 12.0 for role function–preventive, and 10.4 for emotional function. At Week 60, the mean changes from baseline were 22.2 for role function–restrictive, 15.6 for role function–preventive, and 17.5 for emotional function.

The results of the analyses of the WPAI:M subscores showed consistent mean decreases, corresponding to improvements for most subscores. The mean changes from baseline at Week 4 were −16.0 for presenteeism, −15.3 for work productivity loss, and −16.5 for activity impairment; these reductions were maintained throughout the trial (−17.2 for presenteeism, −16.0 for work productivity loss, and −21.1 for activity impairment at Week 60). No consistent changes were seen in absenteeism, with scores ranging from 0.2 at Week 4 to 0.6 at Week 60, which may be due to a low baseline score explained by participants working despite migraine symptom burden and leaving little room for improvement (Fig. 6).

**Fig. 7:**
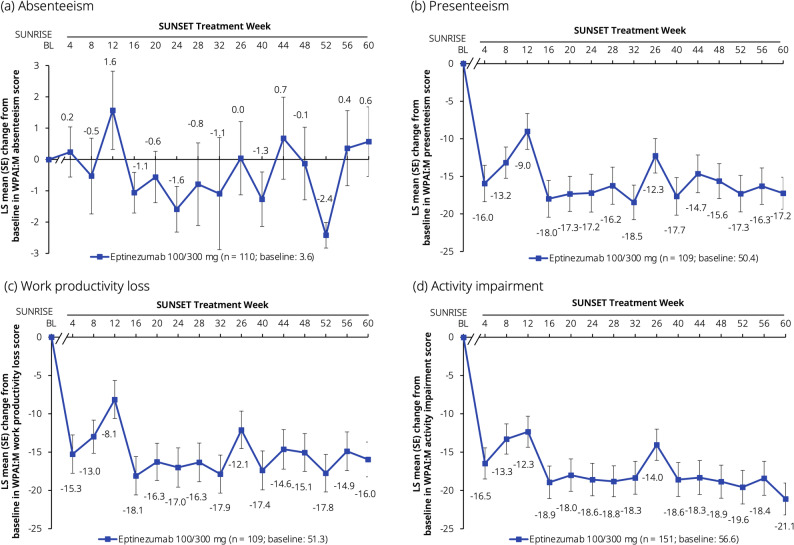
Change from baseline in WPAI:M domain scores: (**a**) absenteeism, (**b**) presenteeism, (**c**) work productivity loss, and (**d**) activity impairment. Data presented here are from the full analysis set. Baseline values are from the SUNRISE trial. The axis break between SUNRISE baseline and SUNSET Week 4 represents the 12-week placebo-controlled period in SUNRISE that preceded the SUNSET trial. The WPAI:M assesses activities over the preceding 7 days and consists of two items assessing the number of hours worked and the number of hours missed from work, and two visual numerical scales to assess the impact on productivity at work and the ability to complete normal daily activities. The estimated LS means were derived from a mixed model for repeated measures with visit as a factor, baseline score as a continuous covariate, and baseline score-by-visit interaction. BL, baseline; LS, least-squares; SE, standard error; WPAI:M, Migraine-specific Work Productivity and Activity Impairment questionnaire

## Discussion

The results from the SUNSET open-label extension trial confirm the safety of eptinezumab in Japanese people with CM and demonstrate maintenance of effect, thereby broadening the evidence base showing the benefits of long-term preventive usage in participants from Japan. Eptinezumab 100 or 300 mg administered every 12 weeks for up to 60 weeks was well-tolerated, maintained the levels of MMD reduction observed in the 12-week SUNRISE trial, and produced improvements in multiple ePROs.

The completion rate for the 60-week long trial was high (88.7%), with few participants discontinuing due to TEAEs or lack of efficacy. While approximately three-quarters of SUNSET participants increased to the eptinezumab 300-mg dose after 12 weeks of treatment, this escalation was automatic—based on the predefined algorithm. Of note, it is unclear if continued improvements were due to the dose escalation or longer eptinezumab treatment.

The safety data from the SUNSET trial were similar to previously reported observations, and no new safety signals relating to long-term treatment of CM with eptinezumab were identified. The most common TEAE was COVID-19 (25.2%), which was unsurprising given the timeframe (2021–2024) during which the trial was conducted. The second most common TEAE was nasopharyngitis (11.3%), which was also one of the most common TEAEs reported in the lead-in SUNRISE trial [29] and in a pooled analysis of eptinezumab clinical trials (from single-dose up to 48 weeks of treatment [41]), and which occurred in 14.1% of participants during the 2-year open-label PREVAIL trial in a Western population with CM [22].

Effectiveness results showed a persistent maintenance of reduction in MMDs over the period of 60 weeks, during which MMDs were maintained or further reduced from the levels seen during the SUNRISE placebo-controlled period. MRRs (≥50% and ≥75%) were also sustained during SUNSET, with more than one-third of participants achieving the ≥50% response rate from Weeks 25–36 onwards, and 17.5% achieving the more stringent ≥75% response rate during Weeks 49–60. Although trials cannot be directly compared due to differences in design, population, and duration, the ≥50% MRR in SUNSET was lower than that observed during the 24-week analysis of the pivotal PROMISE-2 trial in a Western population with CM (where ~60% of participants achieved ≥50% migraine response [42]), but was higher than the 28% who achieved ≥50% reduction in MMDs at Month 6 in a recently reported real-world observational study of eptinezumab conducted in adults with migraine in Singapore [43]. In the analysis according to prior SUNRISE treatment (eptinezumab 100 mg, 300 mg, or placebo), the results indicate broadly similar reductions in MMDs across all groups following eptinezumab treatment during SUNSET. However, the data are complicated by the treatment switches occurring between and within the two clinical trials as well as small sample sizes, and some of the fluctuations observed may be due to random noise rather than a systematic treatment effect.

In addition to MMDs, improvements in most ePROs were maintained across the 60 weeks of treatment in SUNSET. The PGIC data, showing that ~50% of participants were very much or much improved from Week 24 onwards, are comparable with the results from the PROMISE-2 trial in which 52.3% of participants treated with eptinezumab 100 mg and 63.8% treated with 300 mg at Week 12, and 59.3% and 63.6%, respectively, at Week 24 were ‘very much or much improved’ [42]. The difference between responder rates on MMDs and PGIC indicates that a sizable fraction of patients report to be ‘very much or much improved’ without meeting criteria for ≥50% MRR, and suggests that improvements after eptinezumab treatment go beyond reductions in MMDs. Similarly, 61.1% of participants in the PREVAIL trial were ‘very much or much improved’ at Week 4, and maintained or increased these improvements through to Week 48 and beyond [22]. PI-MBS improvements in SUNSET also showed continuous improvements over 60 weeks, similar to the PGIC scores; associations between PGIC and PI-MBS have also been reported previously in the PROMISE-2 trial [34]. Mean baseline HIT-6 total score in this population (64.3) was similar to that reported by respondents in the OVERCOME (Japan) 2nd Study (59.7) [5], indicating ‘severe’ impact on daily life. The mean reduction of 8.8 points at Week 60 indicates that on average SUNSET trial participants had improved from a ‘severe’ impact to ‘some’ impact on daily life. MSQ subscores all showed clinically meaningful improvements over the course of the SUNSET trial. Importantly, improvements in HRQoL and symptoms following eptinezumab treatment also translated into better WPAI:M domain scores for presenteeism, work productivity loss, and activity impairment. The mean baseline score for absenteeism in SUNSET (3.6) was lower than those reported in eptinezumab-treated participants in the DELIVER trial conducted in the United States and Europe (11.4–12.0) [44] and lower than that reported in OVERCOME (Japan) 2nd Study respondents with CM (9.9) [5]; the low baseline score likely contributed to the overall lack of change over time in this domain score. As suggested by the authors of the first OVERCOME (Japan) Study, low rates of absenteeism in the Japanese population could be attributable to a work culture that requires employees to be present even when unwell [8]. However, the mean baseline scores for presenteeism (50.4), work productivity loss (51.3), and activity impairment (56.6) in SUNSET were similar to those in the eptinezumab-treated groups in DELIVER (50.8–53.4, 53.7–57.0, and 58.5–59.1, respectively), and the mean improvements from baseline at Week 24 were also comparable (−17.0 to −18.6 in SUNSET and −19.3 to −24.7 in DELIVER) [44].

Although several trials have reported benefits associated with anti-CGRP mAbs as preventive medications for CM in Asian populations [45–47], most clinical trials have been short-term (12 weeks), and there has been a paucity of long-term outcome data. One trial with erenumab for the treatment of EM or CM has been reported, comprising a 24-week double-blind period plus a 28-week open-label period, in which the mean change from baseline in MMDs in participants with CM was −6.9 days and the proportion of ≥50% responders was 41.7% at Week 52 [48]. The data from the SUNSET trial add further support for the benefits of long-term preventive treatment among Japanese individuals with CM, and should instill physician confidence in prescribing eptinezumab and improve patient receptivity towards the use of preventive medication. It is to be hoped that the availability of these findings will help to overcome some of the current barriers to effective migraine treatment in Japan and will lead to an increase in prescribing rates for preventive medication [8–10, 17].

## Limitations

Limitations of the SUNSET trial include the open-label design lacking a control arm, and that due to the design, dosing was related to level of response, thus precluding any assessment of dose response in the trial. Also, since the dose escalation from 100 mg to 300 mg at Week 12 of the SUNSET trial was done solely based on the whether the participant met ≥50% MRR during Weeks 1–12, this may not reflect the decision to escalate dose in clinical practice.

## Conclusions

The results from the SUNSET trial showed that the decrease in symptoms observed during the SUNRISE trial of eptinezumab were maintained across a 60-week treatment period in Japanese participants with CM. Eptinezumab 100/300 mg was well-tolerated with no new long-term safety concerns, and participants reported decreased MMDs and increased HRQoL compared to the baseline of the SUNRISE trial [29]. Overall, outcomes were comparable with those reported from other studies of eptinezumab in predominantly Western participants with CM and confirm the utility of preventive treatment with eptinezumab for improving headache pain, other bothersome migraine symptoms, and the ability to undertake daily life activities, including improvement of work productivity.

## Electronic supplementary material

Below is the link to the electronic supplementary material.

Additional file 1

Additional file 2

Additional file 3

## Data Availability

In accordance with EFPIA's and PhRMA's “Principles for Responsible Clinical Trial Data Sharing” guidelines, Lundbeck is committed to responsible sharing of clinical trial data in a manner that is consistent with safeguarding the privacy of patients, respecting the integrity of national regulatory systems, and protecting the intellectual property of the sponsor. The protection of intellectual property ensures continued research and innovation in the pharmaceutical industry. Deidentified data are available to those whose request has been reviewed and approved through an application submitted to https://www.lundbeck.com/global/our-science/clinical-data-sharing.

## Abbreviations

ADAs: Anti-drug (anti-eptinezumab) antibodies
APTS: All-patients-treated set
CGRP: Calcitonin gene-related peptide
CM: Chronic migraine
C-SSRS: Columbia–Suicide Severity Rating Scale
ECG: Electrocardiogram
EM: Episodic migraine
ePROs: Electronic patient-reported outcomes
FAS: Full analysis set
HIT-6: 6-item Headache Impact Test
HRQoL: Health-related quality of life
IV: Intravenous
mAbs: Monoclonal antibodies
MMDs: Monthly migraine days
MMRM: Mixed model for repeated measures
MRRs: Migraine responder rates
MSQ v2.1: Migraine-Specific Quality-of-Life Questionnaire, version 2.1
NAbs: Neutralizing antibodies
PGIC: Patient Global Impression of Change
PI-MBS: Patient-identified most bothersome symptom
SD: Standard deviation
TEAE: Treatment-emergent adverse event
VAS: Visual analog scale
WPAI:M: Migraine-specific Work Productivity and Activity Impairment questionnaire
