# Supplementary material for: Long-term tolerability and effectiveness of eptinezumab in Japanese adults with chronic migraine: results of the 60-week open-label SUNSET trial
Source: J Headache Pain. 2025 Nov 22;26(1):275. doi: 10.1186/s10194-025-02214-w (PMC12659320; doi:10.1186/s10194-025-02214-w)
Supplement: Supplementary file 1 — Additional file 1 [file 10194_2025_2214_MOESM1_ESM.pdf]

Clinical Study Protocol

Interventional, open-label, flexible-dose, long-term extension study to evaluate safety of eptinezumab as preventive treatment in patients with migraine in Japan

Eptinezumab

Study No.:	19140B
Sponsor:	H. Lundbeck A/S (Lundbeck)<br>2500 Valby (Copenhagen), Denmark
Edition No.:	2.0<br>(the version No. in the footer is the system version No.)
Date of edition:	10 November 2021

This document is the property of H. Lundbeck A/S and H. Lundbeck A/S is the holder of any and all related intellectual property rights, including, but not limited to, copyrights. This document is confidential. It is not to be copied or distributed to other parties without prior written authorization from H. Lundbeck A/S.

Synopsis – Study 19140B

**Sponsor**<br>H. Lundbeck A/S	**Investigational Medicinal Product**<br>Eptinezumab
**Study Title**<br>Interventional, open-label, flexible-dose, long-term extension study to evaluate safety of eptinezumab as preventive treatment in patients with migraine in Japan	
**Objectives and Endpoints**	
**Primary Objective**<br>• To evaluate the long-term safety and tolerability of eptinezumab	**Safety Endpoints**<br>• Adverse events<br>• Absolute values and changes from baseline in clinical safety laboratory test values, vital signs, weight, and ECG parameter values<br>• Potentially clinically significant clinical safety laboratory test values, vital signs, weight changes, and ECG parameter values<br>• Development of specific anti-eptinezumab antibodies (ADA) including neutralizing antibodies (NAbs)<br>• Columbia-Suicide Severity Rating Scale (C-SSRS) score
**Secondary Objective**<br>• To evaluate the maintenance of the therapeutic effect of eptinezumab on:<br>– prevention of migraine<br>– health-related quality of life	**Secondary endpoints**<br>• Change from baseline in the number of monthly migraine days (MMDs)<br>• Response: ≥50% reduction from baseline in MMDs<br>• Change from baseline in the HIT-6 score<br>• Change from baseline in the Health-Related Quality of Life (EQ-5D-5L) Visual Analogue Scale (VAS) score<br>• Patient Global Impression of Change (PGIC) score<br>• Change from baseline in the Most Bothersome Symptom (MBS) score
**Exploratory Objectives**<br>• To evaluate the long-term exposure of eptinezumab<br>• To evaluate the maintenance of the therapeutic effect of eptinezumab on:<br>– healthcare resource utilization, and<br>– work productivity	**Exploratory Endpoints**<br>• Eptinezumab plasma concentrations during long-term treatment<br>• Change from baseline in the Migraine-Specific Quality of Life (MSQ v2.1) sub-scores (Role Function-Restrictive, Role Function-Preventive, Emotional Function)<br>• Change from baseline in Health Care Resources Utilization (HCRU)<br>• Change from baseline in the Work Productivity and Activity Impairment Questionnaire: Migraine (WPAI:M) sub-scores (Absenteeism, Presenteeism, Work productivity loss, Activity impairment)

Study Methodology

- This is an interventional, multi-site, Open-label Phase III study to be conducted in patients in Japan, to evaluate the long-term safety of eptinezumab in patients with migraine who have completed the Primary Outcome Visit in the Lead-in Study and are eligible for preventive treatment.
- The target population for this study are patients diagnosed with chronic migraine as outlined in the IHS ICHD-3 guidelines, confirmed at screening into the Lead-in Study.
- Investigators and patients will be informed about which treatment the patients received in the Lead-in Study only after the last patient has completed the Open-label Study.
- The Baseline Visit of this study will be the same as Visit 5 (Primary Outcome Visit) in the Lead-in Study.
- The total study duration from the Baseline Visit to the Safety Follow-up Visit is approximately 68 weeks and includes an Open-label Treatment Period (60 weeks) and a Safety Follow-up Period (8 weeks).
- Patients will receive IMP at the Baseline Visit and once every 12 weeks (5 infusions in total) by intravenous infusions of 30 minutes (+15 minutes).
- All patients will receive eptinezumab 100 mg infusion at the Baseline Visit.
- At Week 12 (Visit 4), patients that do not have a treatment response of at least 50% reduction of MMDs as compared to the Baseline Visit of the Lead-in Study will have their eptinezumab dose increased to 300 mg. This dose increase to 300 mg is only done at Visit 4, if applicable. The 50% responder status at Week 12 (Visit 4) will be calculated relative to the Baseline Visit of the Lead-in Study.
- After Visit 4, all patients will continue receiving the same eptinezumab dose for the remainder of the study, except for patients on eptinezumab 300 mg that have tolerability issues (who will be allowed to switch to eptinezumab 100 mg once between Visits 5 to 13 [inclusive] and will remain on the 100 mg dose for the remainder of the study).
- Patients will complete a daily headache eDiary from the Baseline Visit until the Completion/Withdrawal Visit.
- The eDiary data from the 28 days prior to Baseline Visit will be used for generating baseline values for all the efficacy endpoints.
- During the IMP Visits, assessments of safety will be performed before and after the infusion. At these visits, AEs will be collected as well as clinical safety laboratory tests, ECG, weight, vital signs and blood samples for eptinezumab and ADA quantification. On the IMP Visit day, patient-reported outcomes (PROs) must be completed prior to infusion. Patients must ensure to complete eDiary recording of headaches which ended prior to infusion (i.e., for headaches which are ongoing or not yet recorded in the eDiary).
- Patients who complete the study will attend a Safety Follow-up Visit, 20 weeks after the last IMP Visit (date when the last dose of IMP was administered).
- Patients who withdraw, except for those who withdraw their consent, will be asked to attend a Withdrawal Visit as soon as possible and a further Safety Follow-up Visit scheduled 20 weeks after the last IMP Visit (date when the last dose of IMP was administered).
- An independent Safety Data Monitoring Committee (DMC) will regularly monitor the patients' safety data according to the DMC Charter.

The study design is presented in Panel 1 (including the study periods) and the scheduled study procedures and assessments are summarized in Panel 2.

Number of Patients Planned

Approximately 154 patients, recruited from specialist settings, are planned for enrolment in the Lead-in Study in Japan. The aim is that 100 patients complete the Open-label Study.

Target Patient Population

Main Inclusion Criteria

- The patient has completed the Primary Outcome Visit (Visit 5) of the Lead-in Study immediately prior to enrolment into this study.
- The patient is indicated for 60-week preventive treatment of migraine with eptinezumab according to the clinical opinion of the investigator.

Main Exclusion Criteria

- The patient has a SAE or a moderate or severe ongoing AE from the Lead-in Study considered a potential safety risk by the Investigator. This includes an abnormal clinical laboratory test value reported as an AE during the Lead-in Study considered a potential safety risk by the Investigator.
- The patient has a clinically relevant change in vital signs or ECG from the Lead-in Study considered a potential safety risk by the Investigator.
- The following recent and concomitant medications are disallowed or allowed with restrictions with respect to their use prior to or during the study (the list is not comprehensive):
  - Disallowed: any investigational products; other monoclonal antibody treatment targeting the CGRP pathway; oral anti-CGRPs for acute treatment of migraine; CNS- and migraine-related devices (neuromodulation, neurostimulation) or injectable therapies as trigger point injections, extracranial nerve blocks, or facet joint injections; botulinum toxin; monoamine oxidase inhibitors, ketamine, methysergide, methylergonovine, or nimesulide.
  - Allowed with restriction: prescription or over-the-counter medication for preventive - treatment of migraine prescribed or recommended by a healthcare professional; hormonal therapy (for example, contraceptives, hormone replacement therapy); anti-impotence agents; barbiturates (including Fiorinal®, Fioricet®, or any other combination containing butalbital); prescription opiates (including single ingredient or combination medications containing opiates, opioids, tramadol, or tapentadol); and non-pharmacological interventions (e.g., behavioural therapy and acupuncture etc); traditional Chinese Patent medicines.

Investigational Medicinal Product, Doses and Mode of Administration

- Eptinezumab – 100 mg, concentrate for solution for intravenous infusion 100 mg/mL added to 100 mL of 0.9% normal saline, intravenously.
- Eptinezumab – 300 mg, concentrate for solution for intravenous infusion 100 mg/mL added to 100 mL of 0.9% normal saline, intravenously.

The IMP will be administered at the Baseline Visit, and once every 12 weeks by intravenous infusion over 30 minutes (+15 minutes).

Assessment Details

The assessments are summarized in Panel 2. Details for selected assessments which are non-standard/require more explanation/description are provided below. All clinical outcome assessments, used to assess efficacy and pharmacoeconomic information in this study, are patient reported outcomes (PRO).

eDiary

Patients will complete a daily headache eDiary from the Baseline Visit until the Completion/Withdrawal Visit consisting of applications and reports which will be used to derive the migraine and headache endpoints. Ongoing evaluation of eDiary compliance will be performed by the study site based on eDiary reports.

Headache Impact Test (HIT-6)

The HIT-6 (v1.0) is a Likert-type, self-reporting questionnaire designed to assess the impact of an occurring headache and its effect on the ability to function normally in daily life. The HIT-6 contains 6 questions, each item rated from "never" to "always" with the following response scores: never = 6, rarely = 8, sometimes = 10, very often = 11, and always = 13. The total score for the HIT-6 is the sum of each response score and ranges from 36 to 78. The life impact derived from the total score is described as followed: Severe (60-78), Substantial (56-59), Some (50-55), Little to None (36-49). It takes less than 5 minutes to complete the HIT-6 questionnaire.

Most Bothersome Symptom

The Investigator will verbally obtain the most bothersome symptom associated with the patient's migraines during the Screening Visit of the Lead-in Study. Patients will be asked to rate the improvement in this symptom from screening on a 7-point scale identical to the scale used for the PGIC. The MBS areas include: nausea, vomiting, sensitivity to light, sensitivity to sound, mental cloudiness, fatigue, pain with activity, mood changes, and other. It takes less than 5 minutes to complete the MBS.

Migraine-Specific Quality-of-Life Questionnaire (MSQ v2.1)

The MSQ v2.1 is a patient-reported outcome designed to assess the quality of life in patients with migraine. It consists of 14 items covering three domains: role function restrictive; role function preventive; and emotional function. Each item is scored on a 6-point scale ranging from 1 (none of the time) to 6 (all of the time). Scores are obtained for each domain and ranges from 0-100. Higher scores indicate better quality of life. It takes approximately 5-10 minutes to complete the MSQ v2.1.

Health Care Resource Utilization (HCRU)

Migraine-specific healthcare resource utilization information will be collected in terms of outpatient health care professional visits, emergency room visits, hospital admissions, as well as duration of hospital stays. Clinical site personnel and patients will be instructed to capture utilization that takes place outside of visits associated with their participation in the clinical study.

Work Productivity and Activity Impairment: Migraine (WPAI:M)

The WPAI is a patient self-rated scale designed to provide a quantitative measure of the work productivity and activity impairment due to a specific health problem (WPAI:M). The WPAI:M assesses activities over the preceding 7 days and consists of 6 items: 4 items assess the number of hours worked, the number of hours missed from work due to the patient's condition, or due to other reasons, and 2 visual numerical scales to assess how much the patient's condition affects their productivity at work and their ability to complete normal daily activities. It takes approximately 5 minutes to complete the WPAI:M.

Statistical Methodology

- The following analysis set(s) will be used for the analyses:
  - *all-patients-enrolled set* (APES) – all patients who have completed the Lead-in Study and are enrolled into the Open-label Study
  - *all-patients-treated set* (APTS) – all patients in the APES who received IMP in the Open-label Study
  - *full-analysis set* (FAS) – all patients in the APTS who had a valid baseline MMD, based on the eDiary data from the last 28 days of the Lead-in Study, and at least one post-baseline observation of MMD
- Analysis of safety endpoints:
  - Safety endpoints will be descriptively summarised by previous treatment and overall. The summaries of changes from baseline will be presented relative to both the baseline in the Open-label Study and the baseline in the Lead-in Study.
- Analysis of the secondary endpoints:
  - Details on derivation and imputations of days with missing or incomplete eDiary data will be described in the *Statistical analysis Plan* (SAP).
  - Secondary endpoints will be descriptively summarised by previous treatment and overall.

    For exploratory purpose, the changes in MMDs by 12-week intervals will be modelled using mixed model repeated measures (MMRM) including visit as a fixed factor, baseline MMDs as covariate and the interaction of baseline MMD and visit.

Sample Size Considerations

No formal sample size calculations have been performed. The study is planned to enrol patients in Japan who have completed the Lead-in Study and fulfilled the entry criteria for the Open-label Study.

Panel 1 Study Design

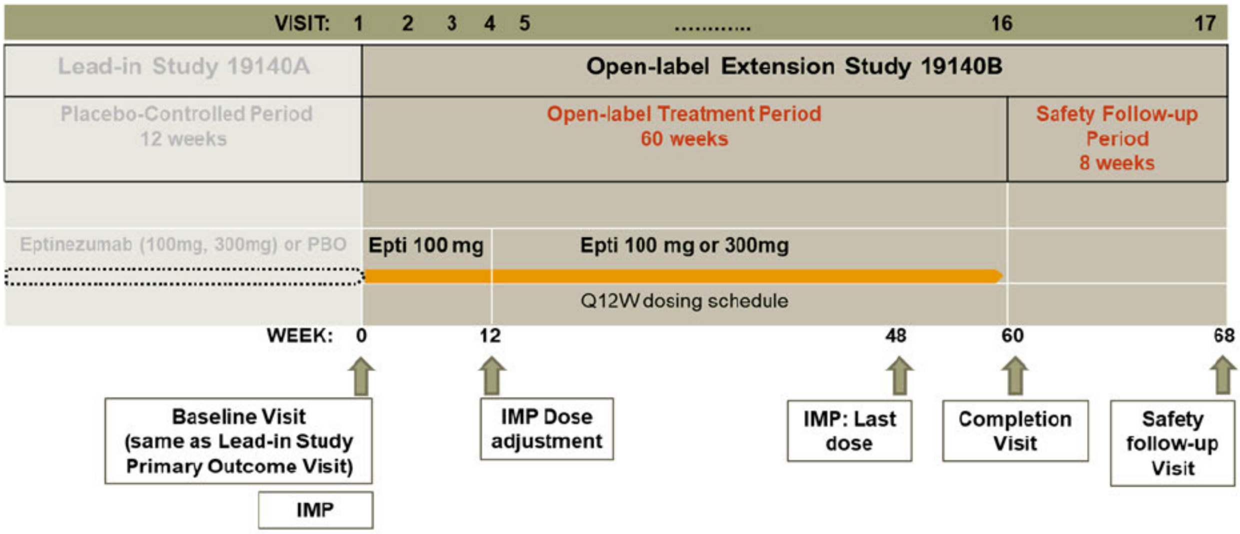

The study consists of an open-label treatment of 60 weeks (5 infusions), and a Safety Follow-up Period (8 weeks). IMP (eptinezumab 300 mg) will be administered by intravenous infusion at the Baseline Visit and once every 12 weeks. At Week 60, patients will complete the Completion Visit and will return to the clinic 8 weeks later for a Safety Follow up Visit.

Panel 2 Study Procedures and Assessments

**Visit Name**	**Baseline+IMP**	**Phone Contact[c]**	**Phone Contact[u]**	**IMP**	**Phone Contact[c]**	**Phone Contact[u]**	**IMP**	**Phone Contact[c]**	**Phone Contact[u]**	**IMP**	**Phone Contact[c]**	**Phone Contact[u]**	**IMP**	**Phone Contact[c]**	**Phone Contact[u]**	**Completion**	**Safety Follow-up**	**Withdrawal[d]**
**Visit Number**	**1**	**2**	**3**	**4**	**5**	**6**	**7**	**8**	**9**	**10**	**11**	**12**	**13**	**14**	**15**	**16**	**17**	
**End of Week[a]**	**0**	**4**	**8**	**12**	**16**	**20**	**24**	**28**	**32**	**36**	**40**	**44**	**48**	**52**	**56**	**60**	**68**	
**Visit Window[b] (days relative to nominal visit)**		**±2**	**±2**	**±2**	**±2**	**±2**	**±2**	**±2**	**±2**	**±2**	**±2**	**±2**	**±2**	**±2**	**±2**	**±2**	**±5**	
**Baseline Procedures and Assessments**																		
Signed informed consent	√																	
Demographics (age, sex, race)[s]	√																	
Diagnosis[s]	√																	
Inclusion/exclusion criteria	√																	
**Efficacy Assessments (eDiary and PROs)[e, g]**																		
eDiary daily recording[f]	√[s]	√	√	√	√	√	√	√	√	√	√	√	√	√	√	√[i]		
eDiary compliance check[h]	√[s]	√	√	√	√	√	√	√	√	√	√	√	√	√	√	√		
PGI-C	√[s]			√			√			√			√			√		√
MBS	√[s]			√			√			√			√			√		√
**Pharmacoeconomic Assessments (PROs)[g]**																		
HIT-6	√[s]	√	√	√	√	√	√	√	√	√	√	√	√	√	√	√		√
MSQ v2.1	√[s]			√			√			√			√			√		√
EQ-5D-5L	√[s]	√	√	√	√	√	√	√	√	√	√	√	√	√	√	√		√
HCRU	√[s]	√	√	√	√	√	√	√	√	√	√	√	√	√	√	√		√
WPAI:M	√[s]	√	√	√	√	√	√	√	√	√	√	√	√	√	√	√		√
**Pharmacokinetic Assessments**																		
Blood sampling for eptinezumab quantification[t]	√[k,l,s]			√[k,l]			√[k,l]			√[k,l]			√[k,l]			√		√
**Safety Assessments**																		
Adverse events	√[j,k,l,s]	√	√	√ [j,k,l]	√	√	√ [j,k,l]	√	√	√ [j,k,l]	√	√	√ [j,k,l]	√	√	√	√	√

Visit Name	Baseline+IMP	Phone Contact[c]	Phone Contact[u]	IMP	Phone Contact[c]	Phone Contact[u]	IMP	Phone Contact[c]	Phone Contact[u]	IMP	Phone Contact[c]	Phone Contact[u]	IMP	Phone Contact[c]	Phone Contact[u]	Completion	Safety Follow-up	Withdrawal[d]
**Visit Number**	**1**	**2**	**3**	**4**	**5**	**6**	**7**	**8**	**9**	**10**	**11**	**12**	**13**	**14**	**15**	**16**	**17**	
**End of Week[a]**	**0**	**4**	**8**	**12**	**16**	**20**	**24**	**28**	**32**	**36**	**40**	**44**	**48**	**52**	**56**	**60**	**68**	
**Visit Window[b] (days relative to nominal visit)**		**±2**	**±2**	**±2**	**±2**	**±2**	**±2**	**±2**	**±2**	**±2**	**±2**	**±2**	**±2**	**±2**	**±2**	**±2**	**±5**	
Blood and urine sampling for clinical safety laboratory tests	√[k,s]			√[k]			√[k]			√[k]			√[k]			√		√
Blood sampling for ADA	√[k,s]			√[k]			√[k]			√[k]			√[k]			√	√[v]	√
Vital signs (including body temperature), weight, ECGs	√[k,l,s]			√[k,l]			√[k,l]			√[k,l]			√[k,l]			√		√
Examinations (physical, neurological)	√[m,s]			√[m]			√[m]			√[m]			√[m]			√		√
C-SSRS[n]	√[k,s]			√[k]			√[k]			√[k]			√[k]			√	√	√
**Other Study Procedures and Assessments**																		
IMP dose adjustment				√[w]														
IMP administered (IV infusion)[o]	√[p]			√[p]			√[p]			√[p]			√[p]					

Visit Name	Baseline+IMP	Phone Contact[c]	Phone Contact[u]	IMP	Phone Contact[c]	Phone Contact[u]	IMP	Phone Contact[c]	Phone Contact[u]	IMP	Phone Contact[c]	Phone Contact[u]	IMP	Phone Contact[c]	Phone Contact[u]	Completion	Safety Follow-up	Withdrawal[d]
**Visit Number**	1	2	3	4	5	6	7	8	9	10	11	12	13	14	15	16	17	
**End of Week[a]**	0	4	8	12	16	20	24	28	32	36	40	44	48	52	56	60	68	
**Visit Window[b] (days relative to nominal visit)**		±2	±2	±2	±2	±2	±2	±2	±2	±2	±2	±2	±2	±2	±2	±2	±5	
IMP accountability[q]	√			√			√			√			√					
Concomitant medication (prescription and non-prescription), traditional Chinese medicines, herbal remedies, non-pharmacological interventions, vitamin and mineral supplements	√[k,s]	√	√	√[k]	√	√	√[k]	√	√	√[k]	√	√	√[k]	√	√	√	√	√
Substance use (alcohol, tobacco, caffeine, marijuana)	√[s]	√	√	√	√	√	√	√	√	√	√	√	√	√	√	√		√
eDiary training/reminder	√																	
PRO training/reminder	√																	
eDiary closeout																√		√
Pregnancy test[r]	√[k,s]			√[k]			√[k]			√[k]			√[k]			√	√	√

ADA = anti-drug antibody; C-SSRS = Columbia-Suicide Severity Rating Scale; ECG = electrocardiogram; βhCG = beta-human chorionic gonadotropin; EQ-5D-5L = Euroqol 5 Dimensions; HCRU = Health Care Resource Utilization; HIT-6 = Headache Impact Test; IMP = investigational medicinal product; IV = intravenous; MBS = Most Bothersome Symptom; MSQ v2.1 = Migraine-Specific Quality of Life Questionnaire Version 2.1; PGI-C = Patient Global Impression of Change; PRO = patient-reported outcome; SAE = serious adverse event; WD = withdrawal; WPAI:M = Work Productivity and Activity Impairment: Migraine questionnaire

a All assessments may be completed over a maximum of 2 consecutive days except for PROs (see foot note e below); if so the first day is considered the “visit” day according to the schedule.

b If the date of a clinic visit or phone contact does not conform to the schedule, subsequent visits should be planned to maintain the visit schedule relative to the Baseline Visit.

c Patients will be contacted via phone for eDiary compliance check, to ensure PROs have been completed, and for collection of relevant information such as AEs and concomitant medication.

d Patients who withdraw, except for those who withdraw their consent, will be asked to attend a Withdrawal Visit as soon as possible. A Safety Follow-up Visit is scheduled 20 weeks after their last IMP Visit (date when last dose of IMP was administered).

e PROs which are scheduled in alignment with a clinic visit can be completed at the clinic or in the remote setting within 3 days prior to the scheduled clinic visit date. PROs which are scheduled in alignment with a phone contact must be completed in the remote setting and can be completed on the day or within 3 days prior to the scheduled phone contact date.

f The eDiary assessments will be completed in the remote setting on a daily basis.

g At IMP visits, patients must complete the PRO entries prior to infusion. Patients must ensure to complete eDiary recording of headaches that ended prior to infusion (i.e., for headaches which are ongoing or not yet recorded in the eDiary).

h In addition to the eDiary compliance checks performed at the defined clinic visits and phone contacts, ongoing evaluation of eDiary compliance will be performed by the site (based on eDiary reporting) and more frequent contact with patients may be needed in case of non-compliance.

i The eDiary closeout will take place at the Completion Visit/Withdrawal Visit while the patient is at the site. Details will be provided in a separate training module.

j Infusion Related Reactions must be checked as part of the overall AE collection, during and after infusion and before the patient is discharged from the site.

k Infusion must be preceded by the assessment of vital signs including body temperature, concomitant medications, AEs, ECG, blood sampling (for clinical safety laboratory tests, ADA and a pre-infusion PK sample), urine sampling (for clinical safety laboratory and pregnancy tests) and C-SSRS. Vital signs must be assessed prior to blood sampling.

l Vital signs including body temperature and AEs must be checked after infusion. A post-infusion PK sample must be taken within 1 hour after end-of-infusion. Vital signs must be assessed prior to blood sampling.

m Physical and Neurological examinations for all clinic visits are to be conducted at the discretion of the investigator. If the examinations are conducted at the IMP Visit, then these must be performed prior to the infusion.

n The C-SSRS will be administered by the authorized rater at the clinic.

o An unblinded pharmacist or designee is responsible for receiving, storing and preparing IMP. The pharmacist or designee will not be responsible for other aspects of the clinical study where blinding is necessary.

p Patients must be monitored during the infusion and for a period of 1 hour from the end-of-infusion. Patients will be requested to stay longer should the investigator or designee determine this is clinically warranted.

q A designated unblinded CRA is responsible for the IMP accountability.

r For women of childbearing potential, pregnancy test at the Safety Follow-up Visit is to be conducted using serum β-HCG. At all other visits, urine pregnancy testing will be performed before infusion and in case of a positive finding, further confirmatory testing will be performed via serum β-HCG.

s Assessments performed prior to IMP will be transferred from the Lead-in Study.

t There will be one blood sample before infusion and another sample within 1 hour after end-of-infusion.

u This phone contact is optional, depending on the ongoing evaluation of eDiary compliance (based on eDiary reporting). In case of non-compliance, a phone contact will be required, and sites must ensure PROs have been completed, and relevant information such as AEs and concomitant medication is collected. If the phone contact is not done, the PROs should be completed as scheduled.

v Patients who test positive for ADA will be asked to provide up to two additional blood samples for immunogenicity testing at 12-week intervals (±1 week) for up to 24 weeks.

w Patients that do not have a treatment response of at least 50% reduction of MMDs by Week 12 (Visit 4) as compared to the Baseline Visit of the Lead-in Study will have their eptinezumab dose increased to 300 mg.

Table of Contents

List of Panels **16**

List of Abbreviations and Definitions of Terms **17**

1 Introduction **20**
1.1 Background........ 20
1.1.1 Overview 20
1.1.2 Nonclinical Data 21
1.1.3 Clinical Data 22
1.2 Rationale for the Study........ 24

2 Objectives and Endpoints **24**

3 Study Design **25**
3.1 Overview of the Study Design........ 25
3.2 Rationale for the Study Design........ 27

4 Ethics **28**
4.1 Ethical Rationale........ 28
4.2 Informed Consent........ 29
4.3 Personal Data Protection 30
4.4 Ethics Committees 30

5 Study Population **31**
5.1 Planned Countries and Planned Number of Patients........ 31
5.2 Selection Criteria 31
5.3 Withdrawal Criteria........ 33

6 Investigational Medicinal Product (IMP) **33**
6.1 Treatment Regimen........ 33
6.2 IMP, Formulation, and Strength 33
6.3 Manufacturing, Packaging, Labelling, and Storage of IMP 34
6.4 Method of Assigning Patients to Treatment 34
6.5 IMP Accountability........ 35
6.6 Post-study Access to IMP........ 35

7 Concomitant Medication **35**

8 Study Visit Plan **36**
8.1 Overview 36
8.2 Baseline + IMP Visit (Visit 1) 37
8.2.1 Patient Identification Card........ 38
8.3 Phone Contacts (Visits 2, 3, 5, 6, 8, 9, 11, 12, 14 and 15)........ 38
8.4 IMP visits (Visit 1, 4, 7, 10 and 13) 38
8.5 Completion (Visit 16) or Withdrawal Visit........ 39
8.6 Safety Follow-up Visit (Visit 17)........ 40
8.7 End-of-study Definition 41
8.8 Unscheduled Visits 41

9 Assessments **41**
9.1 Baseline Procedures and Assessments 41
9.1.1 Demographics and Baseline Characteristics........ 41

9.2 Efficacy Assessments ... 42
9.2.1 Clinical Outcome Assessments (COAs) ... 42
9.2.1.1 Use of COA Tools ... 42
9.2.1.2 eDiary ... 43
9.2.1.3 Patient Global Impression of Change (PGIC) ... 43
9.2.1.4 Most Bothersome Symptom (MBS) ... 44
9.2.1.5 External COA Monitoring Oversight ... 44
9.2.1.6 COA Tool Training ... 44
9.3 Pharmacoeconomic Assessments ... 44
9.3.1 Clinical Outcome Assessments (COAs) ... 45
9.3.1.1 Use of COA Tools ... 45
9.3.1.2 Headache Impact Test (HIT-6) ... 45
9.3.1.3 Migraine-Specific Quality of Life Questionnaire, version 2.1 (MSQ v2.1) ... 45
9.3.1.4 Euroqol 5 Dimension – 5 Levels (EQ-5D-5L) ... 46
9.3.1.5 Health Care Resource Utilization (HCRU) ... 46
9.3.1.6 Work Productivity and Activity Impairment: Migraine (WPAI:M) ... 46
9.3.1.7 External COA Monitoring Oversight ... 46
9.3.1.8 COA Training ... 46
9.4 Pharmacokinetic Assessments ... 47
9.4.1 Blood sampling for eptinezumab quantification ... 47
9.5 Safety Assessments ... 47
9.5.1 Adverse Events ... 47
9.5.2 Clinical Safety Laboratory Tests ... 47
9.5.3 Vital Signs ... 49
9.5.4 Weight ... 49
9.5.5 Electrocardiograms (ECGs) ... 49
9.5.6 Physical and Neurological Examinations ... 50
9.5.7 Columbia-Suicide Severity Rating Scale ... 50
9.5.8 Anti-Drug Antibody (ADA) including Neutralizing Antibody (NAb) Assessments ... 51
9.6 Order of Assessments ... 51
9.7 Total Volume of Blood Drawn ... 52
9.8 Treatment Compliance ... 53

10 Adverse Events ... 53
10.1 Definitions ... 53
10.1.1 Adverse Event Definitions ... 53
10.1.2 Adverse Event Assessment Definitions ... 54
10.2 Management of Reactions to Study Drug ... 55
10.3 Pregnancy ... 56
10.4 Recording Adverse Events ... 56
10.5 Reporting Serious Adverse Events (SAEs) ... 56
10.6 Treatment and Follow-up of Adverse Events ... 57
10.7 Study Monitoring Committee(s) ... 58
10.7.1 Data Monitoring Committee (DMC) ... 58

11 Data Handling and Record Keeping ... 58
11.1 Data Collection ... 58
11.1.1 Electronic Case Report Forms (eCRFs) ... 58

11.1.2 Patient Binders 59
11.1.2.1 Use of Patient Binders 59
11.1.2.2 Serious Adverse Event Fallback Forms 59
11.1.3 External Data 59
11.2 Retention of Study Documents at the Site 59
11.2.1 eCRF Data 59
11.2.2 Other Study Documents 60

12 Monitoring Procedures 60

13 Audits and Inspections 60

14 Protocol Compliance 61

15 Study Termination 61

16 Statistical Methodology 61
16.1 Responsibilities 61
16.2 Analysis Sets 62
16.3 Descriptive Statistics 62
16.4 Patient Disposition 62
16.5 Demographics and Baseline Characteristics 62
16.6 Recent and Concomitant Medication 62
16.7 Exposure 63
16.8 Efficacy Analyses 63
16.8.1 General Efficacy Analysis Methodology 63
16.8.2 Analysis of the Secondary Endpoints 63
16.8.3 Analysis of the Exploratory Endpoints 63
16.9 Safety Analyses 64
16.9.1 General Safety Analysis Methodology 64
16.9.2 Analysis of Adverse Events 64
16.9.3 Analysis of Other Safety Endpoints 64
16.10 Interim Analyses 64
16.11 Sample Size and Power 64
16.12 Statistical Analysis Plan 65

17 Clinical Study Report and Publications 65
17.1 Data Ownership 65
17.2 Clinical Study Report 65
17.3 Summary of Clinical Study Results 65
17.4 Publications 65

18 Indemnity and Insurance 65

19 Finance 66
19.1 Site Agreements 66
19.2 Financial Disclosure 66
19.3 Equipment 66

References 67

Appendices

Appendix I Clinical Study Protocol Authentication and Authorization.............................. 70
Appendix II Recent and Concomitant Medication Disallowed or Allowed with Restrictions 72

List of Panels

Panel 1 Study Design...7
Panel 2 Study Procedures and Assessments...8
Panel 3 Objectives and Endpoints...25
Panel 4 Clinical Safety Laboratory Tests..48

List of Abbreviations and Definitions of Terms

β-hCG	beta-human chorionic gonadotropin
ADA	anti-drug antibody
AE	adverse event
ALP	alkaline phosphatase
ALT	alanine aminotransferase
APTS	all-patients-treated set
AST	aspartate aminotransferase
ATC	anatomical therapeutic chemical
BMI	body mass index
BSC	best supportive care
CGRP	calcitonin gene-related peptide
CI	confidence interval
CM	chronic migraine
CNS	central nervous system
CRA	clinical research associate
CRF	case report form
CRO	clinical research organisation
CSR	clinical study report
C-SSRS	Columbia-Suicide Severity Rating Scale
DMC	Data Monitoring Committee
DNA	deoxyribonucleic acid
EC	ethics committee
ECG	Electrocardiogram
eCRF	electronic case report form
EM	episodic migraine
EMA	European Medicines Agency
EQ-5D-5L	Euroqol 5 Dimensions
FAS	full-analysis set
FDA	Food and Drug Administration
hCG	human chorionic gonadotropin
HCRU	health care resource utilization
HDL	high density lipoprotein
HIT-6	headache impact test
HIV	human immunodeficiency virus
IB	Investigator’s Brochure
ICH	International Council for Harmonisation of Technical Requirements for Pharmaceuticals for Human Use
ICMJE	International Committee of Medical Journal Editors

IMP	investigational medicinal product
IRB	institutional review board
IRR	infusion-related reaction
IRT	interactive response technology
IV	intravenous
LDL	low density lipoprotein
Lu	Lundbeck
MBS	most bothersome symptom
MMDs	monthly migraine days
MMRM	mixed model for repeated measurements
MSQ v2.1	Migraine-Specific Quality of Life Questionnaire Version 2.1
NA	not applicable
NAb	neutralizing antibody
OC	observed case(s)
PCS	potentially clinically significant
PGIC	patient global impression of change
PR	specific ECG interval describing atrioventricular conduction
PRO	patient-reported outcome
QP	qualified person
QRS	specific ECG interval describing ventricular depolarization
QT	specific ECG interval describing ventricular depolarization/repolarization
QT_c	heart-rate corrected QT interval
QT_{cF}	heart-rate corrected QT interval using Fridericia's correction formula
RR	specific ECG interval describing the ventricular depolarization/repolarization cycle
SAE	serious adverse event
SAP	Statistical Analysis Plan
SAS	statistical software package from the SAS® Institute
SD	standard deviation
SOC	system organ class
SUSAR	suspected unexpected serious adverse reaction
TEAE	treatment-emergent adverse event
TMF	trial master file
WPAI:M	Work Productivity and Activity Impairment: Migraine

Major Changes Since Last Edition

The following summarizes the major changes since the last edition of this CSP.

Chapter/ Section Number	Chapter/Section Title	Change
3.1	Overview of the Study Design	*Added*: Migraine day definition serving as basis for "responder status". The investigator will be informed about "responder status" at Visit 4.
5.2	Selection Criteria	*Updated:* Inclusion criteria 6 on contraception measures modified to be clearer in accordance with CTFG guidance for double barrier methods
8.2	Baseline + IMP Visit (Visit 1)	*Added:* Clarification regarding PK/ADA sampling at the Baseline Visit.
8.4	IMP visits (Visit 1, 4, 7, 10 and 13)	*Updated:* Specified that details on the calculation of the response and determination of the dose are to be found in the Pharmacy manual.
8.8	Unscheduled Visits	*Added:* Description of unscheduled visits
Appendix	Recent and Concomitant Medication: Disallowed or Allowed with Restrictions	*Deleted:* Anti-inflammatory agents<br><br>*Added:* COVID vaccine guidance added due to COVID pandemic

1 Introduction

1.1 Background

1.1.1 Overview

Migraine is a disabling disorder characterised by headache and often accompanied by nausea, vomiting, photophobia, and phonophobia.[1] Attacks of migraine typically last between 4 and 72 hours, produce significant disability, and recur often without warning, over decades of time. Migraine is more common in women and most prevalent through the 3rd and 4th decades of life, amplifying its impact on family and career development.[2] Migraine is one of the most prevalent neurological disease for which medical treatment is sought, and worldwide, is considered the leading cause of disability for people under the age of 50 and 2nd leading cause of disability worldwide.[3,4] Generally, migraine begins as an episodic disease. Between attacks of migraine, the nervous system returns to a normal (premorbid) state of function. However, approximately 2.5% of people with episodic migraine (EM) will annually transform from episodic to chronic migraine (CM).[5,6,7] For those with chronic migraine, headaches are more frequent and severe, migraine-associated symptoms are generally more severe, and migraine-related life impact and disability are much greater than that observed in episodic migraine. In addition, CM has a greater association with co-morbid conditions such as anxiety, depression, and non-headache pain. The high prevalence of migraine and its associated disability, especially when transformed to CM, are important justifications for developing effective treatments for the prevention of migraine.

Currently pharmacological treatments of migraine include acute treatments and preventive medications. Preventive treatments are used on a sustained basis for periods of months to years to prevent migraine from occurring. Conventional preventive medications belong to different pharmacological categories (e.g., beta blockers, anticonvulsants) and were all initially developed for other conditions. These treatments show little efficacy and often poor tolerability in patients with migraine, resulting in frequent early discontinuation of treatment.[2,8,9,10,11,12,13] Furthermore, preventive treatment can take weeks to months to achieve optimal efficacy.[14,15] Thus there is a need for preventive medications which are more effective and better tolerated than the current standard of care.[16]

Calcitonin gene-related peptide (CGRP) is thought to play an important role in migraine by facilitating the transmission of migraine pain, thereby contributing to the induction of the pronociceptive stage through modulation of the central nervous system (CNS).[17,18]

A large body of evidence has established the CGRP pathway as a potential target for the treatment of migraine. Studies with monoclonal antibodies targeting CGRP or the CGRP receptor have shown that inhibition of CGRP is efficacious in the treatment of CM and EM.[19,20,21]

Eptinezumab is a humanised monoclonal antibody that inhibits the action of calcitonin gene-related peptide (CGRP) and is approved by the Food and Drug Agency (FDA) as the first and only intravenous preventive treatment for migraine. Eptinezumab addresses an unmet medical

need of patients who require preventive treatment of migraine with an early onset of effect. Eptinezumab is administered by IV infusion every 12 weeks offering a route of drug administration with 100% bioavailability that allows for rapid CGRP inhibition, which has been associated with a clinically meaningful migraine preventive effect as soon as the day following the first infusion. Eptinezumab IV infusion administered every 12 weeks by a healthcare professional helps to ensure treatment adherence and delivery of a known quantity of drug with each dose. Results from two placebo-controlled, Phase III studies showed that eptinezumab led to significant reductions in the number of monthly migraine days (MMDs) in patients with EM (ALD403-CLIN-006)[22] and CM (ALD403-CLIN-011).[23] In clinical studies, treatment with eptinezumab has been associated with a robust and clinically meaningful migraine reduction as early as the day after the first infusion, a sustained efficacy throughout the treatment period and an acceptable tolerability profile with low incidences of study drug withdrawal due to adverse events. Results from an open-label study with repeat doses of eptinezumab used as a preventive treatment for migraine in adults with chronic migraine (ALD403-CLIN013) demonstrated an acceptable long-term safety profile. In this study, robust and clinically meaningful reductions in migraine-related life impact and improvements in measures of health-related quality of life were observed after the initial eptinezumab infusion which were maintained or improved after each subsequent quarterly infusion for up to 2 years. The following sections provide a brief overview of the nonclinical and clinical data currently available for eptinezumab. Refer to the current version of the *Investigator's Brochure*[24] for more detailed information.

1.1.2 Nonclinical Data

Data from the comprehensive program of nonclinical studies support the clinical mechanism of action and safety of eptinezumab.

To demonstrate the ability of eptinezumab to block CGRP-driven vasodilation in vivo, several primary pharmacodynamic animal studies were conducted in rat, cynomolgus monkey and rabbit. The administration of eptinezumab was well tolerated at doses up to 100 mg/kg, the highest dose administered, and inhibited increases in dermal blood perfusion induced by either topical capsaicin (rats and monkey) or intradermal β-CGRP challenge (rabbit). The pharmacodynamic effect was dose-dependent and occurred from 0.1 mg/kg. The appropriateness of the nonclinical species has been established in vitro (rat and rabbit). Across species (including human), eptinezumab has a binding affinity in the low picomolar range for α-and β-CGRP and has been shown to functionally inhibit α-and β-CGRP with high specificity.

Intravenous administration of eptinezumab, either as a single- or multiple-dose for 1-month duration up to 100 mg/kg/dose in rats or monkeys, or multiple-dose for 6-months duration up to 150 mg/kg/dose in monkeys, was well tolerated. No mortality or adverse findings attributed to the pharmacological activity of eptinezumab were observed in the single- or repeat-dose studies in rats or cynomolgus monkeys. As determined during a 6-month chronic toxicity in cynomolgus monkeys, the NOAEL of 150 mg/kg/dose supports a 103-fold or 123-fold safety margin by C_{max} or AUC for the highest dose, 300 mg, of eptinezumab administered by IV infusion every 12 weeks in humans.

Overall, following IV administration in the nonclinical studies, eptinezumab exposure was generally dose proportional, and the plasma-concentration profiles were consistent for IV administration with the rapid achievement of C_{max} followed by a mono-exponential decline. The volume of distribution (V_c) for eptinezumab is generally limited to the vascular compartment.[24,25,26]

Eptinezumab is unlikely to interact directly with DNA or other chromosomal material, and under ICH S6(R1) guidance, evaluations for potential genotoxicity were considered unnecessary and were not performed for eptinezumab. Based on extensive evaluation of the literature related to inhibition of CGRP, angiogenesis, and tumor growth as well as the absence of eptinezumab-related proliferative findings from long-term studies in monkeys, no further nonclinical studies addressing the carcinogenic risk are considered necessary.

Eptinezumab is being developed for the prophylaxis of migraine, and a significant proportion of migraineurs are women of childbearing potential.[27] Given the patient population, and in conformance with applicable guidance documents, a complete package of reproductive/development toxicity studies was conducted. In these studies, administration of eptinezumab by intravenous injection to pregnant female rats or rabbits at 75 or 150 mg/kg/dose was well tolerated. No effects on male or female reproductive function or performance, fertility or early embryonic development in rats were observed. No parental effects or evidence of embryo-lethality, fetotoxicity, or teratogenicity in rats or rabbits were observed. There were no effects on the survival, physical development, behaviour or reproductive performance of the F1 generation in the pre- and postnatal development study in rats.

The local tolerance of eptinezumab was assessed following multiple dose studies in rats and cynomolgus monkeys utilizing eptinezumab administered IV. No gross observations including erythema and oedema, or toxicologically significant histological changes at the injection site(s) were noted in either species for any dose route at concentrations up to 100 mg/mL eptinezumab.

Biologics in general have minimal risks regarding drug-drug interactions;[28] therefore, drug interactions with eptinezumab and concomitant medications are not expected, and nonclinical studies evaluating the potential for interactions with drugs that may be co-administered with eptinezumab were not performed.

1.1.3 Clinical Data

The clinical program of eptinezumab is composed of 5 completed studies to date; 4 studies are placebo-controlled (Phase Ib study in frequent EM (ALD403-CLIN-002),[29] Phase II study in CM (ALD403-CLIN-005),[30] PIII study in frequent EM (ALD403-CLIN-006),[22] PIII study in CM (ALD403-CLIN-011)[23] and 1 study is open-label (PIII study in CM (ALD403-CLIN-013)).[31,32] A Phase III study (18903A)[33] is ongoing to assess treatment of eptinezumab in patients experiencing an acute attack of migraine. A further Phase I study is initiated to assess the safety and PK in Japanese healthy volunteers (18899A).

Eptinezumab is administered by 30-minute (+15 minutes) IV infusion, which bypasses extravascular absorption routes and renders 100% bioavailability. The time required to achieve therapeutic concentrations for eptinezumab is rapid and maximum observed plasma concentration (C_{max}) is typically observed at the end of infusion. The low plasma clearance (0.15 L/d) and protracted terminal-elimination half-life ($t_{½}$) of 27 days for eptinezumab support a sustained duration of effect and infrequent, once every 12 weeks, dosing. The mean accumulation ratios based on C_{max} and $AUC_{0\text{-}tau}$ are 1.08 and 1.15, respectively.

Eptinezumab is not metabolized by cytochrome P450 enzymes. Therefore, interactions by eptinezumab with concomitant medications that are substrates, inducers, or inhibitors of cytochrome P450 enzymes are considered unlikely.[34] Nevertheless, the co-administration of eptinezumab in combination with sumatriptan was investigated in Study ALD403-CLIN-001.[24] The co-administration of sumatriptan did not appear to alter the single dose PK of eptinezumab. Similarly, the PK of sumatriptan was not impacted upon by the co-administration of eptinezumab.

Results from the two placebo-controlled Phase III studies showed that eptinezumab at doses of 100 mg or 300 mg administered by IV infusion every 12 weeks (2 infusions) led to significant reductions in monthly migraine days in patients with EM or CM (ALD403-CLIN-006 and ALD403-CLIN-011).[22,23] Both eptinezumab 300 mg and 100 mg groups achieved the primary efficacy endpoint and all key secondary endpoints in the prespecified statistical hierarchy. The therapeutic benefit resulting from administration of eptinezumab for the preventive treatment of migraine in adults is robust and clinically meaningful, as demonstrated by the results of the 75% and 50% migraine responder analyses. Administration of eptinezumab 100 or 300 mg resulted in a rapid, migraine preventive effect that was established on the day after the first infusion and maintained over the 12-week dosing cycle. Both eptinezumab doses were associated with a consistent pattern of statistically significant and clinically meaningful efficacy across these endpoints compared with placebo.

Results from the open-label study with repeat doses of eptinezumab 300 mg administered every 12 weeks (ALD403-CLIN013) demonstrated an acceptable long-term safety profile in patients with chronic migraine. The PK of free eptinezumab were predictable and consistent with prior analyses. The mean $t^{½}$ of ALD403 was 28 days (674 hours), the median t_{max} was observed at the end of infusion (0.72 hours), and steady state was achieved by Week 12. The establishment of robust and clinically meaningful reductions in migraine-related burden and improvement in measures of quality of life was observed within the first month of treatment and was, on average, sustained or improved over each of the 7 subsequent quarterly infusions that comprised 2 years of treatment. There were no new safety signals identified and the overall safety profile of eptinezumab was consistent with that observed in randomized, placebo-controlled phase III studies of eptinezumab in migraine patients and throughout the eptinezumab clinical development program overall.

In the completed eptinezumab clinical studies, the most common adverse reaction in ≥2% of treated patients and ≥2% greater than placebo were nasopharyngitis and hypersensitivity reactions (hypersensitivity reactions were reported with multiple adverse event terms, such as hypersensitivity, angioedema, urticaria, rash, pruritus, and flushing/hot flush). The majority of

these adverse events were categorized as mild to moderate and most hypersensitivity reactions occurred during infusion. The safety of eptinezumab has been evaluated in 2,076 patients with migraine who received at least one dose of eptinezumab, representing 1615 patient-years of exposure. Long term data with eptinezumab is limited; however, 128 patients have been treated with up to 2 years of exposure and no new significant findings have been identified during the long-term follow-up.

The safety findings to date indicate that eptinezumab is well tolerated and demonstrates a favourable risk-benefit profile based on review of nonclinical, clinical, and scientific literature data.

1.2 Rationale for the Study

The Lead-in Study, with the Lundbeck study number 19140A, aims at establishing the efficacy and safety of eptinezumab in Asian patients with migraine eligible for preventive treatment. The Open-label Study will provide supportive long-term data on the safety and tolerability of eptinezumab in Japanese patients with migraine eligible for further preventive treatment. This study aims to collect data from 100 Japanese migraine patients who are treated with eptinezumab for one-year. Additionally, the study will allow further investigation of the maintenance of the therapeutic effect of eptinezumab by including endpoints evaluating health-related quality of life, most bothersome symptoms, as well as work productivity and to further support the impact of preventive treatment beyond the reduction in pain and migraine days. Besides providing 1-year of exposure data for eptinezumab from 100 completed patients that had been exposed to the proposed therapeutic doses in accordance with ICH E1, this study design will also provide information about dose adjustments that can further guide the prescriber.

There is a high unmet need for new preventive treatment options for migraine which are more effective and better tolerated than the current standard of care.[16,35,36,37,38,39] The demonstrated efficacy and tolerability profile of eptinezumab supports its use as a potential therapeutic candidate for patients with migraine eligible for preventive treatment. Unique to eptinezumab's profile is that it is administered as an intravenous infusion and, throughout its clinical development program, has demonstrated clinically meaningful efficacy in the prevention of migraine from Day 1 after dosing, and sustained efficacy throughout the treatment period with no safety issues observed.

In conclusion, a positive benefit-risk profile has been established in the broad population of migraine patients eligible for preventive treatment. This justifies the initiation of the current study to be conducted in Japanese patients, and to confirm that the long-term safety and tolerability profile of eptinezumab in the Japanese population is similar to that seen in the completed studies.

2 Objectives and Endpoints

The study objectives and endpoints are summarized in Panel 3.

Panel 3 Objectives and Endpoints

Objectives	Endpoints
**Primary Objectives**<br>• To evaluate the long-term safety and tolerability of eptinezumab	**Safety Endpoints**<br>• Adverse events<br>• Absolute values and changes from baseline in clinical safety laboratory test values, vital signs, weight, and ECG parameter values<br>• Potentially clinically significant clinical safety laboratory test values, vital signs, weight changes, and ECG parameter values<br>• Development of specific anti-eptinezumab antibodies (ADA) including neutralizing antibodies (NAbs)<br>• Columbia-Suicide Severity Rating Scale (C-SSRS) score
**Secondary Objectives**<br>• To evaluate the maintenance of the therapeutic effect of eptinezumab on:<br>– prevention of migraine<br>– health-related quality of life	**Secondary endpoints**<br>• Change from baseline in the number of monthly migraine days<br>• Response: ≥50% reduction from baseline in MMDs<br>• Change from baseline in the HIT-6 score<br>• Change from baseline in the Health-Related Quality of Life (EQ-5D-5L) Visual Analogue Scale (VAS) score<br>• Patient Global Impression of Change (PGIC) score<br>• Change from baseline in the Most Bothersome Symptom (MBS) score
**Exploratory Objectives**<br>• To evaluate the long-term exposure of eptinezumab<br>• To evaluate the maintenance of the therapeutic effect of eptinezumab on:<br>– healthcare resource utilization, and<br>– work productivity	**Exploratory Endpoints**<br>• Eptinezumab plasma concentrations during long-term treatment<br>• Change from baseline in the Migraine-Specific Quality of Life (MSQ v2.1) sub-scores (Role Function-Restrictive, Role Function-Preventive, Emotional Function)<br>• Change from baseline in Health Care Resources Utilization (HCRU)<br>• Change from baseline in the Work Productivity and Activity Impairment Questionnaire: Migraine (WPAI:M) sub-scores (Absenteeism, Presenteeism, Work productivity loss, Activity impairment)

3 Study Design

3.1 Overview of the Study Design

This study has been designed in accordance with the *Declaration of Helsinki.*[40]

This is an interventional, multi-site, open-label Phase III study to be conducted in patients in Japan, to evaluate long-term safety in patients with migraine who have completed the Primary Outcome Visit in the Lead-in Study and are eligible for preventive treatment.

This study will be conducted in compliance with the protocol, *Good Clinical Practice*,[41] and applicable regulatory requirements.

An overview of the study is presented in Panel 1.

Approximately 154 patients, recruited from specialist settings, are planned for enrolment in the Lead-in Study in Japan. The aim is that 100 patients will complete the Open-label Study.

The target population for this study are patients diagnosed with chronic migraine as outlined in the IHS ICHD-3 guidelines, confirmed at screening into the Lead-in Study.

Investigators and patients will be informed about which treatment the patients received in the Lead-in Study only after the last patient has completed the Open-label Study.

The Baseline Visit of this study will be the same as Visit 5 (Primary Outcome Visit) in the Lead-in Study.

The total study duration from the Baseline Visit to the Safety Follow-up Visit is approximately 68 weeks and includes an Open-label Treatment Period (60 weeks) and a Safety Follow-up Period (8 weeks). All patients will receive eptinezumab 100 mg infusion at the Baseline Visit irrespectively of what dose the patient was allocated to in the Lead-in Study.

At Week 12 (Visit 4), patients that do not have a treatment response of at least 50% reduction of MMDs as compared to the Baseline Visit of the Lead-in Study will have their eptinezumab dose increased to 300 mg. This dose increase to 300 mg is only done at Visit 4, if applicable. The 50% responder status at Week 12 (Visit 4) will be calculated relative to the Baseline Visit of the Lead-in Study. The migraine day definition that serves as basis for the "responder status" (at least 50% reduction of MMDs) will be the same as the definition used for the calculation of the Baseline value of MMDs.

At week 12 (Visit 4) the investigator will be informed about the "responder status" based on information recorded in the patient's eDiary, and automatically calculated relative to the Baseline visit of the Lead-in Study. The investigator will acknowledge and record the change in dose in the eCRF at Visit 4.

After Visit 4, all patients will continue receiving the same eptinezumab dose for the remainder of the study, except for patients on eptinezumab 300 mg that have tolerability issues (who will be allowed to switch to eptinezumab 100 mg once between Visits 5 to 13 [inclusive] and will remain on the 100 mg dose for the remainder of the study).

Patients will complete a daily headache eDiary from the Baseline Visit until the Completion/Withdrawal Visit. The eDiary data from the 28 days prior to Baseline Visit will be used for generating baseline values.

During the IMP Visits, assessments of safety will be performed before and after the infusion. At these visits, AEs will be collected as well as clinical safety laboratory tests, ECG, weight, vital signs and blood samples for eptinezumab and ADA quantification. On the IMP Visit day, patient-reported outcomes (PROs) must be completed prior to infusion. Patients must ensure to complete eDiary recording of headaches which ended prior to infusion (i.e., for headaches which are ongoing or not yet recorded in the eDiary).

Patients who complete the study will attend a Safety Follow-up Visit, 20 weeks after the last IMP Visit (date when the last dose of IMP was administered). Patients who withdraw, except for those who withdraw their consent, will be asked to attend a Withdrawal Visit as soon as possible and a further Safety Follow-up Visit scheduled 20 weeks after the last IMP Visit (date when the last dose of IMP was administered).

An independent Safety Data Monitoring Committee (DMC) will regularly monitor the patients' safety data according to the DMC Charter.

The study design is presented in Panel 1 (including the study periods) and the scheduled study procedures and assessments are summarized in Panel 2.

3.2 Rationale for the Study Design

The purpose of this study is to conduct a dedicated study in Japanese patients, to allow for investigation of safety and tolerability. The study is intended to show the long-term safety following repeated doses of eptinezumab and the maintenance of the therapeutic effect of eptinezumab on prevention of migraine, health-related quality of life, healthcare resource utilization, and work productivity.

The proposed study population consists of patients with CM who are eligible for preventive treatment at entry into the Lead-in Study. This target population corresponds to the CM patient population in the completed phase III study (ALD403-CLIN-011)[23] in which eptinezumab demonstrated efficacy. The patients enrolled in the current study must have completed the Lead-in Study and have an indication of preventive treatment with eptinezumab for up to 1 year as judged by the clinical evaluation of the treating physician. The patients must continuously fulfil the eligibility criteria established in the Lead-in Study with regards to concurrent diseases and concomitant medication.

The sample size for the study is based on the number of patients planned to be recruited from Japan in the Lead-in Study and with the aim that 100 patients are to complete the Open-label Study.

The current study is a classic open label extension design. Consenting patients who complete the Primary Outcome Visit in the Lead-in Study will be switched directly into the Open-label Study and will receive an IMP infusion at this visit, which corresponds to Baseline Visit of this Open-label Study. All patients will receive the recommended approved dose of 100 mg at the first intravenous infusion, at baseline Visit, with possibility of increasing the dose to 300 mg at week 12 infusion, in case of a clinical response less than 50% reduction in MMDs compared to the baseline visit of the lead-in study. After Visit 4, all patients will continue receiving the same eptinezumab dose for the remainder of the study, except for patients on eptinezumab 300 mg that have tolerability issues (who will be allowed to switch to eptinezumab 100 mg once between Visits 5 to 13 [inclusive], and will remain on the 100 mg dose for the remainder of the study).The study duration will ensure 1-year exposure and safety data on both therapeutic doses of eptinezumab, which corresponds to 5 infusions every 12 weeks (week 0, 12, 24, 36, 48). Besides providing 1-year of exposure data for eptinezumab

from 100 completed patients that had been exposed to the proposed therapeutic doses in accordance with ICH E1, this study design will also provide information about dose adjustments that can further guide the prescriber. Furthermore, the study duration will also allow adequate time to investigate the effect of eptinezumab on health-related quality of life, most bothersome symptoms, as well as work productivity and on potential reduction in new migraine attacks. This will demonstrate the impact of preventive treatment beyond the reduction in pain and migraine days and for further insights into the effectiveness profile of eptinezumab.

In general, safety data from short-term and long-term studies with eptinezumab have not raised any clinical safety concerns at a dose of 100 mg or 300 mg, supporting that eptinezumab can be safely used in the current study. However, it cannot be ruled out that other adverse events associated with eptinezumab have not yet been reported. Blood sampling will be required at several time points during the study to evaluate standard safety laboratory parameters. Although there was no evidence of impact of ADA development on efficacy or safety in any of the previous clinical studies, in the current study the ADA response will be assessed at several time points during the study. Patients who test positive for ADA at the Safety Follow-up Visit will be asked to provide up to two additional blood samples for immunogenicity testing at 12-week intervals (±1 week) for up to 24 weeks.

4 Ethics

4.1 Ethical Rationale

The Open-label Study will provide all patients (including those who participated in the placebo arm) of the Lead-in Study with access to active treatment while collecting useful information about long-term safety. Furthermore, the patients will be allowed to continue their current acute and preventive treatment of migraine from the start of the Lead-in Study. Thus, no patient will be denied access to standard treatments during the study.

The patients will be fully informed about the study, including the risks and benefits of their participation in the study.

The patient may withdraw from the study at any time, for any reason, specified or unspecified and without penalty or loss of benefits to which the patient is otherwise entitled. Unscheduled visits can be made, and immediate withdrawal is possible. Throughout the study, signs of suicidal risk will be assessed and the patients at risk will be withdrawn from the study.

In general, safety data both from short-term and long-term studies with eptinezumab have not raised any clinical safety concerns at a dose of 100 mg or 300 mg, supporting that eptinezumab can be safely used in the current study. However, it cannot be ruled out that other adverse events associated with eptinezumab have not yet been reported. To monitor patient safety, safety data will be reviewed on an ongoing basis and evaluated regularly by the Lundbeck Safety Committee. This will ensure that prompt actions are taken if needed. In

addition, an independent DMC will regularly monitor the patients' safety data according to the DMC Charter.

In accordance with *Good Clinical Practice*,[41] qualified medical personnel at Lundbeck or the clinical CRO will be readily available to advise on study-related medical questions. Medical monitoring will be performed throughout the study. Safety data will be reviewed regularly by the Lundbeck Lu IMP Safety Committee to ensure that prompt action is taken, if needed.

In accordance with *Good Clinical Practice*,[41] the investigator will be responsible for all study-related medical decisions.

Based on data from the nonclinical and clinical studies, and in combination with the cautionary measures implemented in the study design, the risks for the patients are considered well controlled and balanced with the potential benefits of the treatment.

4.2 Informed Consent

No study-related procedures may be performed before the investigator has obtained written informed consent from the patient.

It is the responsibility of the investigator, or person designated by the investigator, to obtain written informed consent from the patient. If the informed consent process is delegated, the requirements for the delegates must be documented prior to the start of the study. National laws must always be adhered to when allowing potential delegation. The *Informed Consent Form* will be provided during the Lead-in Study to patients who may be potentially eligible and who wish to participate in the Open-label Study.

The investigator must identify vulnerable patients, that is, patients whose willingness to participate in this study might be unduly influenced by the expectation, regardless of whether it is justified, of benefits associated with participation, or of a retaliatory response from senior members of a hierarchy in case of refusal to participate. Patients thus identified must be excluded from participation in the study.

Prior to obtaining written informed consent, the investigator or a designee must explain to the patients the aims and methods of the study and any reasonably expected benefits and foreseeable risks or inconveniences to the patients.

The patients must be informed:
- that their participation in the study is voluntary and that they are free to withdraw from the study at any time without justifying their decision
- of the possibility of withdrawing consent (section 8.4)
- of their right to request a copy of their personal data from the study via the investigator
- of their right to be informed by the investigator, after the last patient has completed the study and the full study has been reported, about which treatment they received during the Lead-in Study.

- of their right to receive information about the study results from the investigator on the patients' own initiative; the results will be available approximately 1 year after the end of the study

The patients must be informed that persons authorized by Lundbeck and authorized personnel from certain authorities (domestic, foreign, data protection agencies, or ethics committees (ECs) or institutional review boards (IRBs)) may view their medical records. The patients must also be informed that de-personalized copies of parts of their medical records may be requested by authorized personnel from certain authorities (domestic, foreign, data protection agencies, or ECs or IRBs) for verification of study procedures and/or data. The confidentiality of the patients will in all cases be respected.

The patients must be given ample time and opportunity to enquire about details of the study prior to deciding whether to participate in the study. To ensure this, the investigator or designee, should provide information of the study to the patient during the Lead-in study.

It is the responsibility of the investigator to ensure that all questions about the study are answered to the satisfaction of the patients. Prior to allowing a patient to participate in the study, an *Informed Consent Form* must be signed and dated by the patient and signed and dated by the investigator or a designee on the same day. The patients must be given a copy of the written information (Patient Information Sheet) as well as a copy of the signed *Informed Consent Form*.

The consent procedures described above will only be implemented if allowed by local law and regulations and will only be initiated after approval by the relevant ethics committees.

4.3 Personal Data Protection

The data collected in this study will be processed in accordance with the specifications outlined in the Danish Data Protection Act and the European Union legislation[42] to ensure that requirements regarding personal data protection are met. If an external organization will process data on behalf of Lundbeck, a contractual procedure will be signed between Lundbeck or delegate and the external organization to ensure compliance with the above-mentioned legislation.

4.4 Ethics Committees

This study will be conducted only after Lundbeck has received confirmation that the regulatory authorities have approved or confirmed notification of the study and that written approval of the protocol has been granted by the appropriate IRB.

The investigator must not allow any patients to participate in the study before receiving confirmation from Lundbeck or the CRO that the required approvals and/or notifications have been received.

The IRB must be informed when specific types of protocol amendments have been made and written approval must be obtained before implementation of each amendment, if required by local law.

If applicable, interim reports on the study and reviews of its progress will be submitted to the IRB by the investigator at intervals stipulated in its guidelines.

5 Study Population

5.1 Planned Countries and Planned Number of Patients

The study will be conducted in Japan.

Approximately 154 patients, recruited from specialist settings, are planned for enrolment in the Lead-in Study in Japan. The aim is that 100 patients will complete Open-label Study.

5.2 Selection Criteria

All patients in this study will consist of eligible patients who have participated in, and who have completed, the Lead-in Study in Japan and have agreed to subsequently participate in the current study.

The investigators will be notified immediately when the recruitment period comes to an end. Patient selection is based on the inclusion and exclusion criteria listed below.

Patients who meet each of the inclusion criteria at the Baseline Visit and none of the exclusion criteria at the Baseline Visit are eligible to participate in this study.

Inclusion Criteria

1. The patient is able to read and understand the *Informed Consent Form.*
2. The patient has signed the *Informed Consent Form.*
3. The patient has completed the Primary Outcome Visit (Visit 5) of the Lead-in Study immediately prior to enrolment into this study.
4. The patient is indicated for 60-week preventive treatment of CM with eptinezumab according to the clinical opinion of the investigator.
5. The patient is willing and able to attend study appointments within the specified time windows.
6. The patient, if a woman, must:
   - have had her last natural menstruation ≥12 months prior to the Baseline Visit, OR
   - have been surgically sterilized prior to the Baseline Visit, OR
   - have had a hysterectomy prior to the Baseline Visit, OR
   - remain sexually abstinent, when this is in line with her preferred and usual lifestyle, OR

- engage exclusively in same-sex relationships, OR
- agree not to try to become pregnant during the study, AND
- use at least one of the below adequate contraception:
    - combined oral hormonal contraception
    - intrauterine devices (IUD)
    - intrauterine hormone-releasing system (IUS)
    - male condom
    - vasectomized partner.
- The contraception must be used from the Baseline Visit to ≥6 months after the last dose of IMP.

In addition to the inclusion criteria above, the patient needs to have fulfilled the inclusion criteria from the Lead-in study at the time patient was enrolled and have fulfilled the continued contraception requirement throughout the Lead-in study. Apart from this, a re-evaluation of inclusion criteria from the Lead-in study is not required for enrolling in the Open-label Study.

Exclusion Criteria

1. The patient has previously been enrolled in this study.
2. The patient has a SAE or a moderate or severe ongoing AE from the Lead-in Study considered a potential safety risk by the Investigator. This includes an abnormal clinical laboratory test value reported as an AE during the Lead-in Study considered a potential safety risk by the Investigator.
3. The patient has a clinically relevant change in vital signs or ECG from the Lead-in Study considered a potential safety risk by the Investigator.
4. The patient is pregnant or, planning to become pregnant or breastfeeding.
5. The patient takes or has taken recent or concomitant medication that is disallowed or allowed with restrictions (specified in Appendix II) or it is anticipated that the patient will require treatment with at least one of these medications during the study.
6. The patient is, at the Baseline Visit, at significant risk of suicide (defined as answering "yes" to suicidal ideation questions 4 or 5 or answering "yes" to suicidal behaviour on the C-SSRS at the Primary Outcome Visit (Visit 5) of the Lead in Study.
7. The patient has a disease or takes medication that could, in the investigator's opinion, interfere with the assessments of safety, or tolerability, or efficacy, or interfere with the conduct or interpretation of the study.
8. The patient is, in the investigator's opinion, unlikely to comply with the protocol or is unsuitable for any reason.

In addition to the exclusion criteria above, the patient needs to have not fulfilled the exclusion criteria from the Lead-in Study at the time the patient was enrolled. A re-evaluation of exclusion criteria from the Lead-in study is not required for enrolling in the Open-label Study.

5.3 Withdrawal Criteria

A patient must be withdrawn from the study if:

- the patient withdraws his or her consent (defined as a patient who **explicitly** takes back his or her consent); section 8.4 states how the patient's data will be handled
- the patient is lost to follow-up (defined as a patient who fails to comply with scheduled study visits or contact, who has not actively withdrawn from the study, and for whom no alternative contact information is available [this implies that at least two documented attempts have been made to contact the patient])
- the investigator considers it, for safety, lack of efficacy, and/or study compliance reasons, in the best interests of the patient that he or she be withdrawn from treatment
- the patient is at significant risk of suicide (defined as answering "yes" to suicidal ideation questions 4 or 5 or answering "yes" to suicidal behaviour on the C-SSRS at any time during the study
- the patient becomes pregnant
- the patient has a serum ALT or AST value >3 times the upper limit of the reference range and a serum total bilirubin value >2 times the upper limit of the reference range
- the patient has a serum ALT or AST value >5 times the upper limit of the reference range that is confirmed by testing <2 weeks later
- the patient has a QTcF interval >500 ms; the decision to withdraw the patient may be postponed until a repeat ECG is taken, if it is taken within 24 hours

Patients who withdraw will not be replaced.

A patient must be withdrawn from further treatment with IMP if:

- the patient experiences an anaphylactic reaction or another serious and/or severe hypersensitivity reaction to the IMP infusion, as assessed by the investigator. If the event occurs during the infusion, the infusion must be discontinued immediately.

6 Investigational Medicinal Product (IMP)

6.1 Treatment Regimen

Patients will receive IMP at the Baseline Visit and once every 12 weeks (5 infusions in total) with eptinezumab 100 mg at Baseline, and 100 mg or 300 mg at Visit 4, 7, 10, and 13, by intravenous (IV) infusions of 30 minutes (+15 minutes).

6.2 IMP, Formulation, and Strength

The IMP supplied by Lundbeck in this study is:

- Eptinezumab 100 mg/mL (1 mL/vial) as concentrate for solution for intravenous infusion.

100 mg eptinezumab will be dispensed as 1 vial of 100 mg/mL (1 mL/vial), concentrate for solution for intravenous infusion. 1 x 1 mL of 100 mg/mL concentrate for solution for intravenous infusion is added to 100 mL of 0.9% normal saline, intravenously.

300 mg eptinezumab will be dispensed as 3 vials of 100 mg/mL (1 mL/vial), concentrate for solution for intravenous infusion. 3 x 1 mL of 100 mg/mL concentrate for solution for intravenous infusion is added to 100 mL of 0.9% normal saline, intravenously.

Further instructions on preparation and procedures associated with administering the IV can be found in the *Pharmacy Manual* and *Infusion Guidelines*.

6.3 Manufacturing, Packaging, Labelling, and Storage of IMP

The IMP will be manufactured, packaged, labelled, released (by a qualified person [QP]), and distributed in accordance with the principles of Good Manufacturing Practice, under the responsibility of Lundbeck.

The IMP will be provided in single-use vials (as a concentrate for solution for intravenous infusion).

The wording on the labels will be in accordance with Good Manufacturing Practice regarding labelling and national and/or local regulatory requirements. If additional information is to be added when the IMP is dispensed to the patients, this will be clearly stated on the labels, and the investigator will be instructed to do so.

No manipulation, repackaging, or relabelling of IMP is permitted after QP release by Lundbeck, unless a repackaging/relabelling agreement exists, and the documentation is available to Clinical Supply, H. Lundbeck A/S, and, where necessary, new QP releases are made.

The IMP will be identified using a unique kit Medication number.

The IMP must be stored in a safe and secure location, and in accordance with the storage conditions specified on the labels. Please refer to the *Pharmacy Manual* for additional storage and handling procedures.

6.4 Method of Assigning Patients to Treatment

The patients' screening numbers from the Lead-in Study will be used to identify them in the Open-label Study.

Interactive response technology (IRT) will be used to dispense IMP to the patients in this study.

6.5 IMP Accountability

IMP accountability is documented in the IRT.

The investigator and the pharmacist (if applicable) must agree to only dispense IMP to patients enrolled in the study. The investigator or the pharmacist (if applicable) must maintain an adequate record of the receipt and distribution of the IMP. This record must be available for inspection at any time.

6.6 Post-study Access to IMP

Post-study access to the IMP will not be available. Patients in the study will have access to appropriate medical care after they complete or withdraw from the study.

7 Concomitant Medication

Concomitant medication is any medication other than the IMP that is taken during the study, up until the Safety Follow-up Visit.

All ongoing concomitant medications at the Visit 5 (Primary Outcome Visit) of the Lead-in Study will be transferred to the Open-label Study. The concomitant medications that are disallowed or allowed with restrictions during the study are summarized in Appendix II. Some of the restrictions apply up until the Completion Visit (Week 60) after which the investigator should continue to treat the patient as required by the patient's clinical condition. Acute medication for treatment of migraine (triptans, ergotamine, combination of non-opioid analgesics, individual non-opioid analgesics, opioid analgesics and NSAID) is allowed for the entire duration of the study and will be collected in the eCRF and the eDiary. Investigators must ensure the patients are informed to which class of medication(s) their acute treatment(s) belong to.

Details of all concomitant medication (prescription and over-the-counter), traditional Chinese medicines, herbal remedies, non-pharmacological interventions, vitamin and mineral supplements for the treatment of migraine must be recorded in the eCRF. Any changes (including reason for changes) in concomitant medication, traditional Chinese medicines and non-pharmacological interventions must be recorded at each subsequent visit.

For any concomitant medication, traditional Chinese medicines, herbal remedies, non-pharmacological interventions, vitamin and mineral supplements for which the dose has been increased due to worsening of a concurrent disorder after enrolment in the study, the worsening of the disorder must be recorded as an adverse event. For any of these therapies initiated due to a new disorder after enrolment in the study, the disorder must be recorded as an adverse event.

8 Study Visit Plan

8.1 Overview

An overview of the procedures and assessments to be conducted during the study and their timing is presented in Panel 2. Further details are in chapter 9.

The Baseline Visit (Visit 1) will take place at the same visit as Visit 5 (Primary Outcome Visit, end of Week 12) in the Lead-in Study. Thereafter, study visits are divided into clinic visits (Visits 2, 5 and 6) and phone contacts (Visits 3 and 4).

The IMP is administered at the Baseline Visit (Visit 1) and once every 12 weeks until Visit 13 (5 infusions in total). The Completion Visit (Visit 16) is performed 60 weeks after the Baseline Visit. A Safety Follow-up Visit (Visit 17) is performed 8 weeks after the Completion Visit. Patients who withdraw, except for those who withdraw their consent, are asked to attend a Withdrawal Visit as soon as possible and a further Safety Follow-up Visit at 20 weeks after administration of IMP.

At the clinic visits all assessments may be completed over a maximum of 2 consecutive days (except for PROs - see paragraph below); if so, the first day is considered the “visit” day according to the schedule.

If the date of a clinic visit or phone contact does not conform to the schedule, subsequent visits should be planned to maintain the visit schedule relative to the Baseline Visit.

eDiary and PROs will have been provisioned and used by the patients in the Lead-in Study. During the Baseline Visit of this study, patients will be assisted with the continued access to the eDiary and PROs in the current study. Details will be provided in a separate *eDiary and PRO Training Module.*

Patients will record eDiary headache data daily from the Baseline Visit until the Completion/Withdrawal Visit. At each clinic visit and phone contact (i.e., every 4 weeks), a compliance check of eDiary (based on eDiary reporting) will be conducted. Additionally, ongoing evaluation of eDiary compliance will be performed by the site and more frequent contact with patients may be needed in case of non-compliance. See section 9.2.1.2 for further details on eDiary.

Patients will complete the PROs in alignment with clinic visits and phone contacts. PROs scheduled in alignment with a clinic visit can be completed in the clinic or in the remote setting within 3 days prior to the scheduled clinic visit date. PROs which are scheduled in alignment with a phone contact must be completed in a remote setting and can be completed on the day or within 3 days prior to the scheduled phone contact date. See sections 9.2 and 9.3 for further details on PROs.

After completing or withdrawing from the study, the patient must be treated in accordance with usual clinical practice

8.2 Baseline + IMP Visit (Visit 1)

The Baseline Visit must be conducted as a visit to the site and is the same visit as Visit 5 (Primary Outcome Visit, end of Week 12) of the Lead-in Study.

Informed consent must be obtained before any study-related procedures are initiated. It is suggested that the *Informed Consent Form* is provided during the Lead-in Study 19140A to patients who may be potentially eligible and who wish to participate in the Open-label Study.

The investigator's evaluation of the patient's eligibility in this study will be done based on the latest data available in the Lead-in Study. At the Baseline Visit, the investigator must consider clinically relevant change in vital signs, ECG, or clinical laboratory tests from the Lead-in Study which are considered a potential safety risk to the patient.

At the Baseline Visit, inclusion and exclusion criteria review must be done prior to dosing (see section 5.2 for further details on selection criteria). A compliance check of eDiary, based on the last 4 weeks of the Lead-in Study, will be conducted and the patient must be assisted with re-training if necessary.

At the Baseline Visit, the patient must be assisted and trained in the continued use of eDiary and PROs and compliance requirements. Details will be provided in a separate *eDiary and PRO Training Module* (see section 9.2.1.2 for further details on eDiary and sections 9.2 and 9.3 for further details on PROs).

On the Baseline Visit day, patients must ensure to complete eDiary recording of headaches that ended prior to infusion (i.e., for headaches which are ongoing or not yet recorded in the eDiary). See section 9.2.1.2 for further details on eDiary.

PROs which are scheduled in alignment with the Baseline Visit/Visit 5 of the Lead-in study (as per the Lead-in Study protocol) can be completed in the clinic prior to dosing or in the remote setting within 3 days prior to the scheduled Baseline Visit date. The PROs should preferably be completed in the following order; HIT-6, PGI-C, MBS, MSQ v2.1, EQ-5D-5L, HCRU, WPAI:M. It is preferable that the same order of assessments is used per patient and if the scheduled time of the day for the assessments is as consistent as possible across all the study visits. See sections 9.2 and 9.3 for further details on PROs.

Assessments performed prior to IMP infusion will be transferred from the Lead-in Study. Assessments performed during and after IMP infusion (PK sampling within 1 hour after end-of-infusion and safety assessments) are performed according to Panel 2.

At the Baseline Visit the patients will receive a dose of IMP. See section 8.4 for procedures preceding and following IMP administration. Section 6.2 and *Infusion Guidelines* for further instructions on procedures associated with administering the intravenous IMP.

8.2.1 Patient Identification Card

Each patient will be provided with a patient identification card that states, at a minimum, the name of the IMP, the study number, the patient identification number, the investigator's name, and an emergency telephone number providing 24-hour service.

The patient identification card should be returned to the investigator upon completion of the patient's participation in the study.

8.3 Phone Contacts (Visits 2, 3, 5, 6, 8, 9, 11, 12, 14 and 15)

The patient will be contacted via phone for an eDiary compliance check, to ensure that selected PROs have been completed and for collection of relevant information such as AEs and concomitant medication. Phone contacts should be planned to maintain the visit schedule relative to the Baseline Visit.

A compliance check of eDiary data will be conducted and the patient must be assisted with re-training if necessary. See section 9.2.1.2 for further details on eDiary. PROs which are scheduled in alignment with a phone contact must be completed in the remote setting and can be completed on the day or within 3 days prior to the scheduled phone contact date. The PROs should preferably be completed in the following order; HIT-6, EQ-5D-5L, HCRU, WPAI:M. It is preferable that the same order of assessments is used per patient and if the scheduled time of the day for the assessments is as consistent as possible across all the study visits. See sections 9.2 and 9.3 for further details on PROs.

Visit 3, 6, 9, 12 and 15 phone contacts are optional, depending on the ongoing evaluation of eDiary compliance based on eDiary reporting. In case of non-compliance, a phone contact will be required, and sites must ensure PROs have been completed by the patient, and relevant information such as AEs and concomitant medication is collected. If the phone contact is not done, the PROs should be completed as scheduled.

Only site staff trained and listed in the delegation log will conduct phone contacts and hence be allowed to call the patient. Each phone contact must be documented in medical notes and used for source data verification when completing the eCRF and for subsequent monitoring.

8.4 IMP visits (Visit 1, 4, 7, 10 and 13)

The IMP Visits must be conducted as a visit to the site.

On the IMP Visits days, patients must ensure to complete eDiary recording of headaches that ended prior to infusion (i.e., for headaches which are ongoing or not yet recorded in the eDiary). See section 9.2.1.2 for further details on eDiary. A review and compliance check of eDiary data will be conducted and the patient must be assisted with re-training if necessary.

PROs which are scheduled in alignment with the IMP Visits can be completed either in the clinic or in the remote setting within 3 days prior to the scheduled clinic visit date. The PROs

should preferably be completed in the following order; HIT-6, PGIC, MBS, MSQ v2.1, EQ-5D-5L, HCRU, WPAI:M. It is preferable that the same order of assessments is used per patient and if the scheduled time of the day for the assessments is as consistent as possible across all the study visits. See sections 9.2 and 9.3 for further details on PROs.

At the Baseline Visit the patients will receive a dose of IMP. See section 6.2 and *Infusion Guidelines* for further instructions on procedures associated with administering the intravenous IMP.

Prior to IMP infusion:

- Patients must complete the PROs (if completed at the clinic). PROs can be completed at the patient's convenience before or after the pre-infusion blood and urine sampling.
- The following assessments must be conducted: vital signs including body temperature, concomitant medications, AEs, physical and neurological examination (if done at the discretion of the investigator), ECG, blood sampling (for clinical safety laboratory tests, ADA and PK analysis) and urine sampling (for clinical safety laboratory and pregnancy tests) and C-SSRS administration. Vital signs must be assessed prior to blood sampling.
- At visit 4, patients that do not have a treatment response of at least 50% reduction of MMDs by week 12 (Visit 4), compared to the Baseline Visit of the Lead-in Study, will have a dose increase to 300 mg eptinezumab. More information on the calculation of the response and determination of the dose change will be described in the *Pharmacy manual*.
- After Visit 4, all patients will continue receiving the same eptinezumab dose for the remainder of the study, except for patients on eptinezumab 300 mg that have tolerability issues (who will be allowed to switch to eptinezumab 100 mg once between Visits 5 to 13 [inclusive] and will remain on the 100 mg dose for the remainder of the study).

During IMP infusion: IRRs must be checked as part of the overall AE collection. IRRs must be assessed after the AE collection.

After end-of-IMP-infusion and before the patient is discharged from the site:

- Patients must be monitored for at least 1 hour.
- The following assessments must be conducted: vital signs including body temperature, IRRs and AEs. IRRs must be assessed after the AE collection.
- A blood sample for eptinezumab quantification must be taken within 1 hour after end-of-infusion. Vital signs must be assessed prior to blood sampling.

Patients will be requested to stay longer should the investigator or designee determine this is clinically warranted. After the infusion, the patients will be under observation, but not confined to bed, unless the investigator or designee decides, based on the patient's condition, that it is in the best interest of the patient to be confined to bed.

8.5 Completion (Visit 16) or Withdrawal Visit

The Completion Visit must be conducted as a visit to the site. A compliance check of eDiary will be conducted and the patient must be assisted with the closeout of the eDiary. Details will

be provided in a separate *eDiary and PRO Training Module*. See section 9.2.1.2 for further details on eDiary.

PROs which are scheduled in alignment with the Completion Visit can be completed either in the clinic or in the remote setting within 3 days prior to the scheduled clinic visit date. The PROs should preferably be completed in the following order; HIT-6, PGIC, MBS, MSQ v2.1, EQ-5D-5L, HCRU, WPAI:M. It is preferable that the same order of assessments is used per patient and if the scheduled time of the day for the assessments is as consistent as possible across all the study visits. See sections 9.2 and 9.3 for further details on PROs.

Patients who withdraw from the study prior to the Completion Visit will be asked to attend a Withdrawal Visit at the site, if at all possible. The visit must be scheduled as soon as possible after withdrawal and includes the same procedures as the Completion Visit described above, with the addition of recording the withdrawal reason in the eCRF. The patient will be asked to take a pregnancy test. A Safety follow-up visit will be scheduled 20 weeks after the last dose of IMP was administered.

No new information will be collected from patients who withdraw from the study, except information collected in relation to the scheduled Withdrawal Visit or needed for the follow-up of adverse events (see section 10.6).

For a patient who withdraws consent:

- if the patient withdraws consent during a visit and then agrees to it being the final visit, the investigator will complete the visit as a Withdrawal Visit and all the data collected up to and including that visit will be used.
- if the patient withdraws consent during a telephone conversation, the investigator will ask the patient if he or she will attend a Withdrawal Visit. If the patient:
  - agrees to attend a Withdrawal Visit, all the data collected up to and including that visit will be used.
  - refuses to attend a Withdrawal Visit, the investigator should attempt to follow the patient’s safety and future treatment; any information collected will only be recorded in the patient’s medical records.
- if the patient explicitly requests that the patient’s data collected from the time of withdrawal of consent onwards not be used, this will be respected.

8.6 Safety Follow-up Visit (Visit 17)

The safety follow-up must be conducted as a visit to the site. The Safety Follow-up Visit must be conducted 8 weeks after the Completion Visit (i.e., scheduled 20 weeks after last administration of IMP). A Safety Follow-up Visit is conducted to capture adverse events that occur during the Safety Follow-up Period as well as to follow up on the outcome of adverse events ongoing at the end of the Completion Visit/Withdrawal Visit.

For patients who withdraw from the study, except for those who withdraw their consent, will be asked to attend a Withdrawal Visit as soon as possible and a further Safety Follow-up Visit at 20 weeks after the last IMP administration.

Patients who test positive for ADA at the Safety Follow-up Visit will be asked to provide up to two additional blood samples for immunogenicity testing at 12-week intervals (±1 week) for up to 24 weeks.

For patients with a clinically significant out-of-range clinical safety laboratory test value at the Safety Follow-up Visit, further safety follow-up should be scheduled in accordance with usual clinical practice until the value normalizes or stabilizes or a diagnosis or reasonable explanation has been established. Any further safety follow-up after the last protocol-specified contact with the patient will be recorded in the patients' medical records and not in the eCRF; see section 10.6 for details.

For adverse events that were ongoing at the end of the Completion/Withdrawal Visit and that resolved during the Safety Follow-up Period, the stop date must be recorded.

For non-serious adverse events still ongoing at the safety follow-up, the *Ongoing Adverse Event* checkbox on the *Adverse Event Form* must be ticked. SAEs must be followed until resolution or the outcome is known.

The safety follow-up for patients who withdraw consent must be performed, if at all possible; any information collected will only be recorded in the patients' medical records.

8.7 End-of-study Definition

The end of the study for an individual patient is defined as the last protocol-specified contact with that patient. The overall end of the study is defined as the last protocol-specified contact with the last patient ongoing in the study.

8.8 Unscheduled Visits

Unscheduled visits can be completed if required as either site visit or telephone visits. At these visits, clinical safety laboratory tests, ECG, vital signs, physical examination, neurological examination or pregnancy tests can be performed. In case of any additional tests performed not covered by the existing tests specified in the protocol and the eCRF, the results can be reported in connection with an AE reporting (see section 10) or documented in the medical notes, as applicable.

9 Assessments

9.1 Baseline Procedures and Assessments

9.1.1 Demographics and Baseline Characteristics

In order to allow for patient identification, the same screening number from the Lead-in Study will be used for the current study.

Demographic information and baseline characteristics captured during the Lead-in Study will not be re-collected in this study but will be referenced from the Lead-in Study.

As the Baseline Visit will be the same as Visit 5 (Primary Outcome Visit) in the Lead-in Study, the assessments taken in the Lead-in Study will be transferred to this study.

See Panel 2 (footnote s) for demographic information, baseline characteristics and assessments to be transferred from the Lead-in Study.

Substance use (alcohol, tobacco, caffeine and marijuana) will be collected throughout the study at all visits until the Completion Visit/Withdrawal Visit.

9.2 Efficacy Assessments

Efficacy assessments include the eDiary to record daily headache data and PROs (PGIC, MBS).

Patients will record eDiary headache data daily from the Baseline Visit (Visit 1) until the Completion/Withdrawal Visit.

Patients will complete the PGIC and MBS along with the pharmacoeconomic assessment PROs (see section 9.3). The PROs should preferably be completed in the following order; HIT-6, PGIC, MBS, MSQ v2.1, EQ-5D-5L, HCRU, WPAI:M. It is preferable that the same order of assessments is used per patient and if the scheduled time of the day for the assessments is as consistent as possible across all the study visits.

PROs will be completed in alignment with clinic visits and phone contacts (see chapter 8):

- PROs which are scheduled in alignment with a clinic visit: can be completed in the clinic or in the remote setting within 3 days prior to the scheduled clinic visit date. On an IMP Visit day, patients must complete the PROs prior to infusion.
- PROs which are scheduled in alignment with a phone contact: must be completed in the remote setting and can be completed on the day or within 3 days prior to the scheduled phone contact date.

9.2.1 Clinical Outcome Assessments (COAs)

9.2.1.1 Use of COA Tools

The COA tools are the eDiary and PROs, and guidance will be given on how to complete them to the patients by designated site staff (see section 9.2.1.6). Detailed instructions will be provided in a separate *eDiary* and *PRO Training Module.*

The COA tools will be administered in the local language. Only those provided by Lundbeck that have been validated in the language to which they have been translated will be used in this study.

The following COA tools will be used for efficacy assessments:

- eDiary – to assess daily headache and migraine variables, i.e., the number of hours with headache, presence of associated symptoms, and use of acute migraine medications start and stop dates, headache severity.
- PGIC - to assess overall change in the severity of illness following treatment.
- MBS - to assess a migraine-related symptom that is most bothersome for the patient.

9.2.1.2 eDiary

At the Screening Visit of the Lead-in Study, the patient will have been assisted with the provisioning and training of the eDiary by designated site staff. At the Baseline Visit (Visit 1) of this study, the patient must be assisted in the continued use of eDiary and compliance requirements. Patients will be instructed to complete the eDiary daily, from the Baseline Visit until the Completion/Withdrawal Visit. During the Completion/Withdrawal Visit, eDiary close-out must be performed while the patient is on site. Details will be provided in a separate *eDiary Training Module*.

The content of the headache diary is developed on key symptoms and characteristics of migraine. The eDiary consists of applications and reports which will be used to derive the headache and migraine endpoints. For each day, the patient should record if they experienced any headaches. For each experienced headache, the start and stop date and time will be collected. The patient will record further daily information regarding headache characteristics (for instance, headache severity, additional symptoms) and intake of headache/migraine acute medication. Headache items will be assessed with a yes/no response; and severity will be rated as mild, moderate or severe. Additional details regarding the questions that patients will answer can be found in the *eDiary Training Module*.

At each IMP Visit (dosing) day, patients must ensure to complete eDiary recording of headaches that ended prior to infusion (i.e., for headaches which are ongoing or not yet recorded in the eDiary).

On each day during the study until the Completion/Withdrawal Visit, the patient will be asked to record eDiary data for the day.

Site staff will be given access to the eDiary data. Compliance data (based on eDiary reporting) will be made available throughout the study to site staff for review on a regular basis. At each clinic visit and phone contact (i.e., every 4 weeks), a compliance check of eDiary will be conducted. Additionally, ongoing evaluation of eDiary compliance will be performed by the site and more frequent contact with patients may be needed in case of non-compliance. All follow-up with patients regarding eDiary compliance should be documented in the source records.

9.2.1.3 Patient Global Impression of Change (PGIC)

The PGIC is a single patient-reported item reflecting the patient's impression of change in their disease status since the start of the study (that is, in relation to activity limitations,

symptoms, emotions, and overall quality of life). The item is rated on a 7-point scale, where a high score indicate improvement (very much improved; much improved; minimally improved; no change; minimally worse; much worse; very much worse). It takes approximate 1 minute to complete the scale.

9.2.1.4 Most Bothersome Symptom (MBS)

The Investigator will verbally obtain the most bothersome symptom associated with the patient's migraines during the Screening Visit of the Lead-in Study. Patients will be asked to rate the improvement in this symptom from the Screening Visit of the Lead-in Study and continuously throughout the Open-label Study. It will be done on a 7-point scale identical to the scale used for the PGIC. The MBS areas include: nausea, vomiting, sensitivity to light, sensitivity to sound, mental cloudiness, fatigue, pain with activity, mood changes, and other. It takes less than 5 minutes to complete the MBS.

9.2.1.5 External COA Monitoring Oversight

Lundbeck reserves the right to use external quality oversight methods to ensure eDiary compliance and data quality, as well as ensure accurate completion of the COAs. For this study, the CRO will conduct the external data monitoring (to be agreed with the sponsor).

9.2.1.6 COA Tool Training

The COA tools are patient-reported. Therefore, designated site staff will receive guidance on good standards in completion of the COAs, in-order to adequately train the patients on completion of the eDiary and PROs.

COA training will be conducted by the CRO (as agreed with the sponsor). Site staff will complete their designated training curriculum based on their initial qualification status and assigned role. Any exceptions must be discussed and approved by Lundbeck and/or its designee. The training program will also include general COA quality assurance and management guidance.

Only site staff who have adequate experience with migraine and who have received adequate training on good standards in completion of the eDiary and PROs will be authorized to train the patients on completion of eDiary and PROs in the study. Documentation of training will be provided to site staff for archiving in the investigator trial master file (TMF). New eDiary and PRO trainers joining the study must be trained accordingly.

9.3 Pharmacoeconomic Assessments

Pharmacoeconomic assessments include PROs (HIT-6, MSQ v2.1, EQ-5D-5L, HCRU, WPAI:M).

Patients will complete these PROs along with the efficacy assessment PROs (PGIC and MBS, see sections 9.2.1.3 and 9.2.1.4). The PROs should preferably be completed in the following

order; HIT-6, PGIC, MBS, MSQ v2.1, EQ-5D-5L, HCRU, WPAI:M. It is preferable that the same order of assessments is used per patient and if the scheduled time of the day for the assessments is as consistent as possible across all the study visits.

PROs will be completed in alignment with clinic visits and phone contacts:

- *PROs which are scheduled in alignment with a clinic visit* (see Panel 2): can be completed in the clinic or in the remote setting within 3 days prior to the scheduled clinic visit date. On an IMP Visit day, patients must complete the PROs prior to infusion.
- *PROs which are scheduled in alignment with a phone contact* (see Panel 2): must be completed in the remote setting and can be completed on the day or within 3 days prior to the scheduled phone contact date.

9.3.1 Clinical Outcome Assessments (COAs)

9.3.1.1 Use of COA Tools

Refer to section 9.2.1.1 for further information on use of COA tools.

The following COA tools will be used for pharmacoeconomic assessments:

- HIT-6 - to assess the impact of an occurring headache and its effect on the ability to function normally in daily life
- MSQ v2.1 - to assess quality of life related to migraine
- EQ-5D-5L - to assess the overall state of health
- HCRU - to assess migraine-specific healthcare resource utilization
- WPAI:M - to assess overall effect of health on productivity at work and daily activities

9.3.1.2 Headache Impact Test (HIT-6)

The HIT-6 (v1.0)[43] is a Likert-type, self-reporting questionnaire designed to assess the impact of an occurring headache and its effect on the ability to function normally in daily life. The HIT-6 contains 6 questions, each item is rated from "never" to "always" with the following response scores: never = 6, rarely = 8, sometimes = 10, very often = 11, and always = 13. The total score for the HIT-6 is the sum of each response score and ranges from 36 to 78. The life impact derived from the total score is described as followed: Severe (≥60), Substantial (56-59), Some (50-55), Little to None (≤49). It takes less than 5 minutes to complete the HIT-6 questionnaire.

9.3.1.3 Migraine-Specific Quality of Life Questionnaire, version 2.1 (MSQ v2.1)

The MSQ v2.1[44] is a patient-reported outcome designed to assess the quality of life in patients with migraine. It consists of 14 items covering three domains: role function restrictive (7 items); role function preventive (4 items); and emotional function (3 items). Each item is scored on a 6-point scale ranging from 1 (none of the time) to 6 (all of the time). Raw domain

scores are summed and transformed to a 0 to 100-point scale. Higher scores indicate better quality of life. It takes approximately 5-10 minutes to complete the MSQ v2.1.

9.3.1.4 Euroqol 5 Dimension – 5 Levels (EQ-5D-5L)

The EQ-5D-5L[45] is a patient-reported assessment designed to measure the patient's well-being. It consists of 5 descriptive items (mobility, self-care, usual activities, pain/discomfort, and depression/anxiety) and a visual analogue scale (VAS) of the overall health state. Each descriptive item is rated on a 5-point index ranging from 1 (no problems) to 5 (extreme problems) and a single summary index (from 0 to 1) can be calculated. The VAS ranges from 0 (*worst imaginable health state*) to 100 (*best imaginable health state*). It takes approximately 5 minutes to complete the EQ-5D-5L.

9.3.1.5 Health Care Resource Utilization (HCRU)

Migraine-specific healthcare resource utilization information will be collected in terms of outpatient health care professional visits, emergency room visits, hospital admissions, as well as duration of hospital stays. Clinical site personnel and patients will be instructed to capture utilization that takes place outside of visits associated with their participation in the clinical study.

9.3.1.6 Work Productivity and Activity Impairment: Migraine (WPAI:M)

The WPAI[46] is a patient self-rated scale designed to provide a quantitative measure of the work productivity and activity impairment due to a specific health problem (WPAI:M). The WPAI:M assesses activities over the preceding 7 days and consists of 6 items: 4 items assess the number of hours worked, the number of hours missed from work due to the patient's condition, or due to other reasons, and 2 visual numerical scales to assess how much the patient's condition affects their productivity at work and their ability to complete normal daily activities. It takes approximately 5 minutes to complete the WPAI:M.

9.3.1.7 External COA Monitoring Oversight

See section 9.2.1.5

9.3.1.8 COA Training

See section 9.2.1.6

9.4 Pharmacokinetic Assessments

9.4.1 Blood sampling for eptinezumab quantification

The blood samples for eptinezumab quantification in plasma will be drawn in accordance with Panel 2. The blood sampling and handling procedures are described in the *Laboratory Specification Manual*.

The bioanalysis will be performed under the responsibility of Department of Bioanalysis - Bioanalysis, H. Lundbeck A/S, according to a protocol approved by Lundbeck.

The results may be subjected to a pharmacokinetic analysis, that will be reported separately.

9.5 Safety Assessments

9.5.1 Adverse Events

The patients will be asked a non-leading question (for example, “How do you feel?”, “How have you felt since your last visit?”) at each visit, starting at the Baseline Visit. Adverse events (including worsening of concurrent disorders, new disorders, and pregnancies) either observed by the investigator or reported spontaneously by the patient will be recorded, and the investigator will assess the seriousness and the intensity of each adverse event and its relationship to the IMP. Results from relevant tests and examinations, such as clinical safety laboratory tests, vital signs, and ECGs, or their corresponding conditions will also be recorded as adverse events if considered by the investigator to be clinically significant.

See chapter 10 for further information on adverse events.

9.5.2 Clinical Safety Laboratory Tests

The clinical safety laboratory tests are listed in Panel 4.

Panel 4 Clinical Safety Laboratory Tests

**Haematology**	**Liver**[b]	**Urine**[e]
B-haemoglobin [HGB]<br>B-erythrocyte count [RBC]<br>B-total leucocyte count [WBC]<br>B-neutrophils[a] [NEUTLE]<br>B-eosinophils[a] [EOSLE]<br>B-basophils[a] [BASOLE]<br>B-lymphocytes[a] [LYMLE]<br>B-monocytes[a] [MONOLE]<br>B-thrombocyte count [PLAT]<br>B-haematocrit [HCT]<br>P-prothrombin time [PT]	S-total bilirubin [BILI]<br>S-conjugated bilirubin [BILDIR]<br>S-alkaline phosphatase [ALP]<br>S-alanine aminotransferase [ALT]<br>S-aspartate aminotransferase [AST]<br>S-gamma-glutamyl transferase [GGT]	U-protein (dipstick) [PROT]<br>U-glucose (dipstick) [GLUC]<br>U-blood (dipstick) [OCCBLD]
**Electrolytes**[b]	**Kidney**[b]	**Pregnancy**[d]
S-sodium [SODIUM]<br>S-potassium [K]<br>S-calcium (total) [CA]	S-creatinine [CREAT]<br>S-urea nitrogen [UREAN]	S-human chorionic gonadotropin [HCG]<br>Urine dipstick
**Endocrine and Metabolic**[b]	**Lipids**[b,c]	
S-albumin [ALB]<br>S-glucose[c] [GLUC]<br>B-HbA1c [HBA1C]<br>S-creatine phosphokinase [CK]	S-low density lipoprotein [LDL]<br>S-high density lipoprotein [HDL]<br>S-triglycerides [TRIG]<br>S-cholesterol (total) [CHOL]	
B = blood; P = plasma; S = serum; U = urine; [ ] = CDISC term		

a Count and % of total leucocytes
b Clinical chemistry
c Fasting, when possible
d Only for women of childbearing potential. Pregnancy test at the Baseline Visit and the Safety Follow-up Visit is to be conducted using serum β-HCG. At all other visits, urine pregnancy testing will be performed and in case of a positive finding, further confirmatory testing will be performed via serum β-HCG.
e If urine dipstick is positive, a urine microscopic panel will be conducted.

Blood samples for the clinical safety laboratory tests will be collected as outlined in Panel 4.

The blood sampling and handling procedures are described in the study-specific *Laboratory Specification Manual.*

The blood samples will be analysed at the at the central laboratory. In case of exceptional circumstances or emergency, the investigator may need to request additional laboratory testing at a local laboratory. The investigator will record only out-of-range clinical safety laboratory test values considered clinically significant, which must be recorded as an adverse event on an *Adverse Event Form*.

Urine samples will be collected and analysed at the central laboratory.

The investigator must review (initial and date) the results of the clinical safety laboratory tests as soon as possible after receipt of those results. Out-of-range values must be interpreted by the investigator as "not clinically significant" or "clinically significant" with a comment concerning the planned follow-up. Tests for clinically significant out-of-range values must be

repeated, or an appropriate clinical follow-up must be arranged by the investigator and documented on the laboratory report, until the value has stabilized or until the value has returned to a clinically acceptable value (regardless of relationship to the IMP). A patient with a value that is out-of-range at the Safety Follow-up Visit and considered clinically significant must be followed in accordance with usual clinical practice or until the value normalizes or stabilizes or a diagnosis or reasonable explanation has been established. Any out-of-range values followed after the last protocol-specified contact with the patient will be documented in the patient's medical records.

Any out-of-range clinical safety laboratory test value considered clinically significant by the investigator must be recorded as an adverse event on an *Adverse Event Form*.

Urine pregnancy testing will be performed and analysed at site. All other urine samples will be collected and analysed at the central laboratory.

The central laboratory will be notified by the sponsor when the biological samples may be destroyed.

9.5.3 Vital Signs

The investigator may appoint a designee (for example, nurse or paramedic) to measure vital signs, provided this is permitted according to local regulations and provided the investigator has trained the designee how to measure vital signs. The investigator must take responsibility for reviewing the findings.

Pulse rate and blood pressure will be measured using a standard meter. Pulse rate and blood pressure will be measured in the following order: supine, sitting, and standing after the patient has rested in each position for at least 3 minutes.

Vital signs including body temperature must be assessed prior to blood sampling

Any out-of-range vital sign considered clinically significant by the investigator must be recorded as an adverse event on an *Adverse Event Form*.

9.5.4 Weight

The patients will be weighed wearing light clothing and no shoes. A similar amount of clothing must be worn on each occasion.

Any weight change considered clinically significant by the investigator must be recorded as an adverse event on an *Adverse Event Form*.

9.5.5 Electrocardiograms (ECGs)

A standard 12-lead ECG will be recorded using digital ECG recording equipment provided to the investigator or, upon agreement, to an external cardiology centre. The ECGs will be

transferred digitally to a central ECG laboratory for evaluation. The investigator will be provided with the results and a cardiological interpretation of the ECG from the central ECG laboratory.

The results from the central ECG laboratory will include the RR, PR, QRS, QT, and QT_c intervals.

The investigator has the final decision on the interpretation of the ECG results. Any abnormal ECG result or out-of-range ECG parameter value considered clinically significant by the investigator must be recorded as an adverse event on an *Adverse Event Form*.

9.5.6 Physical and Neurological Examinations

The investigator may appoint a designee to be primarily responsible for performing the physical examinations, provided this is permitted according to local regulations. The investigator must take responsibility for reviewing the findings. Whenever possible, the same individual should perform all the physical examinations.

Physical and neurological examinations for all clinic visits are to be conducted at the discretion of the investigator. If the examinations are conducted at the IMP Visits then these must be performed prior to the infusion.

The physical examination must, at a minimum, include an examination of appearance, extremities, skin, head, neck, eyes, ears, nose, throat, lungs, chest, heart, abdomen, genito-urinary system, and musculoskeletal system and must be performed by a physician or physician assistant.

If “genitourinary system evaluation” is not clinically indicated based on patient medical history or symptoms, it is accepted by Lundbeck not to conduct such evaluation. Examination of renal regions are to be included as part of abdominal examination.

The neurological examination must be performed by a physician.

Any abnormal finding or out-of-range value considered clinically significant by the investigator must be recorded as an adverse event on an *Adverse Event Form*.

9.5.7 Columbia-Suicide Severity Rating Scale

The C-SSRS is a semi-structured interview developed to systematically assess suicidal ideation and behaviour of patients participating in a clinical study.[47] The C-SSRS has 5 questions addressing suicidal ideation, 5 sub-questions assessing the intensity of ideation, and 4 questions addressing suicidal behaviour. For this study, the “Since last visit” version will be used for all visits. The Baseline/Screening version (lifetime and 1-year assessment) will be done in the Lead-in study. It takes approximately 5 minutes to administer and rate the C-SSRS.

The C-SSRS must be administered in the local language.

The C-SSRS should only be administered by a rater who has adequate experience with clinical studies in CNS indications. The rater should be a clinician, such as a neurologist, geriatrician, psychiatrist, or (neuro-) psychologist involved in clinical practice or regularly evaluating patients. Any exceptions must be discussed and approved by Lundbeck and/or its designee. For each individual patient, the same certified rater should preferably rate the patient throughout the study. In case of unforeseen circumstances, certified back-up raters should be available throughout the study.

Rater training and certification will be conducted by the CRO as agreed with the sponsor. Raters will complete their designated training curriculum based on their initial qualification status and assigned role. Only raters who qualify on study specific Rater Certification Programme will be authorized to administer the C-SSRS in the study. Documentation of training and certification will be provided to raters for archiving in the investigator trial master file (TMF). No patient must be rated before the documentation has been archived. New raters joining the study must be trained and certified by using the same certification process. Detailed instructions on how to administer the C-SSRS will be provided to the site in a *C-SSRS Guideline*.

If the patient answers "yes" to suicidal ideation questions 4 or 5, or answers "yes" to suicidal behaviour on the C-SSRS at the Safety Follow-up Visit, then the patient must be followed in accordance with usual clinical practice. The follow-up will be documented in the patient's medical records.

9.5.8 Anti-Drug Antibody (ADA) including Neutralizing Antibody (NAb) Assessments

Blood samples for the ADA including NAb assessments will be collected as outlined in Panel 2.

Patients who test positive for ADA at the Safety Follow-up Visit will be asked to provide up to two additional blood samples for immunogenicity testing at 12-week (±1 week) intervals for up to 24 weeks. The results will be reported separately from the Clinical Study Report (CSR).

The blood sampling and handling procedures are described in the study-specific *Laboratory Specification Manual*.

The blood samples will be analysed under the responsibility of Department of Bioanalysis - Biologics, H. Lundbeck A/S, according to a protocol approved by Lundbeck.

9.6 Order of Assessments

No study related activities must be conducted until after the applicable *Informed Consent Form* is signed.

PROs:

- PROs scheduled in alignment with a clinic visit (see Panel 2) can be completed at the clinic or in the remote setting within 3 days prior to the scheduled clinic visit date.
- PROs scheduled in alignment with a phone contact (see Panel 2) must be completed in the remote setting and can be completed on the day or within 3 days prior to the scheduled phone contact date
- HIT-6 should preferably be the first PRO completed, followed by the PGIC, MBS, MSQ v2.1, EQ-5D-5L, HCRU, WPAI:M.
- It is preferable that the same order of assessments is used per patient and if the scheduled time of the day for the assessments is as consistent as possible across all the study visits.

At IMP Visits, **prior** to infusion:

- Patients must complete recording of headaches which ended prior to infusion (i.e., for headaches which are ongoing or not yet recorded in the eDiary).
- Patients must complete the PROs (if completed at the clinic). PROs can be completed at the patient's convenience before or after the pre-infusion blood and urine sampling.
- The following assessments must be conducted: vital signs including body temperature, concomitant medications, AEs, physical and neurological examination (if done at the discretion of the investigator), ECG, blood sampling (for clinical safety laboratory tests, ADA and PK analysis) and urine sampling (for clinical safety laboratory and pregnancy tests) and C-SSRS administration. Vital signs must be assessed prior to blood sampling.
- See section 8.4 for procedures preceding IMP administration.

At IMP Visits, **during** infusion:

- IRRs must be checked as part of the overall AE collection. IRRs must be assessed after the AE collection.

At IMP Visits **after** end-of-IMP-infusion and before the patient is discharged from the site:

- The following assessments must be conducted: vital signs including body temperature, IRRs and AEs. IRRs must be assessed after the AE collection.
- A blood sample for eptinezumab quantification must be taken within 1 hour after end-of-infusion. Vital signs must be assessed prior to blood sampling.
- See section 8.4 for procedures following IMP administration.

9.7 Total Volume of Blood Drawn

The total volume of blood drawn from each patient will be approximately 119.5 mL during the study spread out over 68 weeks.

Additional blood samples may be required if the original blood samples are not viable or if re-testing is required.

9.8 Treatment Compliance

Responsible study personnel will administer the infusions of IMP. Treatment compliance verification should be documented in the patient's source documents and study specific IMP documents and verified by a CRA during monitoring.

Anyone administering the IMP to the patient must be listed in the delegation log.

The information from the *IMP Administration Form* must be entered in the eCRF.

10 Adverse Events

10.1 Definitions

10.1.1 Adverse Event Definitions[48]

Adverse event – is any untoward medical occurrence in a patient or clinical study patient administered a medicinal product and which does not necessarily have a causal relationship with this treatment.

An adverse event can therefore be any unfavourable and unintended sign (including clinically significant out-of-range values from relevant tests, such as clinical safety laboratory tests, vital signs, ECGs), symptom, or disease temporally associated with the use of a medicinal product, regardless of whether it is considered related to the medicinal product.

A new sign/symptom or worsening of a pre-existing or chronic condition is considered an adverse event and must be reported as such. Medical conditions, which existed prior to the time of informed consent into the clinical study are part of the patient's medical history and are not considered an adverse event. Unchanged, chronic, non-worsening or pre-existing conditions from the time of informed consent are not adverse events and should not be recorded on the AE CRF. A pre-existing or chronic condition that worsens after signing the informed consent is considered an adverse event.

It is Lundbeck policy to collect and record all adverse events, including pre-treatment adverse events, that is, those that start after the patient has signed the *Informed Consent Form* and prior to the first dose of IMP.

Serious adverse event (SAE) – is any adverse event that:
- results in death
- is life-threatening (this refers to an event in which the patient was at risk of death at the time of the event; it does not refer to an event that hypothetically might have caused death had it been more severe)
- requires inpatient hospitalization or prolongation of existing hospitalization
- results in persistent or significant disability/incapacity

- is a congenital anomaly/birth defect
- is medically important (this refers to an event that may not be immediately life-threatening or result in death or hospitalization, but may jeopardize the patient or may require intervention to prevent any of the SAEs defined above)

Examples of medically important events are intensive treatment for allergic bronchospasm; blood dyscrasia or convulsions that do not result in hospitalization; or development of drug dependency or drug abuse.

Planned hospitalizations or surgical interventions for a condition that existed before the patient signed the *Informed Consent Form* and that did not change in intensity are not adverse events. Emergency room visits that do not result in admission to the hospital are not necessarily SAEs; however, they must be evaluated to determine whether they meet any of the SAE definitions (for example, life-threatening or other serious [medically important] event).

Non-serious adverse event – is any adverse event that does not meet the definition of an SAE.

If there is any doubt as to whether an adverse event meets the definition of an SAE, a conservative viewpoint must be taken, and the adverse event must be reported as an SAE.

Suspected unexpected serious adverse reaction (SUSAR) – is any adverse event that is assessed as serious, unexpected (its nature or intensity is not consistent with the current version of the *Investigator's Brochure*[24] and related to a medicinal product by either the investigator or Lundbeck.

Overdose – is a dose taken by a patient that exceeds the dose prescribed to that patient. Any overdose (and associated symptoms) must, at a minimum, be recorded as a non-serious adverse event.

10.1.2 Adverse Event Assessment Definitions

Assessment of Intensity

The investigator must assess the *intensity* of the adverse event using the following definitions, and record it on the *Adverse Event Form*:

- *Mild* – the adverse event causes minimal discomfort and does not interfere in a significant manner with the patient's normal activities.
- *Moderate* – the adverse event is sufficiently uncomfortable to produce some impairment of the patient's normal activities.
- *Severe* – the adverse event is incapacitating, preventing the patient from participating in the patient's normal activities.

Assessment of Causal Relationship

The investigator must assess the *causal relationship* between the adverse event and the IMP using the following definitions, and record it on the *Adverse Event Form* and the *Serious Adverse Event Form* (if applicable):

- *Probable* – the adverse event has a strong temporal relationship to the IMP or recurs on rechallenge, and another aetiology is unlikely or significantly less likely.
- *Possible* – the adverse event has a suggestive temporal relationship to the IMP, and an alternative aetiology is equally or less likely.
- *Not related* – the adverse event has no temporal relationship to the IMP or is due to underlying/concurrent disorder or effect of another drug (that is, there is no causal relationship between the IMP and the adverse event).

An adverse event is considered causally related to the use of the IMP when the causality assessment is *probable* or *possible*.

Assessment of Outcome

The investigator must assess the *outcome* of the adverse event using the following definitions, and record it on the *Adverse Event Form* and the *Serious Adverse Event Form* (if applicable):

- *Recovered* – the patient has recovered completely, and no symptoms remain.
- *Recovering* – the patient's condition is improving, but symptoms still remain.
- *Recovered with sequelae* – the patient has recovered, but some symptoms remain (for example, the patient had a stroke and is functioning normally, but has some motor impairment).
- *Not recovered* – the patient's condition has not improved and the symptoms are unchanged (for example, an atrial fibrillation has become chronic).
- *Death*

10.2 Management of Reactions to Study Drug

There are no specific antidotes to an infusion of eptinezumab.

A medical emergency should be treated appropriately by the investigator using proper standard of care, according to their typical clinical practice and local guidelines for that emergency condition.

Should a medical condition arise that the investigator believes is related to the study drug, clinical judgement should be used to provide appropriate response including the consideration of dose reduction or of discontinuation of study drug. If a patient experiences an anaphylactic reaction or another severe and/or serious hypersensitivity reaction during the IMP infusion, as assessed by the investigator, the infusion must be discontinued immediately (see section 5.3) and appropriate therapy instituted. Any events believed to be allergic reactions should be discussed with the medical monitor.

Per investigator judgement, it can be considered, to collect additional immune response tests in accordance with local clinical practice, such as histamine, tryptase, immunoglobulin E, and complement components C3 and C4.

10.3 Pregnancy

Although not necessarily considered an adverse event, a pregnancy in a patient in the study must be recorded on an *Adverse Event Form*, as well as on a *Pregnancy Form* (paper), even if no adverse event associated with the pregnancy has occurred. Pregnancies must be reported to Lundbeck using the same expedited reporting timelines as those for SAEs.

An uncomplicated pregnancy should not be reported as an SAE; hospitalization for a normal birth should not be reported as an SAE. If, however, the pregnancy is associated with an SAE, the appropriate serious criterion must be indicated on the *Serious Adverse Event Form*. Examples of pregnancies to be reported as SAEs (medically important) are spontaneous abortions, stillbirths, and malformations.

The investigator must follow up on the *outcome* of the pregnancy and report it on a *Pregnancy Form* (paper). The follow-up must include information on the neonate at least up until the age of 1 month.

10.4 Recording Adverse Events

Adverse events must be recorded on an *Adverse Event Form*. The investigator must provide information on the adverse event, preferably with a diagnosis, or at least with signs and symptoms; start and stop dates (and start and stop time if the adverse event lasts less than 24 hours or occurs on the day of IMP administration); intensity; causal relationship to the IMP; action taken; and outcome. If the adverse event is not related to the IMP, an alternative aetiology must be recorded, if available. If the adverse event is an overdose, the nature of the overdose must be stated (for example, medication error, accidental overdose, or intentional overdose). If the intensity changes during the course of the adverse event, this must be recorded on the *AE Intensity Log*.

If the adverse event is *serious*, this must be indicated on the *Adverse Event Form*. Furthermore, the investigator must fill out a *Serious Adverse Event Form* and report the SAE to Lundbeck immediately (within 24 hours) after becoming aware of it (see section 10.5).

If individual adverse events are later linked to a specific diagnosis, the diagnosis should be reported and linked to the previously reported adverse events.

10.5 Reporting Serious Adverse Events (SAEs)

The investigator must report SAEs to Lundbeck immediately (within 24 hours) after becoming aware of them by completing a *Serious Adverse Event Form* in RAVE.

The initial *Serious Adverse Event Form* must contain as much information as possible and, if more information about the patient's condition becomes available, the *Serious Adverse Event Form* must be updated with the additional information.

If the investigator cannot report the SAE in Rave®, then he or she must complete and sign the *Serious Adverse Event Fallback Form* and send it to:

Fax: +45 36 30 99 67
email: ICSRquery@lundbeck.com

When RAVE is available again, the site must enter the SAE in RAVE for consistency.

Lundbeck will assume responsibility for reporting SAEs to the authorities in accordance with local requirements.

It is the investigator's responsibility to be familiar with local requirements regarding reporting SAEs to the EC and to act accordingly.

Lundbeck will assess the expectedness of SAEs and inform the investigator(s) about SUSARs in the blinded SUSAR listings.

10.6 Treatment and Follow-up of Adverse Events

Patients with adverse events must be treated in accordance with usual clinical practice at the discretion of the investigator.

Non-serious adverse events must be followed up until resolution or the Safety Follow-up Visit, whichever comes first. At the Safety Follow-up Visit, information on new AEs, if any, and stop dates for previously reported adverse events must be recorded.

The investigator must follow up on all SAEs until the patient has recovered, stabilized, or recovered with sequelae, and report to Lundbeck all relevant new information using the same procedures and timelines as those for the initial *Serious Adverse Event Form*.

SAEs that are spontaneously reported by a patient to the investigator after the Safety Follow-up Visit must be handled in the same manner as SAEs that occur during the study. These SAEs will be recorded in the Lundbeck safety database.

Patients with a clinically significant out-of-range clinical safety laboratory test value at the Safety Follow-up Visits must be followed in accordance with usual clinical practice. If the clinically significant out-of-range clinical safety laboratory test value has not normalized or stabilized or a diagnosis or a reasonable explanation has not been established by the Safety Follow-up Visit, the investigator must decide whether further follow-up visits are required (this may include an additional medical examination and/or additional blood sampling). If further follow-up visits are made, these must be documented in the patient's medical records and not in the eCRF.

Patients who withdraw due to an elevated AST or ALT value (see section 5.3) must be followed until the values normalize or stabilize or a diagnosis or a reasonable explanation has been established. Additional medical examinations (for example, ultrasound scanning and/or sampling for serology, conjugated bilirubin, prothrombin time) should be considered. A gastroenterology or hepatology consultation should also be considered.

10.7 Study Monitoring Committee(s)

10.7.1 Data Monitoring Committee (DMC)

The DMC consists of consist of medical doctors with speciality relevant to the fields of neurology and cardiology, as well as a biostatistician. The DMC will monitor safety data on an ongoing basis in addition to cumulative safety data. The DMC will be informed to what extent the data and analyses provided to them have been quality controlled. Members of the DMC will not be involved in other study-related tasks. The DMC procedures are described in the Data Monitoring Committee Charter.

11 Data Handling and Record Keeping

11.1 Data Collection

11.1.1 Electronic Case Report Forms (eCRFs)

eCRFs will be used to collect all the data related to the study, except the external data described in section 11.1.3.

The eCRFs use third party software (Rave®) to capture data via an online system on a computer. When the investigator enters data in the eCRF (ideally during the visit or as soon as possible [<3 days] thereafter), the data will be recorded electronically in a central database over encrypted lines, and all entries and modifications to the data will be logged in an audit trail. Access to the system will only be granted after appropriate and documented training. Written instructions for using the system will be provided along with the training.

Electronic signatures will be used where signatures are required on pages and/or visits. Automated data entry checks will be implemented where appropriate; other data will be reviewed and evaluated for accuracy by the sponsor and/or representatives from the CRO. All entries, corrections, and changes must be made by the investigator or a delegate.

11.1.2 Patient Binders

11.1.2.1 Use of Patient Binders

A *Patient Binder* will be provided for each patient. The *Patient Binder* contains different types of source documents, organized by visit and type. A ballpoint pen with waterproof ink must be used to enter information in the *Patient Binder*.

11.1.2.2 Serious Adverse Event Fallback Forms

Serious Adverse Event Fallback Forms must be used when the eCRF cannot be accessed.

11.1.3 External Data

All electronic data will be transferred using a secure method accepted by Lundbeck.

The following electronic data will be transferred by the vendor and kept in a secure designated storage area outside the eCRF:
- eDiary data
- ePRO data
- eCSSRS data
- ECG results
- Clinical Safety Laboratory data
- Urine sampling data for clinical safety laboratory
- Blood sampling for ADA including Nab data
- Blood sampling for eptinezumab quantification data

The entries in the eDiary will be used to calculate the endpoints using a pre-specified algorithm, completely based on the reported data. In case of missing entries, pre-specified rules and algorithms will also be used. The rules and algorithms will be based on the ICHD criteria for migraine (see section 9.2.1) and fully specified in the SAP.

11.2 Retention of Study Documents at the Site

11.2.1 eCRF Data

If a site closes before the study has been completed, the investigator will continue to have read-only access to the eCRF until the study has been completed. After the study has been completed, all user access to the eCRF will be revoked. Renewed access to the eCRF will be given if corrections or updates to the database are required.

At the end of the study, the site will be provided with all data related to the site (including eCRF data, queries, and the audit trail) using a secure electronic medium; the secure storage of these data at the site is the responsibility of the investigator. When confirmation of receipt

of the data has been received from all sites, all user access to the eCRF will be revoked. If, for some reason, the data are not readable for the full retention period (25 years or in accordance with national requirements, whichever is longer), the investigator may request that the data be re-sent.

11.2.2 Other Study Documents

The investigator must keep the investigator's set of documents in the investigator TMF for at least 25 years after the *Clinical Study Report* has been approved or in accordance with national requirements, whichever is longer. Lundbeck will remind the investigator in writing of this obligation when the *Clinical Study Report Synopsis* is distributed to the site.

If off-site storage is used, a study-specific binder will remain at the site after the other study-specific documents have been shipped for off-site storage. This binder is considered part of the investigator TMF and must be kept in a secure place by the site for the required period of time. The binder must contain, at a minimum, the following documents: a copy of the *Investigator TMF Index*, a certified copy of the *Patient Identification Code List*, and a *Retrieval Form*.

When the required storage period has expired, the documents may be destroyed in accordance with regulations.

12 Monitoring Procedures

Prior to allowing patients to participate in the study, the investigator must sign a source data agreement that identifies the source documents (original documents, data, and records) at the site. The document will also list which data may be recorded directly on the eCRFs.

During the study, the CRA will visit the site to ensure that the protocol is being adhered to and that all issues are being recorded, to perform source data verification, and to monitor IMP accountability. The visit intervals will depend on the outcome of the remote monitoring of the eCRFs, the site's recruitment rate, and the compliance of the site to the protocol and *Good Clinical Practice*. In addition, the CRA will be available for discussions by telephone.

Source data verification requires that the CRA be given direct access to all the source documents. Direct access includes permission to examine and verify any records that are important for the evaluation of the study.

13 Audits and Inspections

Authorized personnel from Medical, Regulatory and Clinical Quality Assurance, H. Lundbeck A/S, and quality assurance personnel from business partners may audit the study at any time to assess compliance with the protocol and the principles of *Good Clinical Practice* and all other relevant regulations.

The investigator must be aware that representatives from regulatory authorities may also wish to inspect source data, such as medical records. The investigator must notify Lundbeck, without delay, of an announced inspection by a regulatory authority.

During audits and inspections, the investigator must permit direct access to all the source documents, including medical records and other documents pertinent to the study.

During audits and inspections, the auditors and inspectors may request relevant parts of medical records. No personal identification apart from the screening or randomization numbers will appear on these copies.

Patient data will not be disclosed to unauthorized third parties, and patient confidentiality will be respected at all times.

14 Protocol Compliance

Lundbeck has a "no-waiver" policy, which means that permission will not be given to deviate from the protocol.

If a deviation occurs, the investigator must inform the CRA and they must review, discuss, and document the implications of the deviation.

15 Study Termination

Lundbeck or a pertinent regulatory authority may terminate the study or part of the study at any time. The reasons for such action may include, but are not limited to:

- safety concerns
- proven lack of efficacy of the IMP in other studies

If the study is terminated or suspended, the investigator must promptly inform the patients and ensure appropriate therapy and follow-up. Furthermore, the investigator and/or sponsor must promptly inform the IRB and provide a detailed written explanation. The pertinent regulatory authorities must be informed in accordance with national regulations.

If the risk/benefit evaluation changes after the study is terminated, the new evaluation must be provided to the IRB if it will have an impact on the planned follow-up of the patients who participated in the study. If so, the actions needed to protect the patients must be described.

16 Statistical Methodology

16.1 Responsibilities

Biostatistics, H. Lundbeck A/S will perform the statistical analyses described below.

16.2 Analysis Sets

The following analysis set(s) will be used for the analyses:

- *all-patients-enrolled set* (APES) – all patients who have completed the Lead-in Study and are enrolled into the Open-label Study
- *all-patients-treated set* (APTS) – all patients in the APES who received IMP in the Open-Label Study
- *full-analysis set* (FAS) – all patients in the APTS who had a valid baseline MMD, based on the eDiary data from the last 28 days of the Lead-in Study, and at least one post-baseline observation of MMD

The patients and data will be classified into the analysis sets according to these definitions at a *Classification Meeting* held after the study database has been released.

16.3 Descriptive Statistics

In general, summary statistics (n, arithmetic mean, standard deviation, median, lower and upper quartiles, minimum and maximum values) will be presented for continuous variables and counts and, if relevant, percentages will be presented for categorical variables.

16.4 Patient Disposition

Patient disposition will be summarized and include the number of patients in the APTS who completed or withdrew from the study, as well as the number of patients in each analysis set (APES, APTS and FAS).

The number of patients who withdrew from study will be summarized by primary reason for withdrawal and all reasons for withdrawal, for all patients and by previous treatment that the patient was exposed to in the Lead-in Study.

The summary of patient disposition will be presented for all patients and by previous treatment.

16.5 Demographics and Baseline Characteristics

Demographics (sex, age, and race) and baseline weight will be summarized for the APTS, for all patients and by previous treatment. Baseline efficacy variables will be summarised for the FAS, for all patients and by previous treatment.

16.6 Recent and Concomitant Medication

Recent and concomitant medication will be summarized by anatomical therapeutic chemical (ATC) code and generic drug name.

16.7 Exposure

All patients in the APTS are expected to receive five single infusions of the IMP and the infusion time duration will be summarised for all patients and by previous treatment. Included patients whose infusion took more than 45 mins and patients who had their infusion interrupted will be listed with infusion start date/time and end date/time, infusion related reactions, and reasons if any.

For each scheduled infusion, the number of patients received the infusion and the volume of infusion will also be summarized.

16.8 Efficacy Analyses

16.8.1 General Efficacy Analysis Methodology

The analysis of endpoints will be presented with 95% CIs, unless otherwise specified. Baseline visit of the Open-label Study will be used for all efficacy analyses.

16.8.2 Analysis of the Secondary Endpoints

For the number of MMDs, details on derivation and imputations of days with missing or incomplete eDiary data will be described in the Statistical analysis Plan (SAP).

Absolute value and change from baseline in the number of MMDs, 50% response rates, HIT-6 score, EQ-5D-5L VAS score and MBS score will be summarized descriptively for all patients and by previous treatment. Absolute value of PGIC will be summarized for all patients and by previous treatment.

Change from baseline in the number of MMDs, HIT-6 score, EQ-5D-5L VAS score and MBS score will be analysed using mixed model repeated measures (MMRM) including visit and previous treatment as fixed factors, baseline as covariate, and previous treatment-by-visit interaction and baseline-by-visit interaction. For PGIC, a similar model will be fitted, without baseline and baseline-by-visit interaction.

16.8.3 Analysis of the Exploratory Endpoints

Plasma concentration of Eptinezumab will be summarized for all patients and by previous treatment.

Absolute value and change from baseline in MSQ subscores, HCRU and WPAI:M subscores will be summarized descriptively for all patients and by previous treatment. Change from baseline in MSQ v2.1 subscores and WPAI:M subscores will be analysed using MMRM.

16.9 Safety Analyses

16.9.1 General Safety Analysis Methodology

Safety endpoints will be descriptively summarised by previous treatment and overall. The summaries of changes from baseline will be presented relative to both the baseline in the Open-label Study and the baseline in the Lead-in Study.

16.9.2 Analysis of Adverse Events

Adverse events will be classified according to the time of onset of the adverse event:

- *treatment-emergent adverse event* (TEAE) – an adverse event that starts or increases in intensity during or after the administration of the first dose of IMP

A new condition or worsening of a pre-existing or chronic condition is considered an adverse event and must be reported as such. Unchanged, chronic, non-worsening or pre-existing conditions from the time of enrolment are not adverse events and should not be recorded on the AE CRF. A pre-existing or chronic condition that worsens after enrolment to the study is considered an adverse event.

Adverse events, sorted by system organ class (SOC) and preferred term, will be summarized for all patients and by previous treatment.

16.9.3 Analysis of Other Safety Endpoints

The clinical safety laboratory test values, vital signs, ECG parameter values_will be summarized by visit for all patients and by previous treatment. Potentially clinically significant (PCS) values will be flagged and summarized.

C-SSRS score will be summarized for all patients and by previous treatment.

The ADA-positive and Nabs-positive samples and their titer values will be summarised for all patients and by previous treatment.

16.10 Interim Analyses

No interim analysis is planned.

16.11 Sample Size and Power

No formal sample size calculations have been performed. The study is planned to enrol patients in Japan who have completed the Lead-in Study and fulfilled the entry criteria for the Open-label Study. It is expected to have patients complete the study.

16.12 Statistical Analysis Plan

A *Statistical Analysis Plan* describing the handling of data issues and the planned statistical analyses in more detail will be prepared by Biostatistics, H. Lundbeck A/S, before the Open-label Study or the Lead-in Study are unblinded.

17 Clinical Study Report and Publications

17.1 Data Ownership

The data collected in this study are the property of Lundbeck.

17.2 Clinical Study Report

Upon completion of the study, a *Clinical Study Report* will be prepared by Regulatory Medical Writing, H. Lundbeck A/S.

17.3 Summary of Clinical Study Results

Upon completion of the study and when the study results are available, the patient has the right to be informed by the investigator about the overall study results.

17.4 Publications

The results of this study will be submitted for publication at the discretion of Lundbeck.

The primary publication based on this study must be published before any secondary publications. Authors of the primary publication must fulfil the criteria defined by the International Committee of Medical Journal Editors (ICMJE).[49]

18 Indemnity and Insurance

In the event of study-related injuries or deaths, insurance for the patients and indemnity of the investigators and those of their employees, servants, or agents whose participation in this study has been documented are provided. Insurance and liability will be in accordance with applicable laws and *Good Clinical Practice*.

19 Finance

19.1 Site Agreements

The financial agreements with each site are addressed in one or more documents. Both parties must sign the agreements before each site is initiated.

19.2 Financial Disclosure

All the investigators, including sub-investigators, and raters participating in the study must complete a *Financial Disclosure Form*.

19.3 Equipment

Equipment owned or rented by Lundbeck that has been provided to the sites for use during the study must be returned at the end of the study.

References

1. International Headache Society (IHS), Headache Classification Committee. The International Classification of Headache Disorders, 3rd edition 2018 (ICHD-3 2018).
2. Lipton RB, Bigal ME, Diamond M, Freitag F, Reed ML, Stewart WF. Migraine prevalence, disease burden, and the need for preventive therapy. Neurology. 2007; 68: 343–349.
3. Steiner TJ, Stovner LJ, Vos T, Jensen R, Katsarava Z. Migraine is first cause of disability in under 50s: will health politicians now take notice? J Headache Pain 2018; 19: 17.
4. Leonardi M, Steiner TJ, Scher AT, Lipton RB. The global burden of migraine: measuring disability in headache disorders with WHO's classification of functioning, disability and health (ICF). J Headache Pain. 2005; 6(6): 429–440.
5. GBD 2016 Headache Collaborators. Global, regional, and national burden of migraine and tension-type headache, 1990–2016: a systematic analysis for the Global Burden of Disease Study 2016. Lancet Neurol. 2018; 17: 954–976.
6. Katsarava Z, Buse DC, Manack AN, Lipton RB. Defining the differences between episodic migraine and chronic migraine. Curr Pain Headache Rep 2012; 16: 86–92.
7. Headache Classification Committee of the International Headache Society. The International Classification of Headache Disorders, 3rd edition. Cephalalgia. 2018, 38(1): 1–211.
8. Silberstein SD. Preventive migraine treatment. Continuum. 2015; 21: 973–989.
9. Lipton RB, Silberstein SD. Episodic and chronic migraine headache: breaking down barriers to optimal treatment and prevention. Headache. 2015; 55 (suppl 2): 103–122.
10. Hepp Z, Dodick DW, Varon SF, Gillard P, Hansen RN, Devine EB. Adherence to oral migraine-preventive medications among patients with chronic migraine. Cephalalgia. 2015; 35: 478–488.
11. Hepp Z, Bloudek LM, Varon SF. Systematic review of migraine prophylaxis adherence and persistence. J Manag Care Pharm. 2014; 20: 22–33.
12. Blumenfeld AM, Bloudek LM, Becker WJ, Buse DC, Varon SF, Maglinte GA, et al. Patterns of use and reasons for discontinuation of prophylactic medications for episodic migraine and chronic migraine: results from the second international burden of migraine study (IBMS-II). Headache. 2013; 53: 644-655.
13. Berger A, Bloudek LM, Varon SF, Oster G. Adherence with migraine prophylaxis in clinical practice. Pain Pract. 2012; 12: 541–549.
14. American Headache Society. The American Headache Society Position Statement on Integrating New Migraine Treatments Into Clinical Practice. Headache. 2019; 59(1): 1-18.
15. Silberstein SD. Practice parameter: evidence-based guidelines for migraine headache (an evidence-based review): report of the Quality Standards Subcommittee of the American Academy of Neurology. Neurology. 2000; 55(6): 754-762.
16. Puledda F, Messina R, and Goadsby PJ. An update on migraine: current understanding and future directions. J Neurol. 2017; 264(9): 2031-2039.
17. Storer RJ, Akerman S, Goadsby PJ. Calcitonin gene-related peptide (CGRP) modulates nociceptive trigeminovascular transmission in the cat. Br J Pharmacol. 2004; 142(7): 1171–1181.

18. Jenkins DW, Langmead CJ, Parsons AA, Strijbos PJ. Regulation of calcitonin gene-related peptide release from rat trigeminal nucleus caudalis slices in vitro. Neurosci Lett. 2004; 366(3): 241–244.

19. Krishnaswamy R, Malik BH, Khan S, Gupta D, Islam M. Anti-CGRP monoclonal antibodies: breakthrough in migraine therapeutics. Progress in Neurology and Psychiatry. 2019; 23: 26-33.

20. Reuter U, Goadsby PJ, Lanteri-Minet M, Wen S, Hours-Zesiger P, Ferrari MD, et al. Efficacy and tolerability of erenumab in patients with episodic migraine in whom two-to-four previous preventive treatments were unsuccessful: a randomised, double-blind, placebo-controlled, phase 3b study. Lancet. 2018; 392: 2280–2287.

21. Ferrari MD, Diener HC, Ning X, Galic M, Cohen JM, Yang R, et al. Fremanezumab versus placebo for migraine prevention in patients with documented failure to up to four migraine preventive medication classes (FOCUS): a randomised, double-blind, placebo-controlled, phase 3b trial. Lancet. 2019; 394: 1030–1040.

22. Ashina, M, Saper J, Cady R, Schaeffler BA, Biondi DM, Hirman J, et al. Eptinezumab in episodic migraine: A randomized, double-blind, placebo-controlled study (PROMISE-I). Cephalagia. 2020; 0(0): 1-14.

23. Lipton RB, Goadsby PJ, Smith J, Schaeffler BA, Biondi DM, Hirman J, et al. Efficacy and safety of eptinezumab in patients with chronic migraine - PROMISE-2. 2020:10.1212/ WNL.0000000000009169.

24. H. Lundbeck A/S. Investigator's Brochure: eptinezumab. current version.

25. Davies B, Morris T. Physiological parameters in laboratory animals and humans. Pharm Res. 1993; 10(7): 1093-1095.

26. Ferri N, Bellosta S, Baldessin L, Boccia D, Racagni G, Corsini A. Pharmacokinetics interactions of monoclonal antibodies. Pharmacol Res. 2016; 111: 592-599.

27. Lipton RB, Bigal ME. The epidemiology of migraine. Am J Med. 2005; 118 Suppl 1: 3-10.

28. Gupta R, Levin E, Wu JJ, Koo J, Liao W. An update on drug-drug interactions with biologics for the treatment of moderate-to-severe psoriasis. J Dermatolog Treat. 2014; 25(1): 87-89.

29. Dodick DW, Goadsby PJ, Silberstein SD, Lipton RB, Olesen J, Ashina M, et al. Safety and efficacy of ALD403, an antibody to calcitonin gene-related peptide, for the prevention of frequent episodic migraine: a randomised, double-blind, placebo-controlled, exploratory phase 2 trial. Lancet Neurol. 2014; 13(11): 1100-1107.

30. Dodick DW, Lipton RB, Silberstein S, Goadsby PJ, Biondi D, Hirman J, et al. Eptinezumab for prevention of chronic migraine: A randomized phase 2b clinical trial. Cephalalgia. 2019; 39(9): 1075–1085.

31. Spierings E, Biondi D, Hirman J, Horblyuk R, Cady R. Reduced impact of headaches after migraine preventive treatment with eptinezumab in patients with chronic migraine: Results from the PREVAIL open-label safety study. Presented at: 2019 American Headache Society Annual Meeting. July 11-14, 2019; Philadelphia, PA. Poster 16.

32. Kudrow D, Berman G, Kassel E, et al. Eptinezumab treatment for migraine prevention reduces migraine disability in patients with chronic migraine: An analysis from the PREVAIL open-label safety study. Presented at: 2019 American Headache Society Annual Meeting. July 11-14, 2019; Philadelphia, PA. Poster 17.

33. ClinicalTrials.gov Identifier: NCT04152083.

34. Wang W, Wang EQ, Balthasar JP. Monoclonal antibody pharmacokinetics and pharmacodynamics. Clin Pharmacol Ther. 2008; 84(5): 548-558.

35. Li X, Zhou J, Tan G, Wang Y, Ran L, Chen L. Diagnosis and treatment status of migraine: a clinic-based study in China. J Neurol Sci. 2012; 315(1-2): 89-92.

36. Ueda K, Ye W, Lombard L, Kuga A, Kim Y, Cotton S, et al. Real-world treatment patterns and patient-reported outcomes in episodic and chronic migraine in Japan: analysis of data from the Adelphi migraine disease specific programme. J Headache Pain. 2019 Jun 7; 20(1): 68.

37. Takeshima T, Wan Q, Zhang Y, Komori M, Stretton S, Rajan N, et al. Prevalence, burden, and clinical management of migraine in China, Japan, and South Korea: a comprehensive review of the literature. The Journal of Headache and Pain. 2019; 20: 111.

38. James SL, Abate D, Abate KH, et al. Global, regional, and national incidence, prevalence, and years lived with disability for 354 diseases and injuries for 195 countries and territories, 1990–2017: a systematic analysis for the Global Burden of Disease Study 2017. The Lancet. 2018; 392: 1789-1858.

39. Luo N, Qi W, Tong W, Tan F, Zhang Q, He J, et al. Prevalence and burden of headache disorders in two neighboring provinces of China. J Clin Neurosci. 2014; 21: 1750-1754.

40. World Medical Association (WMA). Declaration of Helsinki: Ethical principles for medical research involving human subjects. [Internet] wma.net/en/30publications/10policies/b3/index.html.

41. ICH. ICH Harmonised Guideline E6(R2): Integrated addendum to ICH E6(R1): Guideline for Good Clinical Practice. November 2016.

42. European Parliament and Council of the European Union. Regulation (EU) 016/679: Protection of natural persons with regard to the processing of personal data and on the free movement of such data, and repealing Directive 95/46/EC (General Data Protection Regulation). 27 April 2016. Official Journal of the European Union L119, 4 May 2016.

43. Kosinski M, Bayliss MS, Bjorner JB, Ware JE, Garber WH, Cady R, et al. A six-item short-form survey for measuring headache impact: the HIT-6. Qual Life Res. 2003; 12(8): 963–974.

44. Jhingran P, Osterhaus JT, Miller DW, Lee JT, Kirchdoerfer L. Development and validation of the Migraine-Specific Quality of Life Questionnaire. Headache. 1998; 38(4): 295–302.

45. Herdman M, Gudex C, Lloyd A, Janssen M, Kind P, Parkin D, et al. Development and preliminary testing of the new five-level version of EQ-5D (EQ-5D-5L). Quality of Life Research. 2011; 20: 1727–1736.

46. Reilly MC, Zbrozek AS, Dukes, EM. The validity and reproducibility of a work productivity and activity impairment instrument. Pharmacoeconomics. 1993; 4(5): 353–365.

47. Posner K, Brown GK, Stanley B, Brent DA, Yershova KV, Oquendo MA, et al. The Columbia-Suicide Severity Rating Scale: initial validity and internal consistency findings from three multisite studies with adolescents and adults. Am J Psychiatry. 2011; 168: 1266–1277.

48. ICH. ICH Harmonised Tripartite Guideline E2A: Clinical safety data management: definitions and standards for expedited reporting. October 1994.

49. International Committee of Medical Journal Editors (ICMJE). Recommendations for the conduct, reporting, editing, and publication of scholarly work in medical journals. [Internet] icmje.org/icmje-recommendations.pdf. December 2017.

Appendix I

Clinical Study Protocol

Authentication and Authorization

Clinical Study Protocol
Authentication and Authorization

Study title:	Interventional, open-label, flexible-dose, long-term extension study to evaluate safety of eptinezumab as preventive treatment in patients with migraine in Japan
Study No.:	19140B
Edition No.:	2.0
Date of edition:	10 November 2021

This document has been signed electronically. The signatories are listed below.

Authentication

I hereby confirm that I am of the opinion that the ethical and scientific basis of this study is sound.

International study manager:	CCI
Clinical research scientist:	CCI
Head of Biostatistics:	CCI
Head of Medical Safety:	CCI

Authorization

I hereby confirm that I am of the opinion that the ethical and scientific basis of this study is sound.

Head of Therapeutic Area: Bjørn Sperling

Appendix II
Recent and Concomitant Medication
Disallowed or Allowed with Restrictions

Recent and Concomitant Medication: Disallowed or Allowed with Restrictions

In the table below, recent and concomitant medications that are disallowed or allowed with restrictions with respect to their use prior to or during the study are listed.

Drug Class	Details
Any investigational drug	• Do not use during the study.
Anticonvulsants	• See restrictions in use under *anti-migraine agents*. Other medication in the same class is allowed if prescribed for non-migraine indications.
Antihypertensives	• See restrictions in use under *anti-migraine agents*. Other medication in the same class is allowed if prescribed for non-migraine indications.
Anti-impotence agents	• Allowed if the dose has been stable during the Lead-in Study and expected to be maintained until the Completion Visit (Week 60).
Antimigraine agents	<u>Allowed with restrictions:</u><br>• Preventive treatment of migraine (prescription or over-the-counter medication recommended by a healthcare professional) is allowed provided the dose and the regimen have been stable during the Lead-in Study and expected to be maintained until the Completion Visit (Week 60).<br><u>Disallowed:</u><br>• Do not use oral anti-CGRPs for acute treatment until the Completion Visit (Week 60).<br>• Do not use eptinezumab or other monoclonal antibody targeting the CGRP pathway during the study.<br>• Do not use botulinum toxin for migraine or any other medical/cosmetic reason in the head and/or neck region until the Completion Visit (Week 60).<br>• Do not use monoamine oxidase inhibitors, ketamine, methysergide, methylergonovine, or nimesulide until the Completion Visit (Week 60).<br>• Do not use injectable therapy (trigger point injections, extracranial nerve blocks, or facet joint injections) < until the Completion Visit (Week 60).
Hormones	• Hormonal therapy (e.g, contraceptives, hormone replacement therapy) is allowed provided the dose and regimen have been stable during the Lead-in Study and expected to be maintained for ≥6 months after last dose of IMP.
Opioid analgesics	• Prescription opiates (including single-ingredient or combination medications containing opiates, opioids, tramadol, or tapentadol) are allowed provided a stable dose and regimen have been maintained during the Lead-in Study and expected to be maintained until the Completion Visit (Week 60). These agents may be prescribed when considered medically indicated by the investigator during the study and a stable dose and regimen are expected to be maintained.

Drug Class	Details
Other interventions and devices	Allowed with restrictions:<br>• Non-pharmacological interventions and therapies for the treatment of migraine (e.g., behavioural therapy and acupuncture etc) are allowed provided their use has been stable during the Lead-in Study and expected to be maintained until the Completion Visit (Week 60).<br>• epis<br>Disallowed:<br>• Do not use CNS- and migraine-related devices (neuromodulation, neurostimulation) until the Completion Visit (Week 60).
Sedatives/hypnotics	• Barbiturates (including Fiorinal®, Fioricet®, or any other combination containing butalbital) are allowed provided a stable dose and regimen have been maintained during the Lead-in Study and expected to be maintained until the Completion Visit (Week 60). These agents may be prescribed when considered medically indicated by the investigator during the study and a stable dose and regimen are expected to be maintained.
Traditional Chinese Patent medicines for the treatment of migraine	• Allowed for the treatment of migraine providing a stable regimen has been maintained during the Lead-in Study and expected to be maintained until the Completion Visit (Week 60).
COVID-19 Vaccine	Allowed during the study with the following guidance if possible:<br>• COVID-19 vaccine should not be given within ±3 days of the IMP infusion.<br>• If the patient has recently received a COVID-19 vaccine, the investigator should judge if the patient can be administered the IMP infusion at the scheduled visit based upon the patient's individual response to the COVID-19 vaccine.
