# Supplementary material for: Long-term tolerability and effectiveness of eptinezumab in Japanese adults with chronic migraine: results of the 60-week open-label SUNSET trial
Source: J Headache Pain. 2025 Nov 22;26(1):275. doi: 10.1186/s10194-025-02214-w (PMC12659320; doi:10.1186/s10194-025-02214-w)
Supplement: Supplementary file 2 — Additional file 2 [file 10194_2025_2214_MOESM2_ESM.pdf]

Statistical Analysis Plan

Interventional, open-label, flexible-dose, long-term extension study to evaluate safety of eptinezumab as preventive treatment in patients with migraine in Japan

Eptinezumab

Trial No.:	19140B
	Protocol edition 2.0
Sponsor:	H. Lundbeck A/S (Lundbeck)<br>2500 Valby (Copenhagen), Denmark
Biostatistician:	PPD
SAP date:	22 August 2024

This document is the property of H. Lundbeck A/S and H. Lundbeck A/S is the holder of any and all related intellectual property rights, including, but not limited to, copyrights. This document is confidential. It is not to be copied or distributed to other parties without prior written authorization from H. Lundbeck A/S.

Table of Contents

List of Panels and Tables **6**

List of Abbreviations and Definitions of Terms **7**

1 Objectives, Endpoints and Estimands **8**

2 Trial Design **10**
2.1 Clinical Trial Report 12

3 COVID-19 .... **13**

4 Definitions **13**
4.1 Definition of Baseline 13
4.2 Definition of Periods 13
4.3 Definition of Withdrawal from Trial 14
4.4 Definition of Migraine and Headache Days 14
4.4.1 Migraine Day 14
4.4.2 Headache Day 15
4.5 Definition of groups and subgroups related to treatment 15

5 Analysis Sets **16**

6 Descriptive statistics **16**

7 Participant Disposition **16**
7.1 Summary of Participant Disposition 16
7.2 Withdrawals 17

8 Demographics and Baseline Characteristics **17**

9 Recent and Concomitant Medication **18**

10 Exposure **18**

11 eDiary Compliance **19**

12 Effectiveness **19**
12.1 General Effectiveness Analysis Methodology 19
12.2 Testing Strategy 20
12.3 Analysis Methodology for the Primary endpoints 20
12.4 Analysis Methodology for the Key Secondary Endpoints 20
12.5 Analysis Methodology of the Secondary and Exploratory Endpoints 20
12.5.1 Maintenance of Therapeutic Effictiveness Estimand 20
12.5.1.1 Rationale for the Maintenance of Therapeutic Effectiveness Estimand 21
12.5.1.2 Estimator 22
12.5.2 Intercurrent Events 22
12.5.3 Effectiveness Analysis 23
12.5.3.1 Continuous Effectiveness Endpoints 23
12.5.3.2 Effectiveness Endpoints Regarding Response 24
12.5.3.3 Other Effectiveness Endpoints 24
12.5.3.4 Subgroup Analyses 24

12.5.4 Rationale for Selected Analysis Method for the Secondary and Exploratory Endpoints....24
12.5.5 Sensitivity Analyses of Secondary Endpoints....25
12.5.5.1 Sensitivity Analyses to assess the impact of missing data regarding MMDs....25
12.5.5.2 Sensitivity Analysis to assess the impact of missing data regarding 50% response on MMDs....25
12.5.6 Selected Analyses by Prior Treatment Group....25

13 Safety Estimand....26
13.1 Estimand for the safety endpoints....26
13.2 Rationale for the safety estimand....27
13.3 Estimator....27

14 Safety....28
14.1 Adverse Events....28
14.1.1 General Methodology for Adverse Events....28
14.1.2 Coding of Adverse Events....28
14.1.3 Classification of Adverse Events....28
14.1.4 Presentation of Adverse Events....28
14.1.5 Presentation of Adverse Events Starting Prior to First Infusion in Trial 19140B....29
14.1.6 Presentation of Treatment-emergent Adverse Events....29
14.1.7 Presentation of Deaths....29
14.1.8 Presentation of Serious Adverse Events....29
14.1.9 Presentation of Adverse Events Leading to Withdrawal from Trial....29
14.1.10 Presentation of Adverse Events Leading to Infusion Interruption or Termination....30
14.1.11 Presentation of Adverse Events of Special Interest....30
14.2 General Methodology for Other Safety Data....30
14.3 Clinical Safety Laboratory Test Data....31
14.3.1 Data Presentation....31
14.3.2 Potential Drug-induced Liver Injury (DILI)....31
14.4 Vital Signs and Weight....32
14.5 ECGs....32
14.6 Other Safety Endpoints....33
14.6.1 Columbia-Suicide Severity Rating Scale (C-SSRS) Scores....33

15 Immunogenicity....33

16 Pharmacokinetic/Pharmacodynamic Analyses....35

17 Data Reviews....35

18 Interim Analyses....35

19 Sample Size Considerations....35

20 Statistical Software....36

21 Changes to Analyses Specified in the Protocol....36

22 Details on Data Handling....37
22.1 Derived Variables....37
22.1.1 eDiary....37

22.1.1.1 Monthly Migraine Days (MMDs)/Monthly Headache Days (MHDs) 37
22.1.1.2 Migraine/Headache Responder Variables 38
22.1.1.3 Migraines/Headaches With Severe Pain Intensity and Headache Episodes/Migraine Attacks 39
22.1.1.4 Monthly Days with Acute Migraine Medication Usage and MMDs/MHDs with Acute Medication Usage 39
22.1.2 Patient Global Impression of Change (PGIC) 40
22.1.3 Most Bothersome Symptom (MBS) 40
22.1.4 Headache Impact Test (HIT-6) 40
22.1.5 Migraine-Specific Quality of Life Questionnaire Version 2.1 (MSQ v2.1) 40
22.1.6 Euroqol 5 Dimension – 5 Levels (EQ-5D-5L) 42
22.1.7 Health Care Resource Utilization (HCRU) 42
22.1.8 Work Productivity and Activity Impairment: Migraine (WPAI:M) 42
22.2 Assigning Data to Visits 43
22.2.1 Clinical Outcome Assessments (COAs) - Scales 43
22.2.2 Safety Variables 44
22.3 Handling Missing or Incomplete Dates/Times 46
22.3.1 Missing Headache Stop Date and Time 46
22.3.2 Withdrawal Date 46
22.3.3 Medical Disorder Start and Stop Dates 46
22.3.4 Medication Start and Stop Dates 46
22.3.5 Adverse Event Start and Stop Dates 47
22.4 Data with Multiple Records 48
22.4.1 Dose Changes in Medication 48
22.4.2 Changes in Intensity or Seriousness of Adverse Events 48
References 50

Appendices

Appendix I Statistical Analysis Plan Authentication and Authorization 51
Appendix II Trial Procedures and Assessments 53
Appendix III SAS® Code 59
Appendix IV PCS Criteria 62
Appendix V Adverse Events of Special Interest 66

List of Panels and Tables

Panel 1 Objectives, Endpoints and Estimands 8
Panel 2 Trial Design 12
Panel 3 C-SSRS Scores 33
Panel 4 MSQ v.2.1 Domains 41
Panel 5 MSQ v.2.1 Item Values 41
Panel 6 Derivation of MSQ v.2.1 Domain Scores 42
Panel 7 Derivation of WPAI:M sub-scores 43
Panel 8 Visit Windows – PGIC, MBS, MSQ v2.1 43
Panel 9 Visit Windows – HIT-6, EQ-5D-5L, HCRU, WPAI:M 43
Panel 10 Visit Windows – Laboratory tests, ECG 44
Panel 11 Visit Windows – Vital Signs (Pulse rate, Blood Pressure, Temperature) 45

Table 1 Trial Procedures and Assessments 54
Table 2 PCS Criteria for Clinical Safety Laboratory Tests 63
Table 3 PCS Criteria for Vital Signs, Weight/BMI, and Waist Circumference 65
Table 4 PCS Criteria for ECG Parameters 65
Table 5 Adverse Events of Special Interest 67

List of Abbreviations and Definitions of Terms

ADA	anti-drug antibody
AE	adverse event
AESI	adverse event of special interest
APES	all-participants-enrolled set
APTS	all-participants-treated set
BMI	body mass index
CGRP	calcitonin gene-related peptide
COA	Clinical outcome assessment
C-SSRS	Columbia-Suicide Severity Rating Scale
DILI	drug-induced liver injury
DMC	Data Monitoring Committee
ECG	electrocardiogram
EQ-5D-5L	Euroqol 5 Dimensions
FAS	full-analysis set
HCRU	Health Care Resource Utilization
HIT-6	Headache Impact Test
IMP	investigational medicinal product
MBS	Most Bothersome Symptom
MedDRA	Medical Dictionary for Regulatory Activities
MMRM	mixed model for repeated measurements
MSQ v2.1	Migraine-Specific Quality of Life Questionnaire Version 2.1
NAb	Neutralizing antibody
PCS	potentially clinically significant
PGIC	Patient Global Impression of Change
PRO	participant reported outcome
REML	restricted maximum likelihood
SAE	serious adverse event
SAS®	statistical software package from the SAS® Institute
SOC	system organ class
TEAE	treatment-emergent adverse event
WPAI:M	Work Productivity and Activity Impairment: Migraine

1 Objectives, Endpoints and Estimands

The trial objectives, endpoints and estimands are summarized in Panel 1.

Panel 1 Objectives, Endpoints and Estimands

<table>
<tr><th>Objectives</th><th>Endpoints</th></tr>
<tr><td>Primary Objectives
• To evaluate the long-term safety and tolerability of eptinezumab</td><td>Safety Endpoints
• Adverse events
• Absolute values and changes from Baseline (Baseline of Trial 19140B) in clinical safety laboratory test values, vital signs, weight, and ECG parameter values
• Potentially clinically significant clinical safety laboratory test values, vital signs, weight changes, and ECG parameter values
• Development of specific anti-eptinezumab antibodies (ADA) including neutralizing antibodies (NAbs)
• Columbia-Suicide Severity Rating Scale (C-SSRS) score</td></tr>
<tr><td colspan="2">Safety Estimand:
The estimand will be the proportion of patients experiencing a treatment emergent adverse event (TEAE) among patients with chronic migraine, who have been treated with eptinezumab 100 mg/300 mg every 12 weeks for 60 weeks, with or without the use of other preventive treatments, and regardless of infusion interruption due to other reasons than TEAE and withdrawal from trial due to other reasons than TEAE.

The estimand for the primary endpoint is described by the following attributes:
– The population of interest is patients with chronic migraine
– The endpoint to be considered is to experience at least one TEAE during 60 weeks of treatment with eptinezumab.
– The treatment condition of interest is treatment with eptinezumab 100 mg/300 mg for 60 weeks, with or without use of other preventive migraine medications.
– Intercurrent events are the following:
- infusion interruption due to other reasons than TEAE, which will be addressed using a treatment policy strategy
- withdrawal from trial due to other reasons than TEAE, which will be handled using a while on treatment strategy
– The population level summary will be the proportion of patients experiencing at least one TEAE during 60 weeks of treatment with eptinezumab.</td></tr>
<tr><td>Secondary Objectives
• To evaluate the maintenance of the therapeutic effect of eptinezumab on:
– prevention of migraine
– health-related quality of life</td><td>Secondary endpoints
• Change from Baseline (Baseline of Trial 19140A) in the number of MMDs (Weeks 1-4, 5-8, 9-12, 13-16, 17-20, 21-24, 25-28, 29-32, 33-36, 37-40, 41-44, 45-48, 49-52, 53-56, 57-60)
• Change from Baseline in the number of MMDs (Weeks 1-12, 13-24, 25-36, 37-48, 49-60)
• Response: ≥50% reduction from Baseline in MMDs (Weeks 1-4, 5-8, 9-12, 13-16, 17-20, 21-24, 25-28, 29-32, 33-36, 37-40, 41-44, 45-48, 49-52, 53-56, 57-60)
• Response: ≥50% reduction from Baseline in MMDs (Weeks 1-12, 13-24, 25-36, 37-48, 49-60)</td></tr>
</table>

**Objectives**	**Endpoints**
	• Change from Baseline in the HIT-6 score at Weeks 4, 8, 12, 16, 20, 24, 28, 32, 36, 40, 44, 48, 52, 56, 60<br>• Change from Baseline in the Health-Related Quality of Life (EQ-5D-5L) Visual Analogue Scale (VAS) score at Weeks 4, 8, 12, 16, 20, 24, 28, 32, 36, 40, 44, 48, 52, 56, 60<br>• Patient Global Impression of Change (PGIC) score at Weeks 12, 24, 36, 48, 60<br>• Change from Baseline in the Most Bothersome Symptom (MBS) score at Weeks 12, 24, 36, 48, 60

Estimands for Secondary endpoints:

- Maintenance of therapeutic effectiveness estimand:

The maintenance of therapeutic effectiveness estimand will be mean change from Baseline (Baseline of Trial 19140A) in MMDs across Weeks 49-60 in patients with chronic migraine, who have received double blinded treatment with placebo, eptinezumab 100 mg or eptinezumab 300 mg for 12 weeks beforehand, now treated with eptinezumab 100 mg/300 mg every 12 weeks for 60 weeks, in the hypothetical scenario where no other long-acting anti-CGRP treatment are available and where all patients manage to continue treatment, with or without the use of other preventive treatments, and regardless of infusion interruption.

The maintenance of therapeutic effectiveness estimand is described by the following attributes:

- The population of interest is patients with chronic migraine who beforehand have received double blinded treatment with placebo, eptinezumab 100 mg or 300 mg for 12 weeks
- The endpoint to be considered is the change from Baseline in MMDs (Weeks 49-60)
- The treatment condition of interest is treatment with eptinezumab 100 mg/300 mg every 12 weeks for 60 weeks (where eptinezumab 300 mg is assigned to patients not achieving 50% reduction in MMDs compared to Baseline when receiving eptinezumab 100 mg during Weeks 1-12), with or without the use of other preventive migraine medications except other long-acting anti-CGRPs
- The population level summary will be the mean change from Baseline in MMDs for Weeks 49-60
- Intercurrent events are:
  - use of other long-acting anti-CGRP treatments, which will be handled using a hypothetical strategy to assess the effectiveness in a scenario where no other long-acting anti-CGRPs are available
  - infusion interruption, which will be handled using a treatment policy strategy
  - withdrawal from trial, which will be handled using a hypothetical strategy, assuming a scenario where all patients will continue to receive treatment with eptinezumab for all of the 60 weeks

- Other continuous endpoints: Other continuous endpoints than change from Baseline in MMDs will be handled similarly to the maintenance of therapeutic effectiveness estimand.

- 50% Response endpoint: The proportion of patients with at least 50% reduction from Baseline in MMDs (Weeks 49-60) will be handled similarly to the maintenance of therapeutic effectiveness estimand described above.

- Other response endpoints: Endpoints based on response variables are handled similarly to the 50% Response endpoint.

**Exploratory Objectives**	**Exploratory Endpoints**
• To evaluate the long-term exposure of eptinezumab<br>• To evaluate the maintenance of the therapeutic effect of eptinezumab on:	• Eptinezumab plasma concentrations during long-term treatment<br>• Change from Baseline (Baseline of Trial 19140A) in the Migraine-Specific Quality of Life (MSQ v2.1) sub-scores

Objectives	Endpoints
– healthcare resource utilization, and<br>– work productivity	(Role Function-Restrictive, Role Function-Preventive, Emotional Function) at Weeks 12, 24, 36, 48, 60<br>• Health Care Resources Utilization (HCRU) at Baseline and Weeks 4, 8, 12, 16, 20, 24, 28, 32, 36, 40, 44, 48, 52, 56, 60<br>• Change from Baseline in the Work Productivity and Activity Impairment Questionnaire: Migraine (WPAI:M) subscores (Absenteeism, Presenteeism, Work productivity loss, Activity impairment) at Weeks 4, 8, 12, 16, 20, 24, 28, 32, 36, 40, 44, 48, 52, 56, 60<br>• Response: ≥75% reduction from Baseline in MMDs (Weeks 1-4, 5-8, 9-12, 13-16, 17-20, 21-24, 25-28, 29-32, 33-36, 37-40, 41-44, 45-48, 49-52, 53-56, 57-60)<br>• Response: ≥75% reduction from Baseline in MMDs (Weeks 1-12, 13-24, 25-36, 37-48, 49-60)<br>• Change from Baseline in the number of MHDs (Weeks 1-4, 5-8, 9-12, 13-16, 17-20, 21-24, 25-28, 29-32, 33-36, 37-40, 41-44, 45-48, 49-52, 53-56, 57-60)<br>• Change from Baseline in the number of MHDs (Weeks 1-12, 13-24, 25-36, 37-48, 49-60)<br>• Response: ≥50% reduction from Baseline in MHDs (Weeks 1-4, 5-8, 9-12, 13-16, 17-20, 21-24, 25-28, 29-32, 33-36, 37-40, 41-44, 45-48, 49-52, 53-56, 57-60)<br>• Response: ≥50% reduction from Baseline in MHDs (Weeks 1-12, 13-24, 25-36, 37-48, 49-60)<br>• Response: ≥75% reduction from Baseline in MHDs (Weeks 1-4, 5-8, 9-12, 13-16, 17-20, 21-24, 25-28, 29-32, 33-36, 37-40, 41-44, 45-48, 49-52, 53-56, 57-60)<br>• Response: ≥75% reduction from Baseline in MHDs (Weeks 1-12, 13-24, 25-36, 37-48, 49-60)<br>• Change from Baseline in the proportion of migraine attacks with severe pain intensity (Weeks 1-12, 13-24, 25-36, 37-48, 49-60)<br>• Change from Baseline in the proportion of headache episodes with severe pain intensity (Weeks 1-12, 13-24, 25-36, 37-48, 49-60)<br>• Change from Baseline in monthly migraine attacks (Weeks 1-12, 13-24, 25-36, 37-48, 49-60)<br>• Change from Baseline in monthly headache episodes (Weeks 1-12, 13-24, 25-36, 37-48, 49-60)<br>• Change from Baseline in monthly days with use of acute migraine medication (Weeks 1-4, 5-8, 9-12, 13-16, 17-20, 21-24, 25-28, 29-32, 33-36, 37-40, 41-44, 45-48, 49-52, 53-56, 57-60)

2 Trial Design

This is an interventional, multi-site, Open-label Phase III trial (Trial 19140B) conducted in participants in Japan, to evaluate the long-term safety of eptinezumab in participants with migraine who have completed the Primary Outcome Visit in the Lead-in Trial (Trial 19140A)

and are eligible for preventive treatment. Only participants enrolled in Trial 19140A under Trial 19140A Protocol version 2.0 or earlier where allowed to enter Trial 19140B.

The target population for this trial are participants diagnosed with chronic migraine as outlined in the IHS ICHD-3 guidelines,[1] confirmed at screening into the Trial 19140A.

Investigators and participants will be informed about which treatment the participants received in the Trial 19140A only after the last participant has completed the Open-label Trial 19140B and Trial 19140A has been unblinded.

The Baseline Visit of Trial 19140B will be the same as Visit 5 (Primary Outcome Visit) in the Trial 19140A.

The total trial duration from the Baseline Visit of Trial 19140B to the Safety Follow-up Visit is approximately 68 weeks and includes an Open-label Treatment Period (60 weeks) and a Safety Follow-up Period (8 weeks).

Participants will receive IMP at the Baseline Visit of Trial 19140B and once every 12 weeks (5 infusions in total) by intravenous infusions of 30 minutes (+15 minutes).

All participants will receive eptinezumab 100 mg infusion at the Baseline Visit of Trial 19140B.

At Week 12 (Visit 4), participants that do not have a treatment response of at least 50% reduction of MMDs as compared to the Baseline Visit of Trial 19140A will have their eptinezumab dose increased to 300 mg. This dose increase to 300 mg is only done at Visit 4, if applicable.

After Visit 4, all participants will continue receiving the same eptinezumab dose for the remainder of the trial, except for participants on eptinezumab 300 mg that have tolerability issues (who will be allowed to switch to eptinezumab 100 mg again once between Visits 5 to 13 [inclusive] and will remain on eptinezumab 100 mg for the remainder of the trial).

Participants will complete a daily headache eDiary from the Baseline Visit of Trial 19140B until the Completion/Withdrawal Visit.

During the IMP Visits, assessments of safety will be performed before and after the infusion. At these visits, AEs will be collected as well as clinical safety laboratory tests, ECG, weight, vital signs and blood samples for eptinezumab and ADA quantification. On the IMP Visit day, participant reported outcomes (PROs) must be completed prior to infusion. Participants must ensure to complete eDiary recording of headaches which ended prior to infusion (i.e., for headaches which are ongoing or not yet recorded in the eDiary).

Participants who complete the trial will attend a Safety Follow-up Visit, 20 weeks after the last IMP Visit (date when the last dose of IMP was administered).

Participants who withdraw, except for those who withdraw their consent, will be asked to attend a Withdrawal Visit as soon as possible and a further Safety Follow-up Visit scheduled 20 weeks after the last IMP Visit (date when the last dose of IMP was administered).

An independent Safety Data Monitoring Committee (DMC) will regularly monitor the participants' safety data according to the DMC Charter.

An overview of the trial is presented in Panel 2 and the scheduled trial procedures and assessments are summarized in Appendix II. See also the Clinical Trial Protocol for further details on the trial design.

Panel 2 Trial Design

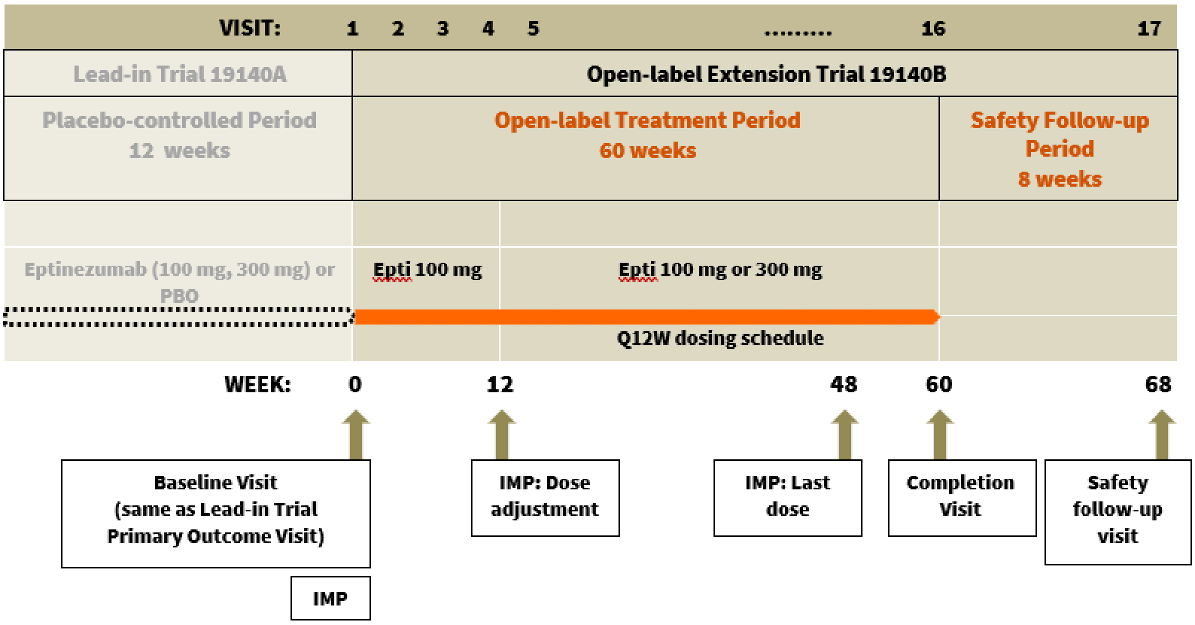

Participants are enrolled in Trial 19140B after completion of 12 weeks of blinded treatment in the Trial 19140A. Trial 19140B consists of an open-label treatment period of 60 weeks (5 infusions), and a safety follow-up period (8 weeks). IMP (eptinezumab 100 mg or 300 mg) will be administered by intravenous infusion at the Baseline Visit of Trial 19140B and once every 12 weeks. At Week 60, participants will complete the Completion Visit and will return to the clinic 8 weeks later for a Safety Follow up Visit.

2.1 Clinical Trial Report

The data collected throughout the trial will be used for generating the output to be included in the *Clinical Trial Report* (CTR). Since Trial 19140A will still be blinded by the completion of Trial 19140B, not all outputs for the CTR can be produced by the end of Trial 19140B. This means that only outputs not depending on the blinded treatment from the Trial 19140A and outputs with no unblinding potential of Trial 19140A will be produced by the end of Trial

19140B. The remaining tables, listings and figures for the CTR will be generated after unblinding Trial 19140A. Hence the CTR can only be generated after unblinding Trial 19140A.

This *Statistical Analysis Plan* describes the outputs prepared for the CTR.

3 COVID-19

For this trial, all participants are considered to have been enrolled after the beginning of the COVID-19 outbreak.

The following information is collected with regards to COVID-19:

- Whether a visit was done remotely due to COVID-19
- Whether a visit was not done due to COVID-19
- Whether participant withdrew due to the COVID-19 situation
- Whether participant got diagnosed with COVID-19

Specific output addressing the impact of COVID-19 for this trial is specified in the relevant sections below.

4 Definitions

4.1 Definition of Baseline

For safety endpoints, unless otherwise specified, the Baseline will be defined as the valid measurements taken prior to IMP at the Baseline Visit of Trial 19140B which corresponds to Week 12 Visit in Trial 19140A.

For effectiveness endpoints based on the eDiary, the first 28 days of the Screening Period of Trial 19140A will be used as Baseline.

For other effectiveness endpoints related to PROs, the valid measurements captured prior to IMP at the Baseline Visit of Trial 19140A will be used as Baseline.

For other endpoints, unless otherwise specified, the Baseline will be defined as the valid measurements taken prior to IMP at the Baseline Visit of Trial 19140B which corresponds to Week 12 Visit in Trial 19140A.

4.2 Definition of Periods

The trial consists of the following period:

- *Treatment Period* (68 weeks): Starts at Week 0 Visit and continues up to and including Week 68. Hereby this period covers the entire Open-label Trial by covering both the Open-label Treatment Period and Safety Follow-up Period shown in Panel 2.

4.3 Definition of Withdrawal from Trial

Participants who withdrew from treatment and the scheduled visits will be described as *withdrawn from trial*. The complementary group will be described as *completed*.

4.4 Definition of Migraine and Headache Days

For the purpose of endpoint derivations, the following definitions of migraine and headache days will be used.

In this document, the term headache will encompass both headaches and migraine headaches. Migraine headaches (henceforth simply referred to as "migraines") are a subgroup of headaches with characteristics outlined below in Section 4.4.1. Using this definition, we have that all migraines are headaches, but not all headaches are migraines.

Headaches will be self-reported by the participant using an electronic diary (eDiary). The eDiary is split into two parts, the headache diary and the evening diary. The headache diary is the part of the eDiary where the start and stop date and times of experienced headaches are recorded along with headache characteristics used for classifying whether headaches are migraines, and the evening diary is the part of the eDiary that participants are expected to complete each day during the trial regardless of whether or not they experienced a headache on that day. The evening diary captures use of acute medication, whether the participant experienced any headache that day, and whether the participant experienced aura without headache on that day. The possible acute medication choices are: Ergotamine, Triptan, Analgesic, Opioid, Combination Analgesic, TCMs, Antiemetics (metoclopramide, domperidone), Analginum, Antipyrine, Tolfenamic acid, None (Exclusive).

The eDiary reported headache characteristics will be derived using the rules for handling missing data described in Section 22.1.1.

4.4.1 Migraine Day

A migraine day is defined as any day with a headache that meets criteria A, B, or C listed below:

A. All of the following criteria:

1. Lasted 4 hours or more
2. Had at least 2 of the following:
    a. Unilateral location
    b. Pulsating quality
    c. Moderate or severe pain intensity

d. Aggravation by or causing avoidance of routine physical activity

3. Had at least 1 of the following:

   a. Nausea

   b. Vomiting

   c. Photophobia and phonophobia

B. A headache that lasted 30 minutes or more and participants had an aura with the headache

C. Participant took acute medication to treat the headache because they believed they were having a migraine

4.4.2 Headache Day

A headache day is defined as any day with a headache that meets one of the following criteria:
- Lasted 30 minutes or more
- Meets the criteria for being a migraine, i.e. fulfils either criteria A, B, or C above (see Section 4.4.1)

4.5 Definition of groups and subgroups related to treatment

As all participants in this Open-label trial will follow the same treatment regimen, one treatment group is considered, comprising all participants:
- *EPTI 100/300 mg*: Participants who receive eptinezumab 100 mg throughout the trial and participants for whom the dose is increased to eptinezumab 300 mg at Week 12 Visit, no matter if the dose is decreased to eptinezumab 100 mg again later in the trial

Some outputs and analyses will be based on the treatment that the participants received during Trial 19140A. The Previous Treatment groups will be defined as follows:
- *PBO - EPTI:* Participants receiving placebo in Trial 19140A and eptinezumab 100 mg/300 mg in Trial 19140B
- *EPTI 100 mg - EPTI:* Participants receiving eptinezumab 100 mg in Trial 19140A and eptinezumab 100 mg/300 mg in Trial 19140B
- *EPTI 300 mg - EPTI:* Participants receiving eptinezumab 300 mg in Trial 19140A and eptinezumab 100 mg/300 mg in Trial 19140B

Selected analyses will be repeated for the subgroup of participants that receive eptinezumab 300 mg during Trial 19140B (“EPTI 300 mg at least once”). The subgroup will be defined as participants that at some point during Trial 19140B receive eptinezumab 300 mg, no matter if the dose is decreased to eptinezumab 100 mg again later in the trial.

5 Analysis Sets

The following analysis set(s) will be used for the analyses:

- *all-participants-enrolled set* (APES) – all participants who have completed the Lead-in Trial 19140A and are enrolled into the Open-label Trial 19140B
- *all-participants-treated set* (APTS) – all participants in the APES who received IMP in the Open-Label Trial 19140B
- *full-analysis set (FAS)* – all participants in the APTS who had a valid Baseline assessment of MMDs (based on the eDiary data from the first 28 days of the Screening Period of Trial 19140A) and at least one valid post-baseline assessment of MMDs in Trial 19140B

The participants and data will be classified into the analysis sets according to these definitions at a *Classification Meeting* held after the trial database has been released, but prior to unblinding of Trial 19140A.

Unless otherwise specified, all outputs will be based on APTS except from effectiveness analyses and eDiary Compliance which will be based on FAS.

6 Descriptive statistics

Unless otherwise specified, summary statistics (n, arithmetic mean, standard deviation [SD], median, lower and upper quartiles, minimum and maximum values) will be presented for continuous variables, and counts and, if relevant, percentages will be presented for categorical variables.

Unless otherwise specified, data listings will include site, treatment group, participant screening number, sex, age, race, and Baseline weight. Participants in the treatment subgroup “EPTI 300 mg at least once” will be flagged.

7 Participant Disposition

7.1 Summary of Participant Disposition

Participant disposition will be summarized overall and by site. The summaries will include the number of participants in each analysis set defined in Chapter 5, and the number of participants in the APTS who completed or withdrew.

To assess the potential impact of COVID-19 on the visit structure, a table will be provided for the visits that are changed from on site to remote. The summary will be based on APTS. Furthermore, a summary of visits not done due to COVID-19 will be provided.

7.2 Withdrawals

The number of participants who withdrew from trial will be summarized by primary reason for withdrawal, and by all reasons for withdrawal. The same summaries will be produced by previous treatment group.

Participants who withdrew from trial will be listed and the listing will include the number of days in the trial until withdrawal, previous treatment group, time since first IMP in Trial 19140A, time since last IMP in Trial 19140B, the primary reason for withdrawal, and all reasons for withdrawal.

Kaplan-Meier failure plots of time to withdrawal from trial will be presented overall and by previous treatment group. The time will be calculated from the date of first dose of IMP in Trial 19140B to the date of completion or withdrawal in Trial 19140B. Participants who completed will be regarded as censored at the Completion Visit or Week 60, whichever comes first.

8 Demographics and Baseline Characteristics

Demographics (sex, age, race); baseline characteristics (height, weight, and BMI); baseline disease characteristics; and baseline effectiveness variables collected in Trial 19140A will be summarized overall and by previous treatment. Furthermore, baseline characteristic (height, weight, and BMI) will be presented by sex.

The disease characteristics captured at Screening Visit of Trial 19140A comprise age at diagnosis of migraine and at first onset of migraine and age group (≤ 21 years, > 21 years) at diagnosis of migraine, duration and duration group (≤ 15 years, > 15 years) from date of migraine diagnosis to Baseline of Trial 19140A, whether the participant suffers from fully reversible aura symptoms and if yes, which aura symptoms, whether the participants experiences aura symptoms without headache and if yes, which aura symptoms, for women whether migraines started before or after menarche and for men whether migraines started before or after puberty, whether the start of migraine was related to any event, and whether the participant suffer from Medication Overuse Headache.

The eDiary reported Baseline headache characteristics captured during Screening period of Trial 19140A that will be summarized are the Baseline MMDs, Baseline MHDs, monthly migraine attacks and headache episodes at Baseline, the proportion of headaches and migraines with severe pain intensity, Baseline MMDs with use of acute medication, and monthly days with use of acute medication.

The PRO Baseline variables that will be summarized are HIT-6 total score – summarized both as a continuous variable and as a categorical variable displaying the percentage and counts of the life impact categories (see Section 22.1.4) – MSQ v2.1 subscores, EQ-5D-5L VAS score, HCRU, WPAI:M subscores, and counts and percentages of MBS symptom chosen at screening.

Concurrent as well as relevant past medical, neurological, and psychiatric disorders will be coded using the *Medical Dictionary for Regulatory Activities* (MedDRA) and summarized overall and by previous treatment group.

A concurrent medical, neurological, or psychiatric disorder is a disorder that is ongoing at the Screening Visit of 19140A. A past medical, neurological, or psychiatric disorder is a disorder that ended prior to the Screening Visit of 19140A.

Social history will be summarized.

Summaries of prior preventive treatment failure medications within 5 years prior to the screening visit at Trial 19140A will be presented. The number and percentage of participants, who experienced each type of prior treatment failure (lack of efficacy, safety/tolerability, or contraindication) will also be presented as well as the number of previous treatment failures.

9 Recent and Concomitant Medication

Recent and concomitant medication will be coded using the *WHO Drug Dictionary* (WHO-DD).

Medications will be classified according to the start and stop time and summarized by anatomical therapeutic chemical (ATC) code, and generic drug name:

- Concomitant medication continued after or started at or after first dose of IMP in Trial 19140B

The summary will be repeated for preventive and acute migraine medications, which will be identified via a clinical review of the coded medication data in the eCRF.

All disallowed medications will be listed based on the APES. The listing will include the generic drug name, indication, time since first IMP, the duration, the start and stop dates, and dosing information.

The eDiary reported medications will be handled separately. In the evening diary, participants are asked each day to fill out whether they used any of the following medications during that day: Ergotamine, Triptan, Analgesic, Opioid, Combination analgesic, TCMs (Traditional Chinese Medicines), Antiemetics (metoclopramide, domperidone), Analginum, Antipyrine, and Tolfenamic acid. The number of participants taking each of the medication types during Weeks 1-60 of Trial 19140B will be presented.

10 Exposure

For each infusion visit, information related to infusion completed as planned (yes/no), infusion temporarily interrupted (yes/no), and infusion lasted longer than 30 (+15) minutes (yes/no), as well as descriptive statistics for the duration of infusions including any infusion interruptions and duration of temporary interruptions will be summarized.

In addition, all infusion data will be listed, i.e. the start and stop time of the infusion and the duration of the infusion.

For each infusion visit, the number of participants receiving an infusion will be summarized.

Due to the possibility of increasing the dose to eptinezumab 300 mg at Week 12 Visit and the possibility to decrease the dose to eptinezumab 100 mg again at a later point, different dosing sequences can occur. The number of participants, who have followed each of the observed dosing sequences, will be summarized.

The number of participants in Trial 19140B who received at least 4 infusions of eptinezumab including infusions with eptinezumab in Trial 19140A and were exposed to eptinezumab for more than 365 days will be summarized. For this the exposure will be calculated from the day of first exposure to eptinezumab in Trial 19140A or Trial 19140B to the last day of contact in Trial 19140B.

Participants were classified as Non-responders if reduction MMDs from Baseline of Trial 19140A to Weeks 1-12 of Trial 19140B was less than 50%. Participants classified as Non-responders were hereafter increased from eptinezumab 100 mg to eptinezumab 300 mg.

The number of Non-responders will be provided based on APTS.

A list of participants who were wrongly classified as Non-responders will be provided.

11 eDiary Compliance

The rate of days where the eDiary has been missed within each 4-week interval will be summarized and presented by 4-week interval. Furthermore, the number of participants missing 14 days or more in a 28 day period will be presented by 4-week interval. Participants withdrawing will not be counted after withdrawal. The summary will include the number of participants reporting in the eDiary any day within each 4-week interval.

A day where the eDiary has been completed is defined as a day where the participant had a headache occurring according to the headache diary, or where the participant did confirm in the evening diary that the participant did not have any headache. A day where the eDiary was not completed will be defined as missed.

The summaries will be based on the FAS.

12 Effectiveness

12.1 General Effectiveness Analysis Methodology

All confidence intervals (CIs) will be two-sided 95% CIs.

12.2 Testing Strategy

No formal testing will be done.

12.3 Analysis Methodology for the Primary endpoints

There are no primary effectiveness endpoints. The primary endpoints are safety endpoints which are described in Chapter 14.

12.4 Analysis Methodology for the Key Secondary Endpoints

There are no key secondary effectiveness endpoints.

12.5 Analysis Methodology of the Secondary and Exploratory Endpoints

12.5.1 Maintenance of Therapeutic Effictiveness Estimand

The maintenance of therapeutic effectiveness estimand will be mean change from Baseline in MMDs across Weeks 49-60 in patients with chronic migraine, who have received double blinded treatment with placebo, eptinezumab 100 mg, or eptinezuamb 300 mg for 12 weeks beforehand, now treated with eptinezumab 100 mg/300 mg every 12 weeks for 60 weeks, in the hypothetical scenario where no other long-acting anti-CGRP treatment are available and where all patients manage to continue treatment, with or without the use of other preventive treatments, and regardless of infusion interruption.

Clinical Question of interest

The clinical question of interest is the change from Baseline in MMDs within 49-60 weeks of treatment with eptinezumab 100 mg/300 mg for every 12 weeks.

Attributes of The Maintenance of Therapeutic Effectiveness Estimand

The maintenance of therapeutic effectiveness estimand is described by the following attributes:

- The **treatment condition of interest** is treatment with eptinezumab 100 mg/300 mg every 12 weeks for 60 weeks (where eptinezumab 300 mg is assigned to patients not achieving 50% reduction in MMDs compared to Baseline when receiving eptinezumab 100 mg during Weeks 1-12), with or without the use of other preventive migraine medications except other long-acting anti-CGRPs
- The **population of interest** is patients with chronic migraine, who beforehand have received double blinded treatment with placebo, eptinezumab 100 mg or eptinezumab 300 mg for 12 weeks
- The **endpoint to be considered** is the change from Baseline in MMDs (Weeks 49-60)
- The **intercurrent events** will be:

- use of other long-acting anti-CGRP treatments, which will be handled using a hypothetical strategy
- infusion interruption which will be addressed using a treatment policy strategy
- withdrawal from trial which will be handled using a hypothetical strategy

- The **population level summary** will be the mean change from Baseline in MMDs for Weeks 49-60

12.5.1.1 Rationale for the Maintenance of Therapeutic Effectiveness Estimand

Endpoint

The number of MMDs is considered a good measure of the impact of treatment with eptinezumab every 12 weeks, since it captures an important part of the symptoms for chronic migraine. To evaluate the ability to maintain therapeutic effectiveness, the latest 12 weeks interval in the trial following IMP (Weeks 49-60) is chosen as the timepoint to consider. Therefore, change from Baseline in MMDs across Weeks 49-60 is chosen as the endpoint for the maintenance of therapeutic effectiveness estimand.

Treatment Condition of Interest

The use of preventive migraine medication except other long-acting anti-CGRPs is included in the treatment condition of interest because eptinezumab is expected to be used with or without other preventive medications by patients suffering from chronic migraine. Furthermore, though use of preventive medication could influence the number of MMDs, the observed effect would reflect what the patients will experience when using the drug.

Intercurrent Events

The following intercurrent events, occurring after treatment initiation and potentially affecting either the interpretation or the existence of the measurements associated with the clinical question of interest, are considered:

- Use of other long-acting anti-CGRP treatments:
  The use of other long-acting anti-CGRPs could potentially affect the observed effectiveness of eptinezumab. To investigate a scenario where other anti-CGRP treatments would not be available to the patients, the intercurrent event use of other long-acting anti-CGRP is handled by a hypothetical strategy.
- Infusion interruption:
  If an IMP is stopped prematurely (either temporarily or completely) the consequence is that the participant did not receive all of the intended dose of eptinezumab and it could be expected that the potential effect of eptinezumab could be decreased due to the lower dose. To be conservative, a treatment policy strategy is used to address this.
- Withdrawal from trial:
  If a participant withdraw from trial the consequence will be that no further effectiveness data will be collected after the withdrawal visit. To address a scenario where all participants continue treatment with eptinezumab for the entire period of 60 weeks, the

intercurrent event will be addressed using a hypothetical strategy. It is assumed that participants who withdraw from trial would have continued their current trajectory in a manner similar to other participants that continue to receive IMP.

12.5.1.2 Estimator

The intercurrent event Use of other long-acting anti-CGRPs is handled using a hypothetical strategy by removing the data collected after the use of other long-acting anti-CGRPs as described in Section 22.1.1.1, in case we have any records of that. Since effectiveness data will not be collected after the withdrawal visit and the data is modelled using MMRM as described below, no further actions are needed in the estimation to account for the intercurrent event of withdrawal from trial. Since the intercurrent event Infusion interruption will be addressed using a treatment policy strategy, effectiveness data will be used despite of the intercurrent event, and hence no further actions are needed in the estimation to account for the intercurrent event.

Change from Baseline in MMDs Weeks 49-60 will be analysed using a restricted maximum likelihood (REML)-based mixed model for repeated measures (MMRM) approach. The analysis will be performed using all available monthly scores derived as specified in Section 22.1.1.1. Below, the term “month” refers to 4-week periods.

The model will include the following fixed effects: month (Weeks 1-4, Weeks 5-8, Weeks 9-12, Weeks 13-16, Weeks 17-20, Weeks 21-24, Weeks 25-28, Weeks 29-32, Weeks 33-36, Weeks 37-40, Weeks 41-44, Weeks 45-48, Weeks 49-52, Weeks 53-56, Weeks 57-60) as a factor, Baseline MMDs as continuous covariate and baseline score-by-month interaction. An unstructured variance structure will be used to model the within-participant errors.
If, unexpectedly, this analysis fails to converge, the following variance structures will be tried out in the following order: Toeplitz (TOEP), Compound Symmetry (CS). The first to converge will be applied. The Kenward-Roger approximation will be used to estimate denominator degrees of freedom.

The change from Baseline in MMDs Weeks 49-60 will be estimated as the average from the least squares estimates from the MMRM for the monthly effects for Weeks 49-52, Weeks 53-56 and Weeks 57-60.

The SAS® code for the analysis is shown in Appendix III.

12.5.2 Intercurrent Events

The number of participants who experience the intercurrent events for the Maintenance of Therapeutic Effectiveness Estimand (“Use of other long-acting anti-CGRPs”, “Infusion interruption” and “Withdrawal from Trial”) will be summarized.

12.5.3 Effectiveness Analysis

12.5.3.1 Continuous Effectiveness Endpoints

The following continuous secondary and exploratory effectiveness endpoints will be analysed using the same methodology as described in the Maintenance of Therapeutic Effectiveness Estimand in Section 12.5.1.2, with the appropriate baseline values and the appropriate contrast statement.

- Change from Baseline in the number of MMDs (Weeks 1-4, 5-8, 9-12, 13-16, 17-20, 21-24, 25-28, 29-32, 33-36, 37-40, 41-44, 45-48, 49-52, 53-56, 57-60)
- Change from Baseline in the number of MMDs (Weeks 1-12, 13-24, 25-36, 37-48, 49-60)
- Change from Baseline in the HIT-6 score at Weeks 4, 8, 12, 16, 20, 24, 28, 32, 36, 40, 44, 48, 52, 56, 60
- Change from Baseline in the Health-Related Quality of Life (EQ-5D-5L) Visual Analogue Scale (VAS) score at Weeks 4, 8, 12, 16, 20, 24, 28, 32, 36, 40, 44, 48, 52, 56, 60
- Patient Global Impression of Change (PGIC) score at Weeks 12, 24, 36, 48, 60
- Change from Baseline in the Most Bothersome Symptom (MBS) score at Weeks 12, 24, 36, 48, 60
- Change from Baseline in the Migraine-Specific Quality of Life (MSQ v2.1) sub-scores (Role Function-Restrictive, Role Function-Preventive, Emotional Function) (at Weeks 12, 24, 36, 48, 60)
- Change from Baseline in the Work Productivity and Activity Impairment Questionnaire: Migraine (WPAI:M) sub-scores (Absenteeism, Presenteeism, Work productivity loss, Activity impairment) (at Weeks 4, 8, 12, 16, 20, 24, 28, 32, 36, 40, 44, 48, 52, 56, 60)
- Change from Baseline in the number of MHDs (Weeks 1-4, 5-8, 9-12, 13-16, 17-20, 21-24, 25-28, 29-32, 33-36, 37-40, 41-44, 45-48, 49-52, 53-56, 57-60)
- Change from Baseline in the number of MHDs (Weeks 1-12, 13-24, 25-36, 37-48, 49-60)
- Change from Baseline in the proportion of migraine attacks with severe pain intensity (Weeks 1-12, 13-24, 25-36, 37-48, 49-60)
- Change from Baseline in the proportion of headache episodes with severe pain intensity (Weeks 1-12, 13-24, 25-36, 37-48, 49-60)
- Change from Baseline in monthly migraine attacks (Weeks 1-12, 13-24, 25-36, 37-48, 49-60)
- Change from Baseline in monthly headache episodes (Weeks 1-12, 13-24, 25-36, 37-48, 49-60)
- Change from Baseline in monthly days with use of acute migraine medication (Weeks 1-4, 5-8, 9-12, 13-16, 17-20, 21-24, 25-28, 29-32, 33-36, 37-40, 41-44, 45-48, 49-52, 53-56, 57-60)

For the endpoints listed above, the Baseline MMDs will be replaced with the Baseline score for the endpoint in question in the model, except for the analyses of PGIC and MBS, which will exclude Baseline score terms, since the rating of the scales in themselves represent a change from Baseline so no Baseline score exists. Furthermore, month will be replaced with the relevant visit structure for the endpoint in question.

For endpoints regarding 12-week intervals (Weeks 1-12, Weeks 13-24, etc.), the estimates will come from models based on 4-week intervals (Weeks 1-4, Weeks 5-8, etc.) as described in Section 12.5.1.2.

12.5.3.2 Effectiveness Endpoints Regarding Response

For endpoints regarding response (50% and 75%) the number and percentages of responders will be summarized by 4-weeks intervals (Weeks 1-4, Weeks 5-8, Weeks 9-12, Weeks 13-16, Weeks 17-20, Weeks 21-24, Weeks 25-28, Weeks 29-32, Weeks 33-36, Weeks 37-40, Weeks 41-44, Weeks 45-48, Weeks 49-52, Weeks 53-56, Weeks 57-60) and by 12-weeks intervals (Weeks 1-12, Weeks 13-24, Weeks 25-36, Weeks 37-48, Weeks 49-60) using the imputation rules described in Section 22.1.1.2.

In addition to the descriptive statistic, confidence intervals based on normality assumptions will be presented.

12.5.3.3 Other Effectiveness Endpoints

Endpoints that are not covered by Section 12.5.3.1 and Section 12.5.3.2 will only be summarized descriptively as described in Chapter 6.

12.5.3.4 Subgroup Analyses

Analyses of the following endpoints will be repeated for the subgroup of participants in “EPTI 300 mg at least once” as defined in Section 4.5.

- Change from Baseline in the number of MMDs (Weeks 1-4, 5-8, 9-12, 13-16, 17-20, 21-24, 25-28, 29-32, 33-36, 37-40, 41-44, 45-48, 49-52, 53-56, 57-60)
- Change from Baseline in the number of MMDs (Weeks 1-12, 13-24, 25-36, 37-48, 49-60)

12.5.4 Rationale for Selected Analysis Method for the Secondary and Exploratory Endpoints

The Change from Baseline in MMDs are generally considered continuous data, and they are analyzed using methods based on observations following a normal distribution. Given the repeated observation of normally distributed data, an MMRM analysis using all available data has been chosen for the analyses. Covariates are included in the model based on an approach including key factors representing trial design features (visit), and baseline number of MMDs to account for differences in baseline MMDs and its predictive ability. When the MMRM analysis includes the individual factors mentioned as well as interaction of visit and baseline score, and applies an unstructured covariance, as described in Section 12.5.1.2, it allows for flexibility in modelling the development over time and similarly provides robust estimation, even under some deviation from the assumption of normality.

The same rationale applies to the additional endpoints described in Section 12.5.3.1.

12.5.5 Sensitivity Analyses of Secondary Endpoints

12.5.5.1 Sensitivity Analyses to assess the impact of missing data regarding MMDs

A sensitivity analysis using multiple imputation (MI) method to assess the robustness of the conclusions for change from Baseline in MMDs with respect to the missing data will be conducted. Specifically, an analysis will be performed using a pattern-mixture model (PMM), in which the remaining missing data after application of the prorating rule, see Section 22.1.1.1, will be imputed using a sequential regression-based multiple imputation method.[2]

200 simulations will be performed to generate the datasets that will be analysed using the model described in Section 12.5.1.2. These analyses will be combined using Rubin's rule to form a unique point estimate and standard error, taking into account the uncertainty of the imputation.[3] The SAS® code for this sensitivity analysis is shown in Appendix III.

12.5.5.2 Sensitivity Analysis to assess the impact of missing data regarding 50% response on MMDs

For the secondary endpoint, response defined as ≥50% reduction from Baseline in MMDs (Weeks 1-12, 13-24,…, 49-60), a sensitivity analysis will be performed, missing data will be filled in using the MI methodology described in Section 12.5.5.1, whereafter, following the approach by Ratitch et al,[4] the results will be combined across the 200 data sets, using PROC MIANALYZE to generate a combined set of estimates.

12.5.6 Selected Analyses by Prior Treatment Group

The following endpoints will, in addition to the analyses described in Section 12.5.3 be analysed by Prior Treatment Group:

- Change from Baseline in the number of MMDs (Lead-in Weeks 1-4, 5-8, 9-12; Weeks 1-4, 5-8, 9-12, 13-16, 17-20, 21-24, 25-28, 29-32, 33-36, 37-40, 41-44, 45-48, 49-52, 53-56, 57-60)
- Change from Baseline in the number of MMDs (Lead-in Weeks 1-12; Weeks 1-12, 13-24, 25-36, 37-48, 49-60)
- Change from Baseline in the number of MHDs (Lead-in Weeks 1-4, 5-8, 9-12; Weeks 1-4, 5-8, 9-12, 13-16, 17-20, 21-24, 25-28, 29-32, 33-36, 37-40, 41-44, 45-48, 49-52, 53-56, 57-60)
- Change from Baseline in the number of MHDs (Lead-in Weeks 1-12; Weeks 1-12, 13-24, 25-36, 37-48, 49-60)

The endpoints will be analysed using the same methods as described in Section 12.5.3.1 with the following changes:

Analyses based on MMRM will in addition to the fixed effects described in Section 12.5.1.2 also contain the following fixed effects: Prior treatment group, Prior treatment group-by-month interaction.

In addition the 50% and 75% responders described in Section 12.5.3.2 will be presented descriptively by prior treatment group.

13 Safety Estimand

General handling of safety endpoints will be addressed in Chapter 14.

13.1 Estimand for the safety endpoints

Summary of the intercurrent events that will be addressed and the estimand attributes can be found below. Further details on the intercurrent events and the strategies used to address them are given in Section 13.2.

The safety estimand will be the proportion of patients experiencing at least one TEAE among patients with chronic migraine who have been treated with eptinezumab 100 mg/300 mg every 12 weeks for 60 weeks, with or without the use of other preventive treatments, and regardless of infusion interruption due to other reasons than TEAE and prior to withdrawal from trial for other reasons than TEAE.

Clinical question of interest:

The clinical question of interest is the proportion of patients experiencing at least one TEAE during 60 weeks of treatment with eptinezumab.

Attributes to the safety estimand

The safety estimand is described by the following attributes:

- The **treatment condition of interest** is treatment with eptinezumab 100 mg/300 mg every 12 weeks for 60 weeks, with or without use of other preventive migraine medications
- The **population of interest** is patients with chronic migraine
- The **endpoint to be considered** is to experience at least one TEAE during 60 weeks of treatment with eptinezumab
- The **intercurrent events** will be:
  - infusion interruption due to other reasons than TEAE which will be addressed using a treatment policy strategy
  - withdrawal from trial due to other reasons than TEAE which will be handled using a while on treatment strategy
- The **population level summary** will be the proportion of patients experiencing at least one TEAE during 60 weeks of treatment with eptinezumab

13.2 Rationale for the safety estimand

Endpoint

The proportion of participants experiencing at least one TEAE during 60 weeks of treatment with eptinezumab is considered a good measure of the overall safety of the drug, since it reflects the safety of the drug used for a long period reflecting how the drug would be used by patients suffering for chronic migraine. Therefore, the proportion of participants experiencing at least one TEAE during treatment with eptinezumab every 12 weeks for 60 weeks will be used as the endpoint for the estimand.

Treatment Conditions of Interest

The use of preventive medication is included in the treatment condition of interest because eptinezumab is expected to be used with or without other preventive medications by patients suffering from chronic migraine. Furthermore, though the use of preventive medication could influence the number of TEAEs that the patient experience, it will reflect the general safety the patients will experience when using the drug.

Intercurrent events:

The following intercurrent events, occurring after treatment initiation and potentially affecting either the interpretation or the existence of the measurements associated with the clinical question of interest, are considered:

- Infusion interruption:
  If an IMP is stopped prematurely (either temporarily or completely) due to other reasons than TEAE, the consequence is that the participant did not receive all of the intended dose of eptinezumab and potentially this would decrease the risk of experiencing an AE afterwards. A treatment policy strategy is used to address this since we want to include all TEAEs also observed after infusion interruption.
- Withdrawal from trial:
  If a participant withdraws from trial due to other reasons than TEAE, the consequence will be that safety data will not be collected after the Safety Follow-up Visit. The intercurrent event will be handled using a while on treatment strategy.

13.3 Estimator

The proportion of participants experiencing at least one TEAE will be presented descriptively. In addition to the descriptive statistic, a confidence interval based on normality assumptions will be presented.

14 Safety

14.1 Adverse Events

14.1.1 General Methodology for Adverse Events

Unless otherwise specified, tables, graphs, and listings will be based on the APTS.

All the tables and graphs will be presented for all participants, unless otherwise specified.

Tables by preferred term and tables by system organ class (SOC) and preferred term will be sorted in descending order based on the percentages of participants with these adverse events.

Unless otherwise specified, the summaries of adverse events will include the number and percentage of participants with an adverse event.

Listings of adverse events will be sorted by site, participant screening number, and adverse event start date, and include preferred term, investigator term, adverse event start date, the day of first IMP infusion, the date and time of the latest IMP infusion prior to the adverse event, the time since latest IMP infusion, duration of the adverse event, action taken, causality, intensity, seriousness, and outcome. For adverse events that change in intensity, each intensity will be included. In listings of adverse events, dates will be displayed as collected also in case of partially or completely missing dates.

14.1.2 Coding of Adverse Events

Adverse events will be coded using MedDRA, Version 27.0 or later.

14.1.3 Classification of Adverse Events

Adverse events will be classified according to the time of onset of the adverse event:

- *treatment-emergent adverse event* (TEAE) – an adverse event that either starts, increases in intensity or changes from non-serious to serious on or after the date and time of first dose of IMP in trial 19140B

For handling of adverse events with incomplete start dates to facilitate this classification, see Section 22.3.5.

An adverse event is considered causally related to the use of the IMP when the causality assessment by the investigator is *probable* or *possible*. If the causality assessment is missing, the adverse event is considered causally related.

14.1.4 Presentation of Adverse Events

All adverse events will be listed based on APES, including a flag for TEAEs.

An overview of the numbers and percentages of participants with TEAEs, serious adverse events (SAEs), adverse events leading to withdrawal from trial, or adverse events leading to infusion interruption, and of participants who died will be provided.

14.1.5 Presentation of Adverse Events Starting Prior to First Infusion in Trial 19140B

Adverse events starting after informed consent for Trial 19140B is signed but before first infusion in Trial 19140B is received, will be listed based on APES.

14.1.6 Presentation of Treatment-emergent Adverse Events

The following summaries will be provided:
- TEAEs by SOC and preferred term
- TEAEs by preferred term
- TEAEs by sex and preferred term
- TEAEs with an incidence ≥2 by preferred term
- causally related TEAEs by SOC and preferred term
- TEAEs by intensity (mild/moderate/severe), SOC, and preferred term
- causally related TEAEs by intensity, SOC, and preferred term
- TEAEs occurring on the day of dosing after infusion start in Trial 19140B (i.e. at Week 0, Week 12, Week 24, Week 36 and Week 48 visits) by SOC and preferred term
- TEAEs by preferred term for the subgroup of participants that at any time during Trial 19140B have received eptinezumab 300 mg.
- TEAEs by preferred term for the subgroup of participants that throughout Trial 19140B have received eptinezumab 100 mg.

For TEAEs occurring on the day of dosing after infusion start, TEAEs with missing start times will also be included.

14.1.7 Presentation of Deaths

All the adverse events in participants who died will be listed.

14.1.8 Presentation of Serious Adverse Events

All SAEs will be listed.

Treatment-emergent SAEs will be summarized by:
- SOC and preferred term
- preferred term

14.1.9 Presentation of Adverse Events Leading to Withdrawal from Trial

All AEs leading to withdrawal from trial will be listed.

TEAEs leading to withdrawal from trial will be summarized by:
- SOC and preferred term
- preferred term

14.1.10 Presentation of Adverse Events Leading to Infusion Interruption or Termination

All AEs leading to infusion interruption or termination will be listed.

TEAEs leading to infusion interruption or termination will be summarized by:
- SOC and preferred term
- preferred term

14.1.11 Presentation of Adverse Events of Special Interest

Adverse events of special interest (AESI) will consist of the preferred terms defined by SMQs/HLTs/HLGTs listed in Table 5.

All AESIs will be listed.

The listing will be repeated for all AESIs belonging to the Hypersensitivity and Anaphylactic Reactions event category only, see Table 5. Previous treatment group from Trial 19140A will be included in the listing.

The following summaries of treatment-emergent AESIs will be provided:
- AESIs by SOC and preferred term
- AESIs by SOC and preferred term, separately for each individual event category
- AESIs belonging to the Hypersensitivity and Anaphylactic Reactions event category by Previous Treatment group, SOC, and preferred term.

14.2 General Methodology for Other Safety Data

Unless otherwise specified, tables, graphs, and listings will be based on the APTS.

The denominators for the summaries of a given variable will be based on the number of participants with non-missing values at a given visit or during the assessment period.

Descriptive statistics for the safety variables, both absolute values and changes from Baseline, will be presented by visit.

The number and percentage of participants with at least one PCS value at any post-baseline assessment time point will be summarized by variable. All available assessments will be included in the evaluation of PCS values.

For participants with post-baseline PCS values, listings will be provided including all the values of the variable for those participants, with flagging of PCS values and out-of-reference-range values.

All the adverse events in participants with post-baseline PCS values will be listed by previous treatment group and participant screening number. The listing will include the PCS value, the assessment date, the change from Baseline in PCS value, the preferred term for the adverse event, and start date and stop date of the adverse event. The PCS values and adverse events will be listed in chronological order according to assessment date and the start date of the adverse event.

For urine dipsticks, for which the results are categorical values (for example, negative, trace, 1+, 2+), the number of participants will be summarized by visit for each test. The microscopy results will be listed by assessment time point for participants with findings.

14.3 Clinical Safety Laboratory Test Data

14.3.1 Data Presentation

The PCS criteria for the clinical safety laboratory tests are in Table 2 in Appendix IV.

The clinical safety laboratory test values will be presented both in conventional and Système International (SI) units.

Fasting lipid and fasting glucose concentrations will be presented in separate tables from the overall laboratory tables.

14.3.2 Potential Drug-induced Liver Injury (DILI)

Signals of DILI will be assessed according to the FDA guideline[5] using the following criteria:

- ALT or AST >2×, >3×, >5×, >10×, or >20×ULN
- total bilirubin >2×ULN
- alkaline phosphatase >1.5×ULN
- ALT or AST >3×ULN AND total bilirubin >1.5× or >2×ULN

Participants fulfilling any of the criteria will be listed, and the listing will include all the ALT, AST, bilirubin, and alkaline phosphatase values for those participants, sorted by assessment date and time in ascending order. If a criterion for a test is fulfilled, the value will be flagged with the highest criterion fulfilled (for example, AST >3×ULN, >5×ULN, >10×ULN, or >20×ULN).

In addition, assessment time points for participants for whom Hy's Law is potentially fulfilled will also be flagged in the listing:

- ALT or AST >3×ULN AND
- alkaline phosphatase <2×ULN AND
- total bilirubin ≥2×ULN

The number of participants who met any of the criteria specified above at any post-baseline visit will be summarized. In the summary, each participant will be counted only once using the maximum assessment, or the most severe for the combined criteria. The summary will also include the number of potential Hy's Law cases. If any participant fulfil Hy's Law (all 3 criteria fulfilled), information collected related to this finding will be presented in a listing.

14.4 Vital Signs and Weight

The PCS criteria used for vital signs and weight are included in Table 3 in Appendix IV.

14.5 ECGs

The PCS criteria used for the ECG parameters are included in Table 4 in Appendix IV.

In addition to the tables and listings specified in Section 14.2, absolute values and changes from Baseline in QTcF will also be summarized categorically by visit. The categories that will be used are as follows for the absolute QTcF values:

- QTcF interval < 450 msec
- QTcF interval 450 - 480 msec
- QTcF interval > 480 - 500 msec
- QTcF interval > 500 msec

The categories that will be used for the change from Baseline QTcF values are:

- QTcF interval increase from Baseline > 30 msec
- QTcF interval increase from Baseline > 60 msec

Furthermore, the number and percentage of participants being classified as having either a normal, abnormal but not clinically significant, abnormal and clinically significant ECG result based on the overall interpretation of the ECG from the investigator will be summarized by visit.

14.6 Other Safety Endpoints

14.6.1 Columbia-Suicide Severity Rating Scale (C-SSRS) Scores

The C-SSRS was administered after baseline (post-baseline) using the *Since Last Visit Version*.

The numbers and percentages of participants with post-baseline suicide-related events based on the C-SSRS will be summarized. The most severe item with an answer "Yes" for each participant according to the ordering in Panel 3 is displayed.

The number and percentage of participants with *no suicidal ideation or behaviour* will be included in the summary.

Panel 3 C-SSRS Scores

C-SSRS Score		Related to:
1	Wish to be dead	Suicidal ideation
2	Non-specific active suicidal thoughts	
3	Active suicidal ideation with any methods (not plan) without intent to act	
4	Active suicidal ideation with some intent to act, without specific plan	
5	Active suicidal ideation with specific plan and intent	
6	Preparatory acts or behaviour	Suicidal behaviour
7	Aborted attempt	
8	Interrupted attempt	
9	Non-fatal suicide attempt	
10	Completed suicide (only applicable for the post-baseline assessments)	

Missing C-SSRS scores will not be imputed.

Positive responses to *non-suicidal self-injurious behaviour* will be summarized separately.

A listing will be provided for participants with any post-baseline suicidal ideation or behaviour including all the C-SSRS scores for those participants. C-SSRS scores related to suicidal behaviour will be flagged.

15 Immunogenicity

The following definitions will be used in this section:

- *ADA incidence*: The proportion of participants with treatment-induced ADAs (seroconverted) and treatment-boosted ADAs during the trial
- *ADA-positive participant*: A participant with ≥1 treatment-induced or treatment-boosted ADA positive sample after administration of a therapeutic protein at any time during the treatment or follow-up observation period

- *ADA-positive sample*: A sample is considered positive when ADAs are detected
- *ADA prevalence*: The proportion of participants with ADAs (including pre existing ADAs) at any point in time
- *Persistent ADA*: Treatment-induced ADAs detected at ≥2 sampling time points during the treatment period (including the follow-up observation period if any), where the first and last ADA-positive samples (irrespective of any negative samples in between) are separated by ≥16 weeks OR treatment-induced ADAs detected only at the last sampling time point of the treatment period or at a sampling time point <16 weeks before an ADA-negative last sample. If the last sampling time-point is the only ADA-positive sample, it is referred to as a persistent ADA response.
- *Pre-existing ADA*: Pre-existing ADAs reactive with the administered therapeutic protein, that is, ADAs that are present in the participant prior to the first administration of the therapeutic protein.
- *Transient ADA*: Treatment-induced ADAs detected only at 1 sampling timepoint during the treatment or follow-up observation period (excluding the last sampling time point, which ought to be considered persistent unless shown to be undetectable at a later time), OR treatment-induced ADAs detected at ≥2 sampling time points during the treatment period (including the follow-up observation period, if any), where the first and last ADA-positive samples (irrespective of any negative samples in between) are separated by <16 weeks, and the participant's last sampling time point is ADA negative.
- *Treatment-boosted ADA*: Pre-existing ADAs that are boosted to a higher level after administration of a therapeutic protein. "Boosted to a higher level" is defined as an ADA titre greater than the Baseline titre by a ≥2.34 increase.
- *Treatment-induced ADA*: ADAs that develop de novo after administration of a therapeutic protein

An overview of the numbers and percentages of participants with ADA results, pre-existing ADA, ADA prevalence, ADA incidence, treatment-induced ADA (in total, transient ADA, persistent ADA), and treatment-boosted ADA in total (transient ADA, persistent ADA) will be provided based on the APTS and will include results for both 19140A and 19140B.

The number and percent of participants with ADA-positive samples during the trial 19140A and 19140B combined will be summarized by visit. Denominators for percentages will be the total number of samples taken for the specified visit. The summaries will also include the total number of ADA-positive samples and the number and percentage of ADA-positive participants anytime during the trial. The denominator for percentage of ADA-positive participants will be the total number of participants contributing with a sample anytime after first IMP with eptinezumab in trial 19140A.

For ADA-positive samples, the titres and the NAb results will be summarized by visit and overall.

All the immunogenicity data, including assessment date, ADA result, titre result, and NAb result, will be listed for participants with at least one positive anti-eptinezumab antibody result in 19140A or 19140B including the baseline. The listing will include Previous Treatment Group.

A summary of TEAEs by SOC and preferred term will be provided for participants who are ADA-positive and for participants who are ADA-negative. In addition, AESIs belonging to the Hypersensitivity and Anaphylactic Reactions event category will be summarized by Previous Treatment group, SOC, and preferred term for participants who are ADA-positive and for participants who are ADA-negative.

AESIs of Hypersensitivity and Anaphylactic Reactions in participants with positive ADA results in 19140B including the baseline, will be listed. The listing will include the ADA results from both trial 19140A and trial 19140B, the ADA sampling dates, the preferred term for the adverse event, TEAE seriousness, the start date and stop date of the adverse event, and time since first and last infusion. The ADA results and adverse events will be listed in chronological order according to ADA sampling date and the start date of the adverse event.

The number and percentages of participants achieving at least 50% reduction in the number of MMDs compared to Baseline of Trials 19140A will be presented by 4-week intervals for participants who are ADA-positive and for participants who are ADA-negative. The summary will also be provided for participants who have a positive NAb result.

16 Pharmacokinetic/Pharmacodynamic Analyses

A separate analysis plan for pharmacokinetic/pharmacodynamic analyses will be prepared by PK/PD Modelling & Simulation, H. Lundbeck A/S. The PK/PD analysis plan will be finalized before unblinding/data delivery of plasma concentrations.

17 Data Reviews

The quality of the trial will be overseen by performing data reviews during the conduct of the trial. The reviews may include, but are not limited to, data quality, protocol adherence, and the appropriateness of design assumptions, including the sample size assumptions.

18 Interim Analyses

No interim analysis is planned.

19 Sample Size Considerations

No formal sample size calculations have been performed. The trial is planned to enrol participants in Japan who have completed the Lead-in Trial 19140A and fulfilled the entry criteria for the Open-label Trial 19140B. Approximately 154 participants, recruited from specialist settings, are planned for enrolment in the Lead-in Trial in Japan with opportunity to enrol in Trial 19140B with the aim to have 100 participants complete Trial 19140B.

The targeted number of participants necessary for evaluating 12 months exposure safety in Trial 19140B in Japan was achieved under Trial 19140A Protocol edition 2.0. Therefore, participants in Japan recruited in Trial 19140A under Protocol edition 3.0 will not enter Trial 19140B, but will instead receive active treatment during the Extension Period of Trial 19140A.

20 Statistical Software

The statistical software used will be SAS®, Version 9.4 or later.

21 Changes to Analyses Specified in the Protocol

The endpoint Change from Baseline in HCRU (at Weeks 4, 8, 12, 16, 20, 24, 28, 32, 36, 40, 44, 48, 52, 56, 60) will be changed to the following endpoint:

- HCRU (at Baseline and Weeks 4, 8, 12, 16, 20, 24, 28, 32, 36, 40, 44, 48, 52, 56, 60)

The definition the analysis set FAS has been redefined in Chapter 5 to contain participants with a valid baseline assessment of MMD based on eDiary data from the first 28 days of the Screening Period of Trial 19140A. The change has been made to align with effectiveness analyses on MMDs which will be based on the Baseline value captured in 19140A.

In the Protocol Section 16.8.1 it was stated that the Baseline Visit of the Trial 19140B should be used for effectiveness analyses. The Baseline of Trial 19140A will be used for effectiveness analyses instead to have a baseline from before the participants receive any infusion.

In the protocol all analyses and presentation of data will be done overall and by prior treatment. This is changed so that only specific summaries and analyses are presented by prior treatment.

The following exploratory endpoints have been added for completeness:

- Response: ≥75% reduction from Baseline in MMDs (Weeks 1-4, 5-8, 9-12, 13-16, 17-20, 21-24, 25-28, 29-32, 33-36, 37-40, 41-44, 45-48, 49-52, 53-56, 57-60)
- Response: ≥75% reduction from Baseline in MMDs (Weeks 1-12, 13-24, 25-36, 37-48, 49-60)
- Change from Baseline in the number of MHD's (Weeks 1-4, 5-8, 9-12, 13-16, 17-20, 21-24, 25-28, 29-32, 33-36, 37-40, 41-44, 45-48, 49-52, 53-56, 57-60)
- Change from Baseline in the number of MHD's (Weeks 1-12, 13-24, 25-36, 37-48, 49-60)
- Response: ≥50% reduction from Baseline in MHDs (Weeks 1-4, 5-8, 9-12, 13-16, 17-20, 21-24, 25-28, 29-32, 33-36, 37-40, 41-44, 45-48, 49-52, 53-56, 57-60)
- Response: ≥50% reduction from Baseline in MHDs (Weeks 1-12, 13-24, 25-36, 37-48, 49-60)

- Response: ≥75% reduction from Baseline in MHDs (Weeks 1-4, 5-8, 9-12, 13-16, 17-20, 21-24, 25-28, 29-32, 33-36, 37-40, 41-44, 45-48, 49-52, 53-56, 57-60)
- Response: ≥75% reduction from Baseline in MHDs (Weeks 1-12, 13-24, 25-36, 37-48, 49-60)
- Change from Baseline in the proportion of migraine attacks with severe pain intensity (Weeks 1-12, 13-24, 25-36, 37-48, 49-60)
- Change from Baseline in the proportion of headache episodes with severe pain intensity (Weeks 1-12, 13-24, 25-36, 37-48, 49-60)
- Change from Baseline in monthly migraine attacks (Weeks 1-12, 13-24, 25-36, 37-48, 49-60)
- Change from Baseline in monthly headache episodes (Weeks 1-12, 13-24, 25-36, 37-48, 49-60)
- Change from Baseline in monthly days with use of acute migraine medication (Weeks 1-4, 5-8, 9-12, 13-16, 17-20, 21-24, 25-28, 29-32, 33-36, 37-40, 41-44, 45-48, 49-52, 53-56, 57-60)

22 Details on Data Handling

22.1 Derived Variables

22.1.1 eDiary

22.1.1.1 Monthly Migraine Days (MMDs)/Monthly Headache Days (MHDs)

The following describes the derivation of MMDs. The derivation of MHDs follows the same principles.

4-Week Intervals

For Baseline and each 4-week period post-baseline in the trial, the MMDs will be derived as the number of migraine days within each 28-day interval using the imputation rules described below. The 4-week periods post-baseline that are considered are the following: Weeks 1-4, Weeks 5-8, Weeks 9-12, Weeks 13-16, Weeks 17-20, Weeks 21-24, Weeks 25-28, Weeks 29-32, Weeks 33-36, Weeks 37-40, Weeks 41-44, Weeks 45-48, Weeks 49-52, Weeks 53-56, Weeks 57-60.

In general, for 4-week periods where the eDiary is completed on at least 14 days out of the 28 days, prorating will be used to calculate the MMDs, and for 4-week periods where the eDiary is completed on less than 14 days out of the 28 days, the MMDs will be set to missing. After this calculation procedure has been applied, a non-missing 4-week MMD value is considered a valid assessment, see terminology used in Chapter 5 above.

Prorating

Intended to be used for 4-week periods where the eDiary is completed on at least 14 out of the 28 days, the prorating procedure consists of imputing days with missing information with the observed mean number of migraine days in the period as follows:

28(Reported Migraine Days/Reported eDiary Days)*

This imputation rule will also be used for the Baseline value regardless of the number of reported eDiary days in the screening period of Trial 19140A.

Calculation of MMDs in Case of Intercurrent Events

In case of the intercurrent event Use of other long-acting anti-CGRPs than eptinezumab, eDiary data are excluded 28 days after the recorded used other long-acting anti-CGRPs. After removing these data, MMDs are calculated and prorated as described above.

22.1.1.2 Migraine/Headache Responder Variables

The following describes the derivation for response for monthly migraine days. The derivation for monthly headache days is similar.

The following response variables will be derived: 50% and 75%. A responder is a participant, who achieves a ≥50% reduction or ≥75% reduction in MMDs, respectively, compared to the Baseline MMDs. The derivation of these responder endpoints will be based on the MMDs resulting from the imputations described in Section 22.1.1.1.

For each 4-week period post-baseline in the trial, the responder status of a participant will be derived based on the percentage change from Baseline in MMDs. If the MMDs value is missing for the month in question, the response status will also be missing.

For the 12-week interval, the 50% and 75% response status will be derived as follows, using 50% response for Weeks 1-12 as an example:

$$\text{50\% Response Status (Weeks } 1-12) = \begin{cases} 1, & \text{if } \dfrac{\text{ave}(\Delta_{M1}, \Delta_{M2}, \Delta_{M3})}{Baseline} \leq -0.5 \\ 0, & \text{if } \dfrac{\text{ave}(\Delta_{M1}, \Delta_{M2}, \Delta_{M3})}{Baseline} > -0.5, \end{cases}$$

where Δ_{Mi} is the change from Baseline value for month i. If any of the months included in the calculation have a missing value of MMDs, the responder status will be derived based on the available values.

22.1.1.3 Migraines/Headaches With Severe Pain Intensity and Headache Episodes/Migraine Attacks

The proportion of migraines and headaches with severe pain intensity is derived based on the derived monthly migraine attacks and headache episodes.

A migraine attack is defined as 1 continuously recorded migraine. One attack may result in multiple migraine days. Headache episodes are similarly defined. Headache severity is collected on a 3-point scale, Mild, Moderate and Severe. Migraines and headaches with severe pain intensity are defined as migraine attacks/headache episodes with a reported severity of "Severe".

The following describes the derivation of monthly migraine attacks. The derivation of monthly headache episodes follows the same principles.

The derivation of monthly migraine attacks will follow the imputation rules described in Section 22.1.1.1 for MMDs, replacing *Reported Migraine Days* with *Reported Migraine Attacks* everywhere. As an example, monthly migraine attacks for a participant with 14 days or more of eDiary reporting in a 4-week period will be derived as:

28(Reported Migraine Attacks/Reported eDiary Days)*

Monthly migraines and headaches with severe pain intensity is derived in the same way using only migraine attacks/headache episodes with a reported severity of "Severe".

The proportion of migraines and headaches with severe pain intensity is then calculated as the proportion of migraine attacks/headache episodes with severe pain intensity out of the number of migraine attacks/headache episodes. Participants with no migraine attacks/headache episodes are included with a rate of 0.

22.1.1.4 Monthly Days with Acute Migraine Medication Usage and MMDs/MHDs with Acute Medication Usage

In the evening eDiary, participants are asked each day to fill out whether they used any of the following medications during that day: Ergotamine, Triptan, Analgesic, Opioid, Combination Analgesic, TCMs (Traditional Chinese Medicines), Antiemetics (metoclopramide, domperidone), Analginum, Antipyrine, Tolfenamic acid. A day where the participant answers that they took any of those in the evening eDiary is considered a day with use of acute migraine medication.

The number of days with acute migraine medication usage is then derived for each 4-week period using the imputations described in Section 22.1.1.1 and replacing *Reported Migraine Days* with *Reported Acute Migraine Medication Days* everywhere as well as replacing *Reported eDiary Days* with *Reported Evening eDiary Days*, where a "*Reported Evening eDiary Day*" is defined as any day where the evening eDiary has been filled out.

Similarly, the number of days with each of the medication types taken will be derived as well as the number of days with any medication use.

Additionally, the monthly migraine days/monthly headache days with acute medication usage will be derived. This is derived using the answer to "*Did you take any medications to treat this headache?*" in the headache diary. The question is asked when a participant is ending a headache. Thus, a migraine/headache day with acute medication usage is defined as a migraine/headache day with the extra condition that this question has been answered as "Yes". In case of a migraine/headache with acute medication usage spanning multiple days, all the days will be counted as a migraine/headache day with use of acute medication.

22.1.2 Patient Global Impression of Change (PGIC)

The PGIC is a single participant-reported item reflecting the participant's impression of change in their disease status since the start of the trial (that is, in relation to activity limitations, symptoms, emotions, and overall quality of life).

The item is rated on a 7-point scale ranging from 1 (very much improved) to 7 (very much worse).

22.1.3 Most Bothersome Symptom (MBS)

The Investigator will verbally obtain the most bothersome symptom associated with the participant's migraines during the Screening Visit of the Lead-in Trial. Participants will be asked to rate the improvement in this symptom from screening on a 7-point scale identical to the scale used for the PGIC, i.e. the scale ranges from 1 (very much improved) to 7 (very much worse).

The MBS areas include: nausea, vomiting, sensitivity to light, sensitivity to sound, mental cloudiness, fatigue, pain with activity, mood changes, and other.

22.1.4 Headache Impact Test (HIT-6)

The HIT-6 (v1.0)[6] is a Likert-type, self-reporting questionnaire designed to assess the impact of an occurring headache and its effect on the ability to function normally in daily life. The HIT-6 contains 6 questions, each item is rated from "never" to "always" with the following response scores: never = 6, rarely = 8, sometimes = 10, very often = 11, and always = 13. The total score for the HIT-6 is the sum of each response score and ranges from 36 to 78. The life impact derived from the total score is described as followed: Severe (60-78), Substantial (56-59), Some (50-55), Little to None (36-49).

22.1.5 Migraine-Specific Quality of Life Questionnaire Version 2.1 (MSQ v2.1)

The MSQ v2.1[7] is a participant-reported outcome designed to assess the quality of life in patients with migraine. It consists of 14 items covering three domains: role function restrictive

(7 items); role function preventive (4 items); and emotional function (3 items). Each item is scored on a 6-point scale ranging from 1 (none of the time) to 6 (all of the time).

The items going into each domain[8] are specified in Panel 4.

Each item score is mapped from the recorded value of the item as shown in Panel 5.[9]

For each domain, the score is derived from the final item values by summing the scores from items within each domain and transforming the summed scores as shown in Panel 6.

Panel 4 MSQ v.2.1 Domains

Domain	Item number	Abbreviated content
Role function restrictive	1	. . . . . interfered with how well you dealt with family, friends, and others
	2	. . . . . interfered with your leisure time activities such as reading or exercising
	3	. . . . . had difficulty in performing work or daily activities
	4	. . . . . kept you from getting as much done at work or at home
	5	. . . . . limited your ability to concentrate on work or daily activities
	6	. . . . . left you too tired to do work or daily activities
	7	. . . . . limited the number of days you felt energetic
Role function preventive	8	. . . . . canceled work or daily activities . . .
	9	. . . . . needed help in handling routine tasks
	10	. . . . . stopped work or daily activities . . .
	11	. . . . . not able to go to social activities . . .
Emotional function	12	. . . . .felt fed up or frustrated
	13	. . . . . felt like a burden on others
	14	. . . . . afraid of letting others down

Panel 5 MSQ v.2.1 Item Values

Response categories	Precoded item value	Final item value
None of the time	1	6
A little bit of the time	2	5
Some of the time	3	4
A good bit of the time	4	3
Most of the time	5	2
All of the time	6	1

Panel 6 Derivation of MSQ v.2.1 Domain Scores

Domain	Sum of item scores range	Derivation
Role function restrictive	7 to 42	(Summed score – 7)*100/35
Role function preventive	4 to 24	(Summed score – 4)*100/20
Emotional function	3 to 18	(Summed score – 3)*100/15

22.1.6 Euroqol 5 Dimension – 5 Levels (EQ-5D-5L)

The EQ-5D-5L[10] is a participant-reported assessment designed to measure the participant's well-being. It consists of 5 descriptive items (mobility, self-care, usual activities, pain/discomfort, and depression/anxiety) and a visual analogue scale (VAS) of the overall health state. Each descriptive item is rated on a 5-point index ranging from 1 (no problems) to 5 (extreme problems) and a single summary index (from 0 to 1) can be calculated.

The VAS is scored separately and ranges from 0 (worst imaginable health state) to 100 (best imaginable health state).

22.1.7 Health Care Resource Utilization (HCRU)

Migraine-specific healthcare resource utilization information will be collected in terms of outpatient health care professional visits (number of visits to doctor/genral practitioner and number of visits to a specialist), number of emergency room visits, number of hospital admissions, as well as number of overnight hospital stays during the past 4 weeks.

The items from the questionnaire will be analyzed separately.

22.1.8 Work Productivity and Activity Impairment: Migraine (WPAI:M)

The WPAI[11] is a participant self-rated scale designed to provide a quantitative measure of the work productivity and activity impairment due to a specific health problem (WPAI:M). The WPAI assesses activities over the preceding 7 days and consists of 6 items: 3 items assess the number of hours worked, the number of hours missed from work due to the participant's condition, or due to other reasons, and 2 visual numerical scales to assess how much the participant's condition affects their productivity at work and their ability to complete normal daily activities.

The derivation of the WPAI:M sub-scores[12] is given in Panel 7, where Q1-Q6 refers to question 1-6 in the questionnaire. If both Q2 and Q4 has a score of 0 then both the Absenteeism and the Work productivity loss sub-scores are set to missing.

Panel 7 Derivation of WPAI:M sub-scores

Sub-score	Description	Derivation
Absenteeism	Percent work time missed due to migraine	100*Q2/(Q2 + Q4)
Presenteeism	Percent impairment while working due to migraine	100*Q5/10
Work productivity loss	Percent overall work impairment due to migraine	$100 * \left(\frac{Q2}{Q2+Q4} + \left(1 - \frac{Q2}{Q2+Q4}\right) * \frac{Q5}{10}\right)$
Activity impairment	Percent activity impairment due to migraine	100*Q6/10

22.2 Assigning Data to Visits

See Section 4.1 for definition of Baseline values.

For all assessments the nominal visit will be used whenever it is available. In case a nominal visit is not available for unscheduled visits and withdrawal visits the assessments will be mapped to a visit as described in Section 22.2.1 and Section 22.2.2.

All available assessments will be used when reporting PCS values irrespectively of the assignment to visits.

22.2.1 Clinical Outcome Assessments (COAs) - Scales

For treated participants withdrawing, the withdrawal visit will also include effectiveness evaluations (PGIC, MBS, HIT-6, MSQ, EQ-5D-5L, HCRU, and WPAI:M). These assessments will be assigned to a nominal visit as specified in Panel 8 and Panel 9.

Panel 8 Visit Windows – PGIC, MBS, MSQ v2.1

Nominal Visit Number	Nominal Visit Week	Nominal Visit Day	Time Window
Visit 4	12	84	Day 1-148
Visit 7	24	168	Day 149-210
Visit 10	36	252	Day 211-294
Visit 13	48	336	Day 295-378
Visit 16	60	420	Day > 378

Panel 9 Visit Windows – HIT-6, EQ-5D-5L, HCRU, WPAI:M

Nominal Visit Number	Nominal Visit Week	Nominal Visit Day	Time Window
Visit 2	4	28	Day 1-42
Visit 3	8	56	Day 43-70
Visit 4	12	84	Day 71-98
Visit 5	16	112	Day 99-126

Nominal Visit Number	Nominal Visit Week	Nominal Visit Day	Time Window
Visit 6	20	140	Day 127-154
Visit 7	24	168	Day 155-182
Visit 8	28	196	Day 183-210
Visit 9	32	224	Day 211-238
Visit 10	36	252	Day 239-266
Visit 11	40	280	Day 267-294
Visit 12	44	308	Day 295-322
Visit 13	48	336	Day 323-350
Visit 14	52	364	Day 351-378
Visit 15	56	392	Day 379-406
Visit 16	60	420	Day > 406

Unscheduled visits will not contain assessments of the COAs mentioned above. In relation to the nominal visits post-baseline, a participant can complete their COAs 3 days before the visit. In case of duplicate scale assessments in relation to a nominal visit, i.e. if a participant fills out the scales 3 days before the visit and the visit is postponed afterwards implying that the participant has to complete the COA again, the latest assessment will be used for non-IMP visits and the latest assessment prior to or on the day of IMP at the visit will be used for IMP visits. In case of competing assessments at a visit after windowing, withdrawal visits will be prioritized over nominal visits.

22.2.2 Safety Variables

Laboratory Tests and ECG

Assessments of laboratory tests and ECGs at unscheduled visits and withdrawal visits will be assigned to a nominal visit according to the visit windowing specified in Panel 10 for participants in the APTS. Assessments for participants not receiving any infusions of IMP will be assigned to Visit 1 (Baseline Visit).

Panel 10 Visit Windows – Laboratory tests, ECG

Nominal Visit Number	Nominal Visit Week	Nominal Visit Day	Time Window
Visit 1 (Baseline Visit)	0	0	Same day as first IMP infusion (Day 0) and prior to IMP infusion on that date
Visit 4	12	84	After start of Visit 1 IMP infusion to start of Visit 4 IMP infusion
Visit 7	24	168	After start of Visit 4 IMP infusion to start of Visit 7 IMP infusion

Nominal Visit Number	Nominal Visit Week	Nominal Visit Day	Time Window
Visit 10	36	252	After start of Visit 7 IMP infusion to start of Visit 10 IMP infusion
Visit 13	48	336	After start of Visit 10 IMP infusion to start of Visit 13 IMP infusion
Visit 16	60	420	After start of Visit 13 IMP infusion

Vital signs

Vital signs will be assessed before and after infusion at nominal visits. For unscheduled or withdrawal visit assessments of vital signs, the values will be assigned a nominal visit according to Panel 11. Weight is only assessed one time at each visit. Windowing of that parameter will follow the windowing used for Laboratory tests and ECG.

Panel 11 Visit Windows – Vital Signs (Pulse rate, Blood Pressure, Temperature)

Nominal Visit Number	Nominal Visit Week	Nominal Visit Day	Time Window
Visit 1 (Baseline Visit) pre-dose	0	0	Same day as first IMP infusion (Day 0) and prior to IMP infusion on that date
Visit 1 (Baseline Visit) post-dose	0	0	After start of first IMP infusion to day 42
Visit 4 pre-dose	12	84	Day 43 to start of Visit 4 IMP infusion
Visit 4 post-dose	12	84	After start of Visit 4 IMP infusion to day 126
Visit 7 pre-dose	24	168	Day 127 to start of Visit 7 IMP infusion
Visit 7 post-dose	24	168	After start of Visit 7 IMP infusion to day 210
Visit 10 pre-dose	36	252	Day 211 to start of Visit 10 IMP infusion
Visit 10 post-dose	36	252	After start of Visit 10 IMP infusion to day 294
Visit 13 pre-dose	48	336	Day 295 to start of Visit 13 IMP infusion
Visit 13 post-dose	48	336	After start of Visit 13 IMP infusion to day 368
Visit 16	60	420	> 369

If there is more than one assessment at a visit (either due to multiple assessments or because an unscheduled or withdrawal visit assessment is mapped to a nominal visit with an already existing value) the value that will be used in summary tables by visit will be the one closest to

the nominal day for the visit but prioritizing values from scheduled visits above values from withdrawal or unscheduled visits. In the ordering of multiple values, assessments without recorded time will come after assessments with recorded time and the first in the ordering will be picked.

22.3 Handling Missing or Incomplete Dates/Times

22.3.1 Missing Headache Stop Date and Time

If the stop date and time for a headache recorded in the eDiary is missing, the headache stop date and time will be imputed as follows:

If there exists an entry in the evening eDiary at a date after the date of the start of the headache entered in the headache eDiary, the headache stop date and time is imputed with the date and time of the last evening eDiary entry.

If there are no entries in the evening eDiary after the date of the start of the headache entered in the headache eDiary, the headache stop date and time is imputed with the start date of the headache and the time 23:59.

Headaches that are not reported as stopped by the participant are missing the answers to the questions that allow for the determination of whether the headache was a migraine or not. Headaches with missing ‘end’ information will be classified as migraines (and thereby also as headaches).

Note that for each participant, it is only possible to have a missing stop date and time for the last headache ever entered, since a participant needs to complete a headache in order to report a new one in the headache eDiary.

22.3.2 Withdrawal Date

Missing withdrawal dates will not be imputed and time to withdrawal will not be calculated for missing withdrawal dates.

22.3.3 Medical Disorder Start and Stop Dates

Incomplete dates will not be imputed. Classification of events into *concurrent medical disorders* or *past disorders* will be based on the reported ongoing status.

22.3.4 Medication Start and Stop Dates

Imputation of incomplete or partially missing dates will be performed in order to document the assigned categories specified in Chapter 9.

The algorithm for imputing the start dates will follow the one used for imputing adverse event start dates, see Section 22.3.5.

For imputing stop dates, the following will apply, where UK and UKN indicate unknown or missing day and month, respectively:

- UK-MMM-YYYY: Medication stop date is imputed with the last day of the month
- UK-UKN-YYYY: Medication stop date is imputed with 31-DEC-YYYY

22.3.5 Adverse Event Start and Stop Dates

Imputation of partially or completely missing dates will be included in data in order to document the classification of the treatment emergent status and assignment of the adverse event to a period. For an adverse event with an imputed start date, the classification of treatment emergent will depend only on whether the imputed date is the same as the date(s) of infusion and not the timepoint of the infusion, since start times for adverse events will not be imputed. No duration will be calculated for adverse events with incomplete start-or-stop dates or for ongoing adverse events.

Imputation will follow the algorithm below. If an imputed start date after this procedure is after the stop date, the start date will be set to the stop date.

Start Dates

Participants with no IMP infusion

For participants who have not been treated in trial 19140B, the imputation of AE start date will be performed as follows, where UK and UKN indicate unknown or missing day and month, respectively:

- UK-MMM-YYYY: The start date will be imputed with either the 1st of the month, or date of the Baseline Visit. Date of the Baseline Visit will be used if that is the later of the two and if it is within the specified month and year
- UK-UKN-YYYY: The start date will be imputed with either JAN 1, or date of the Baseline Visit. Date of the Baseline Visit will be used if it is within the specified year

If the AE start date is completely missing, it will be set equal to the date of the Baseline Visit.

Participants who received at least one IMP infusion

For participants, who have been treated with at least one dose in trial 19140B, the imputation of AE start dates will be performed as follows:

- UK-MMM-YYYY:
  - If the year and month are equal to the year and month of treatment start date, the adverse event start date is imputed with the date of first dose of IMP in trial 19140B
  - If the year is equal to the year of treatment start date: If the month is prior to the treatment start date, the adverse event start date is imputed with the last day of the month. If the month is equal to the month of the treatment start date, see above. If the

month is after the month of the treatment start date, the adverse event start date is imputed with the first day of the month
  - If the year is prior to treatment start date, the adverse event start date is imputed with the last day of the month
  - If the year is after the year of treatment start date, the adverse event start date is imputed with the first day of the month
- UK-UKN-YYYY:
  - If the year is equal to the year of treatment start date, the adverse event start date is imputed with treatment start date of trial 19140B
  - If the year is prior to the year of treatment start date, the adverse event start date is imputed with 31-DEC-YYYY
  - If the year is after the year of treatment start date, the adverse event start date is imputed with 01-JAN-YYYY

If the AE start date is completely missing, it will be set equal to treatment start date of trial 19140B.

Stop Dates

Missing AE stop dates will not be imputed.

Incomplete Intensity Change Dates

If the day is missing in a date of intensity change for an adverse event, the date will be imputed using the same algorithm as described above for incomplete adverse event start dates.

If this results in an imputed start date that is after the stop date of the original event or after an intensity change that comes after the intensity change in question, the start date will be imputed with the stop date of the original event or the date of the later intensity or change.

22.4 Data with Multiple Records

22.4.1 Dose Changes in Medication

Dose changes in medications are recorded on multiple rows in the dataset, with different start and stop dates. When classifying medications into periods, each dose is considered a separate medication, and the same medication can be assigned to several periods for the same participant. Within a period, multiple entries contribute as a single count.

22.4.2 Changes in Intensity or Seriousness of Adverse Events

Changes in adverse event intensity or seriousness are recorded on multiple rows in the dataset. When classifying adverse events into periods, an event will be assigned to more than one period only if the intensity increases or if a non-serious event becomes serious. An adverse event that changes in intensity or seriousness in a period will contribute to the count of events as a single event.

In summaries of adverse events presented by intensity, the maximum intensity of the adverse event will be used. The maximum intensity is searched for in events with changes, as well as over repeated events based on the preferred term. Adverse events for which information on intensity is missing will be classified as *severe*.

Adverse events for which information on seriousness is missing will be classified as *serious*.

References

1. International Headache Society (IHS), Headache Classification Committee. The International Classification of Headache Disorders, 3rd edition 2018 (ICHD-3 2018).
2. Little R and Yau L. Intent-to-treat analysis for longitudinal studies with drop-outs. Biometrics. 1996; 52: 1324-1333.
3. Rubin, DB. Multiple imputation for nonresponse in surveys. New York: Wiley; 1997.
4. Ratitch B, Lipkovich I, O'Kelly M. Combining analysis results from multiply imputed categorical Data. PharmaSUG. 2013; Paper SP03.
5. United States Food and Drug Administration (US FDA). Guidance for Industry: Drug-induced liver injury: premarketing clinical evaluation. July 2009.
6. Kosinski M, Bayliss MS, Bjorner JB, Ware JE, Garber WH, Cady R, et al. A six-item short-form survey for measuring headache impact: the HIT-6. Qual Life Res. 2003; 12(8): 963–974.
7. Jhingran P, Osterhaus JT, Miller DW, Lee JT, Kirchdoerfer L. Development and validation of the Migraine-Specific Quality of Life Questionnaire. Headache. 1998; 38(4): 295–302.
8. Martin BC, Pathak DS, Sharfman MI, Adelman JU, Taylor F, Kwong WJ, Jhingran P. Validity and reliability of the migraine-specific quality of life questionnaire (MSQ Version 2.1). Headache. 2000 Mar; 40(3): 204-215.
9. Mapi Research Trust. Migraine specific quality of life questionnaire. Version 2.1. Scaling and scoring. Version 1.0. November 2017.
10. Herdman M, Gudex C, Lloyd A, Janssen M, Kind P, Parkin D, et al. Development and preliminary testing of the new five-level version of EQ-5D (EQ-5D-5L). Quality of Life Research. 2011; 20: 1727–1736.
11. Reilly MC, Zbrozek AS, Dukes, EM. The validity and reproducibility of a work productivity and activity impairment instrument. Pharmacoeconomics. 1993; 4(5): 353–365.
12. Reilly Associates. WPAI Scoring.[Internet] reillyassociates.net/WPAI_Scoring.html

Appendix I
Statistical Analysis Plan
Authentication and Authorization

Statistical Analysis Plan
Authentication and Authorization

Trial title:	Interventional, open-label, flexible-dose, long-term extension study to evaluate safety of eptinezumab as preventive treatment in participants with migraine in Japan
Trial No.:	19140B
SAP date:	22 August 2024

This document has been signed electronically. The signatories are listed below.

Authentication

Biostatistician: PPD

Clinical research scientist: PPD

Authorization

Head of Biostatistics: PPD

Appendix II
Trial Procedures and Assessments

Trial Procedures and Assessments

Table 1 Trial Procedures and Assessments

**Visit Name**	**Baseline+IMP**	**Phone Contact[c]**	**Phone Contact[u]**	**IMP**	**Phone Contact[c]**	**Phone Contact[u]**	**IMP**	**Phone Contact[c]**	**Phone Contact[u]**	**IMP**	**Phone Contact[c]**	**Phone Contact[u]**	**IMP**	**Phone Contact[c]**	**Phone Contact[u]**	**Completion**	**Safety Follow-up**	**Withdrawal[d]**
**Visit Number**	**1**	**2**	**3**	**4**	**5**	**6**	**7**	**8**	**9**	**10**	**11**	**12**	**13**	**14**	**15**	**16**	**17**	
**End of Week[a]**	**0**	**4**	**8**	**12**	**16**	**20**	**24**	**28**	**32**	**36**	**40**	**44**	**48**	**52**	**56**	**60**	**68**	
**Visit Window[b] (days relative to nominal visit)**		**±2**	**±2**	**±2**	**±2**	**±2**	**±2**	**±2**	**±2**	**±2**	**±2**	**±2**	**±2**	**±2**	**±2**	**±2**	**±5**	
**Baseline Procedures and Assessments**																		
Signed informed consent	√																	
Demographics (age, sex, race)[s]	√																	
Diagnosis[s]	√																	
Inclusion/exclusion criteria	√																	
**Efficacy Assessments (eDiary and PROs)[e, g]**																		
eDiary daily recording[f]	√[s]	√	√	√	√	√	√	√	√	√	√	√	√	√	√	√[i]		
eDiary compliance check[h]	√[s]	√	√	√	√	√	√	√	√	√	√	√	√	√	√	√		
PGI-C	√[s]			√			√			√			√			√		√
MBS	√[s]			√			√			√			√			√		√
**Pharmacoeconomic Assessments <u>(PROs)</u>[g]**																		
HIT-6	√[s]	√	√	√	√	√	√	√	√	√	√	√	√	√	√	√		√
MSQ v2.1	√[s]			√			√			√			√			√		√
EQ-5D-5L	√[s]	√	√	√	√	√	√	√	√	√	√	√	√	√	√	√		√
HCRU	√[s]	√	√	√	√	√	√	√	√	√	√	√	√	√	√	√		√
WPAI:M	√[s]	√	√	√	√	√	√	√	√	√	√	√	√	√	√	√		√
**Pharmacokinetic Assessments**																		
Blood sampling for eptinezumab quantification[t]	√[k,l,s]			√[k,l]			√[k,l]			√[k,l]			√[k,l]			√		√
**Safety Assessments**																		

Visit Name	Baseline+IMP	Phone Contact[c]	Phone Contact[u]	IMP	Phone Contact[c]	Phone Contact[u]	IMP	Phone Contact[c]	Phone Contact[u]	IMP	Phone Contact[c]	Phone Contact[u]	IMP	Phone Contact[c]	Phone Contact[u]	Completion	Safety Follow-up	Withdrawal[d]
**Visit Number**	**1**	**2**	**3**	**4**	**5**	**6**	**7**	**8**	**9**	**10**	**11**	**12**	**13**	**14**	**15**	**16**	**17**	
**End of Week[a]**	**0**	**4**	**8**	**12**	**16**	**20**	**24**	**28**	**32**	**36**	**40**	**44**	**48**	**52**	**56**	**60**	**68**	
**Visit Window[b] (days relative to nominal visit)**		**±2**	**±2**	**±2**	**±2**	**±2**	**±2**	**±2**	**±2**	**±2**	**±2**	**±2**	**±2**	**±2**	**±2**	**±2**	**±5**	
Adverse events	√[j,k,l,s]	√	√	√ j,k,l	√	√	√ j,k,l	√	√	√ j,k,l	√	√	√ j,k,l	√	√	√	√	√
Blood and urine sampling for clinical safety laboratory tests	√[k,s]			√[k]			√[k]			√[k]			√[k]			√		√
Blood sampling for ADA	√[k,s]			√[k]			√[k]			√[k]			√[k]			√	√[v]	√
Vital signs (including body temperature), weight, ECGs	√[k,l,s]			√[k,l]			√[k,l]			√[k,l]			√[k,l]			√		√
Examinations (physical, neurological)	√[m,s]			√[m]			√[m]			√[m]			√[m]			√		√
C-SSRS[n]	√[k,s]			√[k]			√[k]			√[k]			√[k]			√	√	√
**Other Trial Procedures and Assessments**																		
IMP dose adjustment				√[w]														
IMP administered (IV infusion)[o]	√[p]			√[p]			√[p]			√[p]			√[p]					

**Visit Name**	**Baseline+IMP**	**Phone Contact[c]**	**Phone Contact[u]**	**IMP**	**Phone Contact[c]**	**Phone Contact[u]**	**IMP**	**Phone Contact[c]**	**Phone Contact[u]**	**IMP**	**Phone Contact[c]**	**Phone Contact[u]**	**IMP**	**Phone Contact[c]**	**Phone Contact[u]**	**Completion**	**Safety Follow-up**	**Withdrawal[d]**
**Visit Number**	**1**	**2**	**3**	**4**	**5**	**6**	**7**	**8**	**9**	**10**	**11**	**12**	**13**	**14**	**15**	**16**	**17**	
**End of Week[a]**	**0**	**4**	**8**	**12**	**16**	**20**	**24**	**28**	**32**	**36**	**40**	**44**	**48**	**52**	**56**	**60**	**68**	
**Visit Window[b] (days relative to nominal visit)**		**±2**	**±2**	**±2**	**±2**	**±2**	**±2**	**±2**	**±2**	**±2**	**±2**	**±2**	**±2**	**±2**	**±2**	**±2**	**±5**	
IMP accountability[q]	√			√			√			√			√					
Concomitant medication (prescription and non-prescription), traditional Chinese medicines, herbal remedies, non-pharmacological interventions, vitamin and mineral supplements	√[k,s]	√	√	√[k]	√	√	√[k]	√	√	√[k]	√	√	√[k]	√	√	√	√	√
Substance use (alcohol, tobacco, caffeine, marijuana)	√[s]	√	√	√	√	√	√	√	√	√	√	√	√	√	√	√		√
eDiary training/reminder	√																	
PRO training/reminder	√																	
eDiary closeout																√		√
Pregnancy test[r]	√[k,s]			√ [k]			√ [k]			√ [k]			√ [k]			√	√	√

ADA = anti-drug antibody; C-SSRS = Columbia-Suicide Severity Rating Scale; ECG = electrocardiogram; βhCG = beta-human chorionic gonadotropin; EQ-5D-5L = Euroqol 5 Dimensions; HCRU = Health Care Resource Utilization; HIT-6 = Headache Impact Test; IMP = investigational medicinal product; IV = intravenous; MBS = Most Bothersome Symptom; MSQ v2.1 = Migraine-Specific Quality of Life Questionnaire Version 2.1; PGI-C = Patient Global Impression of Change; PRO = participant-reported outcome; SAE = serious adverse event; WD = withdrawal; WPAI:M = Work Productivity and Activity Impairment: Migraine questionnaire

a All assessments may be completed over a maximum of 2 consecutive days except for PROs (see foot note e below); if so the first day is considered the "visit" day according to the schedule.

b If the date of a clinic visit or phone contact does not conform to the schedule, subsequent visits should be planned to maintain the visit schedule relative to the Baseline Visit.

c Participants will be contacted via phone for eDiary compliance check, to ensure PROs have been completed, and for collection of relevant information such as AEs and concomitant medication.

d Participants who withdraw, except for those who withdraw their consent, will be asked to attend a Withdrawal Visit as soon as possible. A Safety Follow-up Visit is scheduled 20 weeks after their last IMP Visit (date when last dose of IMP was administered).

e PROs which are scheduled in alignment with a clinic visit can be completed at the clinic or in the remote setting within 3 days prior to the scheduled clinic visit date. PROs which are scheduled in alignment with a phone contact must be completed in the remote setting and can be completed on the day or within 3 days prior to the scheduled phone contact date.

f The eDiary assessments will be completed in the remote setting on a daily basis.

g At IMP visits, participants must complete the PRO entries prior to infusion. Participants must ensure to complete eDiary recording of headaches that ended prior to infusion (i.e., for headaches which are ongoing or not yet recorded in the eDiary).

h In addition to the eDiary compliance checks performed at the defined clinic visits and phone contacts, ongoing evaluation of eDiary compliance will be performed by the site (based on eDiary reporting) and more frequent contact with participants may be needed in case of non-compliance.

i The eDiary closeout will take place at the Completion Visit/Withdrawal Visit while the participant is at the site. Details will be provided in a separate training module.

j Infusion Related Reactions must be checked as part of the overall AE collection, during and after infusion and before the participant is discharged from the site.

k Infusion must be preceded by the assessment of vital signs including body temperature, concomitant medications, AEs, ECG, blood sampling (for clinical safety laboratory tests, ADA and a pre-infusion PK sample), urine sampling (for clinical safety laboratory and pregnancy tests) and C-SSRS. Vital signs must be assessed prior to blood sampling.

l Vital signs including body temperature and AEs must be checked after infusion. A post-infusion PK sample must be taken within 1 hour after end-of-infusion. Vital signs must be assessed prior to blood sampling.

m Physical and Neurological examinations for all clinic visits are to be conducted at the discretion of the investigator. If the examinations are conducted at the IMP Visit, then these must be performed prior to the infusion.

n The C-SSRS will be administered by the authorized rater at the clinic.

o An unblinded pharmacist or designee is responsible for receiving, storing and preparing IMP. The pharmacist or designee will not be responsible for other aspects of the clinical trial where blinding is necessary.

p Participants must be monitored during the infusion and for a period of 1 hour from the end-of-infusion. Participants will be requested to stay longer should the investigator or designee determine this is clinically warranted.

q A designated unblinded CRA is responsible for the IMP accountability.

r For women of childbearing potential, pregnancy test at the Safety Follow-up Visit is to be conducted using serum β-HCG. At all other visits, urine pregnancy testing will be performed before infusion and in case of a positive finding, further confirmatory testing will be performed via serum β-HCG.

s Assessments performed prior to IMP will be transferred from the Lead-in Trial.

t There will be one blood sample before infusion and another sample within 1 hour after end-of-infusion.

u This phone contact is optional, depending on the ongoing evaluation of eDiary compliance (based on eDiary reporting). In case of non-compliance, a phone contact will be required, and sites must ensure PROs have been completed, and relevant information such as AEs and concomitant medication is collected. If the phone contact is not done, the PROs should be completed as scheduled.

v Participants who test positive for ADA will be asked to provide up to two additional blood samples for immunogenicity testing at 12-week intervals (±1 week) for up to 24 weeks.

w Participants that do not have a treatment response of at least 50% reduction of MMDs by Week 12 (Visit 4) as compared to the Baseline Visit of the Lead-in Trial will have their eptinezumab dose increased to 300 mg.

Appendix III

SAS® Code

SAS® Code

Key Secondary Analysis

The SAS® code for conducting the analysis for the secondary efficacy estimand using Mixed Model for Repeated Measurements as described in Section 12.5.1.2 is given below.

```
proc mixed data = xxxx method=REML;

   class USUBJID AVISITN;

   model CHG = BASE AVISITN BASE*AVISITN / s residual %str(DDRM=KR);

   repeated AVISITN / subject=USUBJID type=UN;

   lsmestimate AVISITN "Change from Baseline in MMDs: Weeks 49-60"
              [1, 13] [1, 14] [1, 15] / divisor = 3 cl;

run;
```

Sensitivity Analysis (Multiple Imputation)

The SAS® code that will be the basis for the MI sensitivity analysis for the secondary endpoints change from Baseline in MMDs (Weeks 49-60), mentioned in Section 12.5.5.1, is given below. Note that data transformation steps are omitted and only the most crucial parts are shown.

The procedure for the MI method will include the following steps:

1. Impute missing intermittent data using Markov Chain Monte Carlo (MCMC) methodology at Baseline and for every 4 Weeks interval (Weeks 1-4, …, Weeks 57-60), using PROC MI from SAS®, with seed = 215428 and 200 imputations, to impute data to a monotone missing data pattern under a MAR assumption.
2. At time t, perform MI, where the monotone missing values are assumed to follow an MNAR pattern. Only observations up to time t will be included. The regression model will include observed or imputed outcomes up to time t (including the Baseline scores), and will use the MONOTON REG option in PROC MI with seed=67653179.
3. Assemble a dataset containing data for all participants, including the imputed data from time t, to serve as predictors for the imputation of the next visit.
4. Repeat steps 2 to 3 sequentially over all visits (t+1, t+2…).
5. Analyse the 200 complete datasets using the same MMRM model as described in Section 12.5.1.2, respectively.
6. Combine the estimated treatment effects obtained across the imputed datasets using PROC MIANALYZE from SAS®.

In the below, it is assumed that:
transposed_data: transposed analysis data with each AVISITN as a separate variable
mono_data: data with monotone missing data pattern
imputed_data: data with missing values imputed
mmrm_data: data with CHG calculated from imputed values and with additional variables to be used in the modelling added
est_data: data with estimated change from baseline from the converged models fit on each of the 200 datasets
results_data: data with the combined estimated treatment effects

Code parts:

```
*Impute intermittent missing values;
proc mi data = transposed_data out = mono_data nimpute = 200 seed = 215428;
    by TRT01P;
    var BASE AVISITN_35 AVISITN_36 AVISITN_37 AVISITN_38 AVISITN_39
AVISITN_40 AVISITN_41 AVISITN_42 AVISITN_43 AVISITN_44 AVISITN_45
AVISITN_46 AVISITN_47 AVISITN_48 AVISITN_49;
    mcmc chain = multiple impute = monotone;
run;

*Impute monotone missing values;
proc mi data = mono_data seed = 67653179 nimpute = 1 out = imputed_data;
    by _IMPUTATION_;
    class TRT01P;
    monotone regression;
    mnar model(AVISITN_35 AVISITN_36 AVISITN_37 AVISITN_38 AVISITN_39
AVISITN_40 AVISITN_41 AVISITN_42 AVISITN_43 AVISITN_44 AVISITN_45
AVISITN_46 AVISITN_47 AVISITN_48 AVISITN_49/ modelobs = (TRT01P = 'EPTI
100/300 mg'));
    var BASE AVISITN_35 AVISITN_36 AVISITN_37 AVISITN_38 AVISITN_39
AVISITN_40 AVISITN_41 AVISITN_42 AVISITN_43 AVISITN_44 AVISITN_45
AVISITN_46 AVISITN_47 AVISITN_48 AVISITN_49;
run;

*Execute model on imputed data;
proc mixed noclprint data = mmrm_data ic method=reml;
    by _IMPUTATION_;
    class USUBJID TRT01P AVISIT;
    model CHG = BASE AVISIT BASE*AVISIT /s ddfm=kr;
    repeated AVISIT /subject=USUBJID type=UN;
    lsmeans AVISIT / diff cl;
    lsmestimate AVISIT "Change from Baseline in MMDs: Weeks 49-60" [1, 13]
[1, 14] [1, 15] / divisor = 3 cl;
    ods output LSMEstimates = est_data;
run;

*Combine model estimates;
proc mianalyze data = est_data;
    modeleffects Estimate;
    stderr StdErr;
    ods output ParameterEstimates = results_data;
run;
```

Appendix IV
PCS Criteria

PCS Criteria

Table 2 PCS Criteria for Clinical Safety Laboratory Tests

Laboratory Test	CDISC Term	Unit	PCS Low	PCS High
**Haematology / Coagulation**				
B-haemoglobin	HGB	g/L	≤ 95 (women)	≥ 165 (women)
			≤ 115 (men)	≥ 185 (men)
B-erythrocytes (red cell count)	RBC	x 10E12/L	≤ 3.5 (women)	≥ 6.0 (women)
			≤ 3.8 (men)	≥ 7.0 (men)
B-haematocrit (packed cell volume)	HCT	V/V	≤ 0.32 (women)	≥ 0.50 (women)
			≤ 0.37 (men)	≥ 0.55 (men)
B-MCV (mean cell volume)	MCV	fL	≤ 0.8 x LLN	≥ 1.2 x ULN
B-total leucocyte (white cell count)	WBC	x 10E9/L	≤ 2.8	≥ 16
B-neutrophils/leucocytes	NEUTLE	%	≤ 20	≥ 85
B-eosinophils/leucocytes	EOSLE	%		≥ 10
B-basophils/leucocytes	BASOLE	%		≥ 10
B-lymphocytes/leucocytes	LYMLE	%	≤ 10	≥ 75
B-monocytes/leucocytes	MONOLE	%		≥ 15
B-thrombocytes (platelet count)	PLAT	x 10E9/L	≤ 75	≥ 600
P-INR (prothrombin ratio)	INR	Ratio		≥ 2.0
B-prothrombin time	PT	Sec		≥ 18
**Liver**				
S-aspartate aminotransferase	AST	U/L		≥ 3 × ULN
S-alanine aminotransferase	ALT	U/L		≥ 3 × ULN
S-bilirubin	BILI	µmol/L		≥ 34
S-bilirubin, direct	BILDIR	µmol/L		≥ 12
S-bilirubin, indirect	BILIND	µmol/L		≥ 22
S-alkaline phosphatase	ALP	U/L		≥ 3 × ULN
S-gamma glutamyl transferase	GGT	U/L		≥ 200
S-alpha-glutathione S-transferase (alpha-GST)	GSTAL	µg/L		≥ 20
**Kidney**				
S-creatinine	CREAT	µmol/L		≥ 1.5 x ULN
B-urea nitrogen (BUN)	BUN	mmol/L		≥ 11
S-uric acid (urate)	URATE	µmol/L		≥ 510 (women)
				≥ 630 (men)
**Electrolytes**				
S-sodium (natrium)	SODIUM	mmol/L	≤ 125	≥ 155
S-potassium (kalium)	K	mmol/L	≤ 3.0	≥ 6.0
S-calcium	CA	mmol/L	≤ 1.8	≥ 3.0

Laboratory Test	CDISC Term	Unit	PCS Low	PCS High
S-chloride	CL	mmol/L	≤ 90	≥ 117
S-magnesium	MG	mmol/L	≤ 0.6	≥ 1.3
S-phosphate (phosphorus, (inorganic)	PHOS	mmol/L	≤ 0.65	≥ 1.95
S-bicarbonate	BICARB	mmol/L	≤ 12	≥ 38
**Endocrine / Metabolic**				
B-glucose, non-fasting/unknown	GLUC	mmol/L	≤ 3.4	≥ 9.4
B-glucose, fasting	GLUC	mmol/L	≤ 3.0	≥ 6.0
S-glucose, non-fasting/unknown	GLUC	mmol/L	≤ 3.9	≥ 11.1
S-glucose, fasting	GLUC	mmol/L	≤ 3.5	≥ 7.0
B-glycosylated haemoglobin, fasting	HBA1C	%		≥ 6.5
S-prolactin	PROLCTN	mIU/L		≥ 1350
S-thyrotropin/TSH	TSH	mIU/L	≤ 0.3	≥ 5.5
S-protein (total)	PROT	g/L	≤ 45	≥ 95
S-albumin	ALB	g/L	≤ 27	
**Lipids**				
S-cholesterol total, non-fasting/unknown	CHOL	mmol/L		≥ 7.8
S-cholesterol total, fasting	CHOL	mmol/L		≥ 6.2
S-triglycerides, non-fasting/unknown	TRIG	mmol/L		≥ 5.65
S-triglycerides, fasting	TRIG	mmol/L		≥ 4.2
S-LDL cholesterol, non-fasting/unknown	LDL	mmol/L		≥ 5.3
S-LDL cholesterol, fasting	LDL	mmol/L		≥ 4.9
S-HDL cholesterol, non-fasting/unknown	HDL	mmol/L	≤ 0.8	
S-HDL cholesterol, fasting	HDL	mmol/L	≤ 0.9	
**Cardiac/Skeletal/Muscle**				
S-creatine kinase (total)	CK	U/L		≥ 400 (women)<br>≥ 750 (men)
S-creatine kinase MB isoenzyme	CKMB	μg/L		≥ 8.5 or
	CKMBCK	%		≥ 3.5% of total CK
S-lactate dehydrogenase (total)	LDH	IU/L		≥ 750
S-troponin I	TROPONI	μg/L		≥ 1.5
S-troponin T	TROPONT	μg/L		≥ 0.4
**Infection**				
S-C-reactive protein	CRP	mg/L		≥ 25
S-globulin (total)	GLOBUL	g/L	≤ 15	≥ 55
**Urine**				
Urinary pH	PH		≤ 4	≥ 9

Laboratory Test	CDISC Term	Unit	PCS Low	PCS High

S=serum; B=whole blood; U=urine

Table 3 PCS Criteria for Vital Signs, Weight/BMI, and Waist Circumference

Variable	CDISC Term	Unit	PCS Low	PCS High
Waist circumference	WSTCIR	Cm	decrease ≥ 7%	increase ≥ 7%
Weight	WEIGHT	Kg	decrease ≥ 7%	increase ≥ 7%
Body Mass Index	BMI	kg/m2	decrease ≥ 7%	increase ≥ 7%
Pulse rate, supine/sitting/unknown	PULSE	beats/min	< 50 and decrease ≥ 15	≥ 120 and increase ≥ 15
Diastolic blood pressure, supine/sitting/unknown	DIABP	mmHg	≤ 50 and decrease ≥ 15	≥ 105 and increase ≥ 15
Systolic blood pressure, supine/sitting/unknown	SYSBP	mmHg	≤ 90 and decrease ≥ 20	≥ 180 and increase ≥ 20
Orthostatic systolic blood pressure	OBP	mmHg	≤ -30	
Orthostatic pulse rate	OPR	beats/min		≥ 20
Temperature	TEMP	ºC	decrease ≥ 2	≥ 38.3 and increase ≥ 2

Increase/decrease is relative to the baseline value.

Table 4 PCS Criteria for ECG Parameters

ECG Parameter	CDISC Term	Unit	PCS Low	PCS High
**Absolute Time Interval**				
PR interval	PRAG	Msec		≥ 260
QRS interval	QRSAG	Msec		≥ 150
QT interval	QTAG	Msec		≥ 500
**Derived Time Interval**				
Heart rate	EGHRMN	beats/min	< 50 and decrease ≥ 15	≥ 120 and increase ≥ 15
QTcB interval	QTCBAG	Msec	< 300	> 500 or increase > 60
QTcF interval	QTCFAG	Msec	< 300	> 500 or increase > 60

Increase/decrease is relative to the Baseline value.

Appendix V
Adverse Events of Special Interest

Adverse Events of Special Interest

Table 5 Adverse Events of Special Interest

Event types	SMQ/HLT/HLGT	Additional criteria
Cardio/cerebrovascular events	Cardiac arrhythmias (SMQ) (Narrow)	
	Cardiac failure (SMQ) (Narrow)	
	Cardiomyopathy (SMQ) (Narrow)	
	Central nervous system vascular disorders (SMQ) (Narrow)	
	Embolic and thrombotic events (SMQ) (Narrow)	
	Hypertension (SMQ) (Narrow)	
	Ischaemic heart disease (SMQ) (Narrow)	
	Pulmonary hypertension (SMQ) (Narrow)	
	Torsade de pointes/QT prolongation (SMQ) (Narrow)	
Events associated with Suicide	Suicide/self-injury (SMQ) (Narrow)	
Events potentially associated with Trial Drug Infusion	Angioedema and urticaria (HLGT) (primary PTs only)	TEAE on the day of dosing after the infusion was started or during the 7 days after dosing
	Bronchial disorders (excl neoplasms) (HLGT) (primary PTs only)	TEAE on the day of dosing after the infusion was started
	Infusion site reactions (HLT) (primary PTs only)	TEAE on the day of dosing after the infusion was started or during the 7 days after dosing
	Oral soft tissue signs and symptoms (HLT) (primary PTs only)	TEAE on the day of dosing after the infusion was started
	Oral soft tissue swelling and oedema (HLT) (primary PTs only)	TEAE on the day of dosing after the infusion was started
	Pruritus NEC (HLT) (primary PTs only)	TEAE on the day of dosing after the infusion was started or during the 7 days after dosing
	Rashes, eruptions and exanthems NEC (HLT) (primary PTs only)	TEAE on the day of dosing after the infusion was started or during the 7 days after dosing
	Respiratory disorders NEC (HLGT) (primary PTs only)	TEAE on the day of dosing after the infusion was started
	Respiratory tract signs and symptoms (HLGT) (primary PTs only)	TEAE on the day of dosing after the infusion was started
	Upper respiratory tract disorders (excl infections) (HLGT) (primary PTs only)	TEAE on the day of dosing after the infusion was started

Event types	SMQ/HLT/HLGT	Additional criteria
Hepatic events	Drug related hepatic disorders - comprehensive search (SMQ) (Narrow)	
Hypersensitivity and Anaphylactic Reactions	Anaphylactic reaction (SMQ) (Narrow)<br>Angioedema (SMQ) (Narrow)<br>Hypersensitivity (SMQ) (Narrow)	
Seizures	Convulsions (SMQ) (Narrow)	
