# Supplementary material for: Long-term tolerability and effectiveness of eptinezumab in Japanese adults with chronic migraine: results of the 60-week open-label SUNSET trial
Source: J Headache Pain. 2025 Nov 22;26(1):275. doi: 10.1186/s10194-025-02214-w (PMC12659320; doi:10.1186/s10194-025-02214-w)
Supplement: Supplementary file 3 — Additional file 3 [file 10194_2025_2214_MOESM3_ESM.pdf]

SUPPLEMENTARY MATERIAL

Supplementary Table 1. Summary of effectiveness endpoints

Supplementary Table 2. PI-MBS category at baseline

Supplementary Figure 1. Change from baseline in MMDs according to treatment in SUNRISE

Supplementary Figure 2. Change from baseline in MSQ v2.1 domain scores: (a) role function–restrictive, (b) role function–preventive, and (c) emotional function

Supplementary Table 1. Summary of effectiveness endpoints

	Eptinezumab 100/300 mg (N = 158)			
**Efficacy measures**	**SUNRISE Baseline**	**SUNSET Weeks 1–12**	**SUNSET Weeks 13–24**	**SUNSET Weeks 49–60**
**MMDs**, N	158	158	156	143
Mean baseline score/LS mean (SE) change from baseline during interval	17.1	−4.4 (0.5)	−5.0 (0.5)	−5.4 (0.6)
**≥50% migraine responder rate**, n (%)	–	46 (29.1%)	49 (31.4%)	51 (35.7%)
**≥75% migraine responder rate**, n (%)	–	11 (7.0%)	17 (10.9%)	25 (17.5%)
**ePROs**	**SUNRISE Baseline**	**SUNSET Week 12**	**SUNSET Week 24**	**SUNSET Week 60**
**PGIC**, N	158	158	152	139
Mean baseline score/LS mean (SE) score at timepoint	4.0	3.0 (0.1)	2.7 (0.1)	2.5 (0.1)
Responders[a], n (%)	–	53 (33.5%)	78 (51.3%)	75 (54.0%)
**PI-MBS**, N	158	157	151	138
Mean baseline score/LS mean (SE) score at timepoint	4.0	3.1 (0.1)	2.7 (0.1)	2.6 (0.1)
Responders[a], n (%)	–	55 (35.0%)	75 (49.7%)	74 (53.6%)
**HIT-6 total score**, N	154	158	150	139
Mean baseline score/LS mean (SE) change from baseline at timepoint	64.3	−5.8 (0.6)	−7.6 (0.6)	−8.8 (0.6)
**EQ-5D-5L VAS**, N	151	151	142	132
Mean baseline score/LS mean (SE) change from baseline at timepoint	71.0	−0.3 (1.7)	1.6 (1.7)	2.9 (2.0)
**MSQ v2.1 domain: role function-restrictive**, N	151	151	145	132
Mean baseline score/LS mean (SE) change from baseline at timepoint	51.9	15.6 (1.4)	18.7 (1.5)	22.2 (1.4)
**MSQ v2.1 domain: role function-preventive**, N	151	151	145	132
Mean baseline score/LS mean (SE) change from baseline at timepoint	71.1	12.0 (1.2)	12.8 (1.3)	15.6 (1.2)
**MSQ v2.1 domain: emotional function**, N	151	151	145	132
Mean baseline score/LS mean (SE) change from baseline at timepoint	62.7	10.4 (1.6)	13.9 (1.5)	17.5 (1.6)
**WPAI:M domain: absenteeism**, N	110	99	94	88
Mean baseline score/LS mean (SE) change from baseline at timepoint	3.6	1.6 (1.3)	−1.6 (0.7)	0.6 (1.1)
**WPAI:M domain: presenteeism**, N	109	98	93	88
Mean baseline score/LS mean (SE) change from baseline at timepoint	50.4	−9.0 (2.4)	−17.2 (2.5)	−17.2 (2.1)
**WPAI:M domain: work productivity loss**, N	109	98	93	88
Mean baseline score/LS mean (SE) change from baseline at timepoint	51.3	−8.1 (2.5)	−17.0 (2.6)	−16.0 (2.3)
**WPAI:M domain: activity impairment**, N	151	151	141	132
Mean baseline score/LS mean (SE) change from baseline at timepoint	56.6	−12.3 (2.0)	−18.6 (2.1)	−21.1 (2.1)

Data presented here are from the full analysis set. Baseline values are from the SUNRISE trial.

[a]Participants responding ‘very much improved’ or ‘much improved’.

ePROs, electronic patient-reported outcomes; HIT-6, 6-item Headache Impact Test; LS, least-squares; MMDs, monthly migraine days; MSQ v2.1, Migraine-Specific Quality-of-Life Questionnaire, version 2.1; PGIC, Patient Global Impression of Change; PI-MBS, patient-identified most bothersome symptom; SE, standard error; WPAI, Migraine-specific Work Productivity and Activity Impairment questionnaire; VAS, visual analog scale.

Supplementary Table 2. PI-MBS category at baseline

	**Eptinezumab 100/300 mg** (N = 158)
**Participants who specified their most bothersome symptom at baseline**, n	157
**Most bothersome symptom**, n (%)	
Pain with activity	56 (35.7%)
Nausea	28 (17.8%)
Fatigue	23 (14.6%)
Sensitivity to light	13 (8.3%)
Sensitivity to sounds	10 (6.4%)
Mood changes	6 (3.8%)
Vomiting	2 (1.3%)
Other	19 (12.1%)

Data presented here are from the full analysis set. Baseline values are from the SUNRISE trial. PI-MBS, patient-identified most bothersome symptom.

Supplementary Figure 1. Change from baseline in MMDs according to treatment in SUNRISE

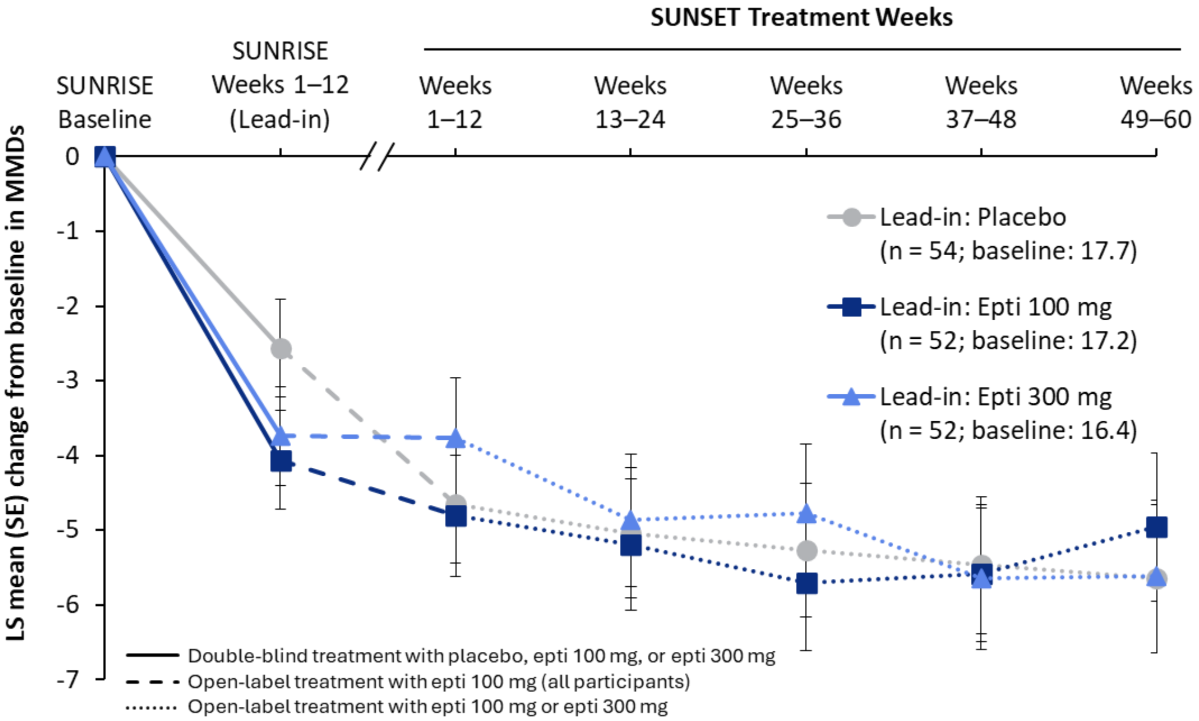

Data presented here are from the full analysis set. During Weeks 1–12 of the SUNSET trial, all participants were treated with eptinezumab 100 mg, and during Weeks 13–60, all participants received eptinezumab 100 or 300 mg based their ≥50% responder status in Weeks 1–12 of SUNSET. The estimated LS means were derived from an MMRM with month and prior treatment group as factors, baseline score as a continuous covariate, prior treatment group-by-month interaction, and baseline score-by-month interaction. From the MMRM, estimates across multiple 4-week intervals were computed using equal weights for each 4-week interval.

Epti, eptinezumab; LS, least-squares; MMDs, monthly migraine days; MMRM, mixed model for repeated measures; SE, standard error.

Supplementary Figure 2. Change from baseline in MSQ v2.1 domain scores: (a) role function–restrictive, (b) role function–preventive, and (c) emotional function

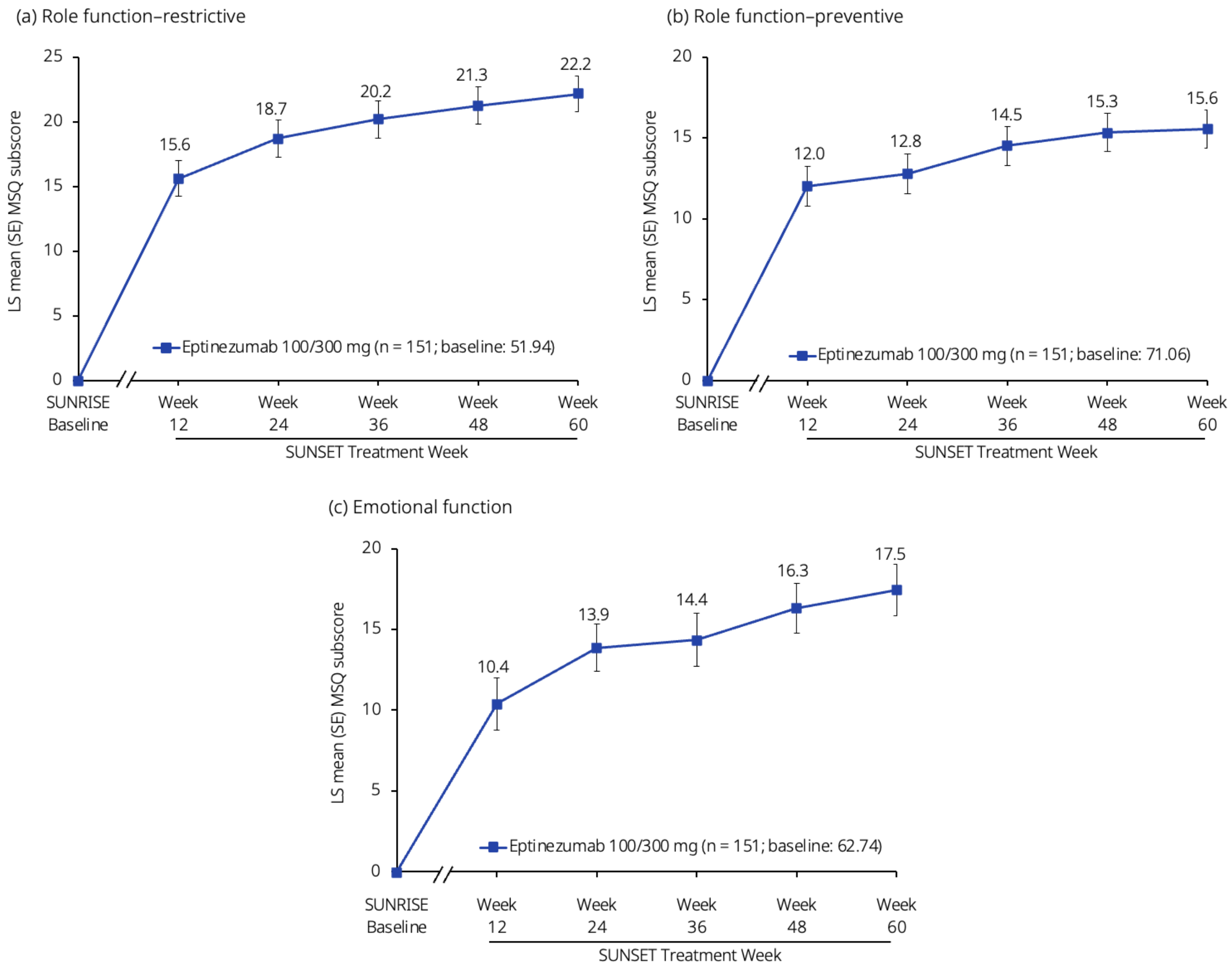

Data presented here are from the full analysis set. The axis break between SUNRISE baseline and SUNSET Week 12 represents the 12-week placebo-controlled period in SUNRISE that preceded the SUNSET trial. The MSQ v2.1 consists of 14 items covering three domains: role function restrictive (7 items); role function preventive (4 items); and emotional function (3 items). Each item is scored on a 6-point scale ranging from 1 (none of the time) to 6 (all of the time). Raw domain scores are summed and transformed to a 0- to 100-point scale. Higher scores indicate better quality of life.

The estimated LS means were derived from a mixed model for repeated measure with visit as a factor, baseline score as a continuous covariate, and baseline score-by-month interaction.

LS, least-squares; MSQ v2.1, Migraine-Specific Quality-of-Life Questionnaire, version 2.1; SE, standard error.
